# Ancient pigs reveal a near-complete genomic turnover following their introduction to Europe

**DOI:** 10.1073/pnas.1901169116

**Published:** 2019-08-12

**Authors:** Laurent A. F. Frantz, James Haile, Audrey T. Lin, Amelie Scheu, Christina Geörg, Norbert Benecke, Michelle Alexander, Anna Linderholm, Victoria E. Mullin, Kevin G. Daly, Vincent M. Battista, Max Price, Kurt J. Gron, Panoraia Alexandri, Rose-Marie Arbogast, Benjamin Arbuckle, Adrian Bӑlӑşescu, Ross Barnett, László Bartosiewicz, Gennady Baryshnikov, Clive Bonsall, Dušan Borić, Adina Boroneanţ, Jelena Bulatović, Canan Çakirlar, José-Miguel Carretero, John Chapman, Mike Church, Richard Crooijmans, Bea De Cupere, Cleia Detry, Vesna Dimitrijevic, Valentin Dumitraşcu, Louis du Plessis, Ceiridwen J. Edwards, Cevdet Merih Erek, Aslı Erim-Özdoğan, Anton Ervynck, Domenico Fulgione, Mihai Gligor, Anders Götherström, Lionel Gourichon, Martien A.M. Groenen, Daniel Helmer, Hitomi Hongo, Liora K. Horwitz, Evan K. Irving-Pease, Ophélie Lebrasseur, Joséphine Lesur, Caroline Malone, Ninna Manaseryan, Arkadiusz Marciniak, Holley Martlew, Marjan Mashkour, Roger Matthews, Giedre Motuzaite Matuzeviciute, Sepideh Maziar, Erik Meijaard, Tom McGovern, Hendrik-Jan Megens, Rebecca Miller, Azadeh Fatemeh Mohaseb, Jörg Orschiedt, David Orton, Anastasia Papathanasiou, Mike Parker Pearson, Ron Pinhasi, Darko Radmanović, François-Xavier Ricaut, Mike Richards, Richard Sabin, Lucia Sarti, Wolfram Schier, Shiva Sheikhi, Elisabeth Stephan, John R. Stewart, Simon Stoddart, Antonio Tagliacozzo, Nenad Tasić, Katerina Trantalidou, Anne Tresset, Cristina Valdiosera, Youri van den Hurk, Sophie Van Poucke, Jean-Denis Vigne, Alexander Yanevich, Andrea Zeeb-Lanz, Alexandros Triantafyllidis, M. Thomas P. Gilbert, Jörg Schibler, Peter Rowley-Conwy, Melinda Zeder, Joris Peters, Thomas Cucchi, Daniel G. Bradley, Keith Dobney, Joachim Burger, Allowen Evin, Linus Girdland-Flink, Greger Larson

**Affiliations:** ^a^School of Biological and Chemical Sciences, Queen Mary University of London, London E1 4NS, United Kingdom;; ^b^The Palaeogenomics & Bio-Archaeology Research Network, Research Laboratory for Archaeology and History of Art, University of Oxford, Oxford OX1 3TG, United Kingdom;; ^c^Department of Zoology, University of Oxford, Oxford OX1 3SZ, United Kingdom;; ^d^Palaeogenetics Group, Institute of Organismic and Molecular Evolution, Johannes Gutenberg-University Mainz, D-55128 Mainz, Germany;; ^e^Department of Natural Sciences, German Archaeological Institute, 14195 Berlin, Germany;; ^f^BioArCh, Department of Archaeology, University of York, York YO10 5NG, United Kingdom;; ^g^Department of Anthropology, Texas A&M University, College Station, TX 77840;; ^h^Department of Earth Sciences, Natural History Museum, London SW7 5BD, United Kingdom;; ^i^Molecular Population Genetics, Smurfit Institute of Genetics, Trinity College Dublin, Dublin 2, Ireland;; ^j^Department of Anthropology, University of Michigan, Ann Arbor, MI 48109;; ^k^Department of Materials Science and Engineering, Massachusetts Institute of Technology, Cambridge, MA 02142;; ^l^Department of Archaeology, Durham University, Durham DH1 3LE, United Kingdom;; ^m^CNRS UMR 7044, Maison interuniversitaire des sciences de l’Homme, F-67083 Strasbourg Cedex, France;; ^n^Department of Genetics, Development and Molecular Biology, School of Biology, Aristotle University of Thessaloniki, 54124 Thessaloniki, Greece;; ^o^Department of Anthropology, University of North Carolina at Chapel Hill, Chapel Hill, NC 27599;; ^p^“Vasile Pârvan” Institute of Archaeology, Bucharest 010667, Romania;; ^q^Osteoarchaeological Research Laboratory, Department of Archaeology and Classical Studies, Stockholm University, 106 91 Stockholm, Sweden;; ^r^Laboratory of Theriology, Zoological Institute of the Russian Academy of Sciences, St. Petersburg 199034, Russia;; ^s^School of History, Classics and Archaeology, University of Edinburgh, Edinburgh EH8 9AG, United Kingdom;; ^t^The Italian Academy for Advanced Studies in America, Columbia University, New York, NY 10027;; ^u^Laboratory for Bioarchaeology, Department of Archaeology, Faculty of Philosophy, University of Belgrade, 11000 Belgrade, Serbia;; ^v^Institute of Archaeology, University of Groningen, 9712 ER, Groningen, The Netherlands;; ^w^Laboratorio de Evolución Humana, Departamento de Historia, Geografía y Comunicación Universidad de Burgos, Burgos, Spain;; ^x^Animal Breeding and Genomics Center, Wageningen University and Research, 6708 PB Wageningen, The Netherlands;; ^y^OD Earth and History of Life, Royal Belgian Institute of Natural Sciences, 1000 Brussels, Belgium;; ^z^Centro de Arqueologia da Universidade de Lisboa, Faculdade de Letras da Universidade de Lisboa, Alameda da Universidade, 1600-214 Lisboa, Portugal;; ^aa^Department of Biological and Geographical Sciences, University of Huddersfield, Huddersfield HD1 3DH, United Kingdom;; ^bb^Department of Archaeology, Gazi University, Ankara 06500, Turkey;; ^cc^Department of Archaeology, Çanakkale Onsekiz Mart University, Çanakkale 17100, Turkey;; ^dd^Flanders Heritage Agency, 1000 Brussels, Belgium;; ^ee^Department of Biology, University of Naples Federico II, 80126 Napoli, Italy;; ^ff^History, Archaeology and Museology Department, 1 Decembrie 1918 University, Alba Iulia 510009, Romania;; ^gg^Department of Archaeology and Classical Studies, Stockholm University, SE-106 91 Stockholm, Sweden;; ^hh^Université Côte d’Azur, CNRS, Cultures et Environnement, Préhistoire, Antiquité, Moyen Âge (UMR 7264), 06357 Nice, France;; ^ii^CNRS, Archéorient (UMR 5133), Maison de l’Orient et de la Méditerranée, 69007 Lyon, France;; ^jj^Department of Evolutionary Studies of Biosystems, Graduate University for Advanced Studies, Hayama, Kanagawa 240-0193, Japan;; ^kk^National Natural History Collections, Faculty of Life Science, The Hebrew University of Jerusalem, Jerusalem 91904, Israel;; ^ll^Department of Archaeology, Classics and Egyptology, University of Liverpool, Liverpool L69 7WZ, United Kingdom;; ^mm^Unité Archéozoologie, Archéobotanique, Sociétés Pratiques et Environnements (AASPE), CNRS, Muséum National d’Histoire Naturelle, 75020 Paris, France;; ^nn^School of Natural and Built Environment, Queen’s University Belfast, Belfast BT9 5AG, United Kingdom;; ^oo^Scientific Center of Zoology and Hydroecology, Institute of Zoology, Yerevan 0014, Armenia;; ^pp^Institute of Archaeology, Adam Mickiewicz University, 61-712, Poznań, Poland;; ^qq^The Hellenic Archaeological Research Foundation, Tivoli House, Cheltenham GL50 2TD, United Kingdom;; ^rr^Department of Archaeology, University of Reading, Reading RG6 6AB, United Kingdom;; ^ss^Lithuanian Institute of History, Vilnius University, LT-01513 Vilnius, Lithuania;; ^tt^Institut für Archäologische Wissenschaften, Goethe University of Frankfurt, 60323 Frankfurt, Germany;; ^uu^International Union for Conservation of Nature/Species Survival Commission Wild Pig Specialist Group, 15412 Jakarta, Indonesia;; ^vv^Center of Excellence for Environmental Decisions, University of Queensland, St Lucia, QLD 4072, Australia;; ^ww^Durrell Institute of Conservation and Ecology, School of Anthropology and Conservation, Marlowe Building, University of Kent, Canterbury, Kent CT2 7NR, United Kingdom;; ^xx^Anthropology Department, Hunter College and Graduate Center, City University of New York, New York, NY 10065;; ^yy^Service de Préhistoire, Université de Liège, 4000 Liège, Belgium;; ^zz^Institute of Prehistoric Archaeology, Free University of Berlin, 14195 Berlin, Germany;; ^aaa^Curt-Engelhorn-Zentrum Archäometrie, 68159 Mannheim, Germany;; ^bbb^Ephorate of Paleoanthropology and Speleology, Greek Ministry of Culture, 106 82 Athens, Greece;; ^ccc^Institute of Archaeology, University College London, London WC1H 0PY, United Kingdom;; ^ddd^Department of Evolutionary Anthropology, University of Vienna, 1090 Vienna, Austria;; ^eee^Museum of Vojvodina, 21101 Novi Sad, Serbia;; ^fff^Laboratoire Évolution & Diversité Biologique-UMR 5174, Université de Toulouse Midi-Pyrénées, 31062 cedex 9 Toulouse, France;; ^ggg^Department of Archaeology, Simon Fraser University, Burnaby, BC V5A 1S6, Canada;; ^hhh^Division of Vertebrates, Department of Life Sciences, The Natural History Museum, London SW7 5BD, United Kingdom;; ^iii^Dipartimento di Scienze storiche e dei Beni Culturali, University of Siena, 53100 Siena, Italy;; ^jjj^Osteologie, Landesamt für Denkmalpflege im Regierungspräsidium Stuttgart, 73728 Konstanz, Germany;; ^kkk^Faculty of Science and Technology, Bournemouth University, Fern Barrow, Poole, Dorset BH12 5BB, United Kingdom;; ^lll^Magdalene College, University of Cambridge, Cambridge CB3 0AG, United Kingdom;; ^mmm^Sezione di Bioarcheologia, Museo delle Civiltà, 00144 Roma, Italy;; ^nnn^Department of Archaeology, Faculty of Philosophy, University of Belgrade, 11000 Belgrade, Serbia;; ^ooo^Department of Archaeology and History, Faculty of Humanities and Social Sciences, La Trobe University, Melbourne, MB 167, Australia;; ^ppp^Institute of Archaeology of the National Academy of Sciences of Ukraine, 02000 Kiev, Ukraine;; ^qqq^Generaldirektion Kulturelles Erbe Rheinland-Pfalz, Dir. Landesarchäologie, D–67346 Speyer, Germany;; ^rrr^Natural History Museum of Denmark, University of Copenhagen, DK-1123 Copenhagen, Denmark;; ^sss^University Museum, Norwegian University of Science and Technology, 7012 Trondheim, Norway;; ^ttt^Integrative Prehistory and Archaeological Science, University of Basel, 4055 Basel, Switzerland;; ^uuu^Department of Anthropology, National Museum of Natural History, Smithsonian Institution, Washington, DC 37012;; ^vvv^ArchaeoBioCenter and Department of Veterinary Sciences, Institute of Palaeoanatomy, Domestication and the History of Veterinary Medicine, Ludwig Maximilian University Munich, 80539 Munich, Germany;; ^www^State Collection for Anthropology and Palaeoanatomy, Bavarian Natural History Collections, 80333 Munich, Germany;; ^xxx^Department of Archaeology, School of Geosciences, University of Aberdeen, St. Mary’s, Aberdeen AB24 3FUK, United Kingdom;; ^yyy^Institut des Sciences de l’Evolution-Montpellier-UMR 5554-CNRS, IRD, Université de Montpellier, 34090 Montpellier, France;; ^zzz^Research Centre in Evolutionary Anthropology and Palaeoecology, School of Natural Sciences and Psychology, Liverpool John Moores University, Liverpool L3 3AF, United Kingdom

**Keywords:** domestication, evolution, gene flow, Neolithic

## Abstract

Archaeological evidence indicates that pig domestication had begun by ∼10,500 y before the present (BP) in the Near East, and mitochondrial DNA (mtDNA) suggests that pigs arrived in Europe alongside farmers ∼8,500 y BP. A few thousand years after the introduction of Near Eastern pigs into Europe, however, their characteristic mtDNA signature disappeared and was replaced by haplotypes associated with European wild boars. This turnover could be accounted for by substantial gene flow from local European wild boars, although it is also possible that European wild boars were domesticated independently without any genetic contribution from the Near East. To test these hypotheses, we obtained mtDNA sequences from 2,099 modern and ancient pig samples and 63 nuclear ancient genomes from Near Eastern and European pigs. Our analyses revealed that European domestic pigs dating from 7,100 to 6,000 y BP possessed both Near Eastern and European nuclear ancestry, while later pigs possessed no more than 4% Near Eastern ancestry, indicating that gene flow from European wild boars resulted in a near-complete disappearance of Near East ancestry. In addition, we demonstrate that a variant at a locus encoding black coat color likely originated in the Near East and persisted in European pigs. Altogether, our results indicate that while pigs were not independently domesticated in Europe, the vast majority of human-mediated selection over the past 5,000 y focused on the genomic fraction derived from the European wild boars, and not on the fraction that was selected by early Neolithic farmers over the first 2,500 y of the domestication process.

## Significance

Archaeological evidence indicates that domestic pigs arrived in Europe, alongside farmers from the Near East ∼8,500 y ago, yet mitochondrial genomes of modern European pigs are derived from European wild boars. To address this conundrum, we obtained mitochondrial and nuclear data from modern and ancient Near Eastern and European pigs. Our analyses indicate that, aside from a coat color gene, most Near Eastern ancestry in the genomes of European domestic pigs disappeared over 3,000 y as a result of interbreeding with local wild boars. This implies that pigs were not domesticated independently in Europe, yet the first 2,500 y of human-mediated selection applied by Near Eastern Neolithic farmers played little role in the development of modern European pigs.

The emergence of agricultural societies in the Near East at least 12,500 y before the present (BP) was followed by the westward dispersal of farmers into Europe beginning ∼8,500 y BP (1–4). This Neolithic expansion was characterized by the human-mediated dispersal of domesticated plants and animals, including cereals, pulses, sheep, goats, cattle, and pigs, all of which were derived from wild species indigenous to the Near East and Anatolia (5, 6). Given that the wild progenitors of modern domestic sheep and goats were never present in Europe, the presence of their remains in European archaeological sites almost certainly represents populations originally domesticated in Anatolia and the Near East. In the case of cattle and pigs, however, the widespread distribution of their wild progenitors across most of Eurasia complicates the classification of archaeological specimens as wild or domestic, and leaves open the possibility that these taxa were also independently domesticated in Europe. Consequently, the relative contribution of European wild boars populations to the gene pools of domestics introduced from the Near East remains contentious (7).

Traditional methods for distinguishing between wild and domestic pigs rely primarily on archaeological context and size differences (8) or are based on demographic profiling (9, 10). More recent methods have relied on the analysis of dental shape variation using geometric morphometrics (11, 12) and stable isotopes (13). Morphological analyses of archeological pig remains have indicated that the first domestic pigs introduced from the Near East were substantially smaller than European wild boars, something most clearly visible in tooth size (e.g., ref. 14). Dental development is generally unaffected by nutrition until extreme starvation approaches (15), and tooth size is slow to change. For example, Australian feral pigs whose ancestors have been living outside of anthropogenic contexts for as long as 2 centuries still possess the small tooth sizes of their domestic ancestors, even though their body size has substantially enlarged (16). In Europe, the earliest domesticated pigs (identified using tooth size) have been recovered from archaeological contexts associated with the earliest Neolithic farmers by ∼8,000 y BP (e.g., ref. 14), and these tooth size differences persist from prehistory to the present day (8, 17). Thus, the archaeological evidence implies that none of the *Sus scrofa* present in Europe before the arrival of Near Eastern farmers can be classified as domestic, indicating that European hunter-gatherers did not independently domesticate local wild boars.

Although the phenotype associated with Near Eastern domestic pigs does not appear to vary considerably following their introduction to Europe (18–20), there is substantial discontinuity with respect to their maternal (mitochondrial DNA [mtDNA]) ancestry. Ancient mtDNA analysis has shown that pigs of Near Eastern maternal ancestry occurred as far west as the Paris Basin (∼6,000 y BP) among early Neolithic European domestic pigs (21). By 5,900 y BP, however, these Near Eastern genetic signatures had been replaced by those of European wild boars (21), and it is possible that the Near Eastern ancestry also vanished from the nuclear genome of modern domestic pigs. A recent analysis of ∼37,000 single-nucleotide polymorphisms (SNPs) typed in modern pigs (22) was consistent with this hypothesis, but this study was likely underpowered due to ascertainment biases and a lack of ancient Near Eastern domestic and wild reference populations.

One possible mechanism to account for the apparent discontinuity between genotype and phenotype is gene flow from local European wild boars into the introduced domestic population. Domestic pigs have likely always interacted and interbred with wild populations, and this process has been suggested wherever domestic animals have arrived (e.g., ref. 23). Genetic introgression (including the mitochondrial genome) from local wild boars into the introduced domestic population potentially involved wild females being captured [perhaps as piglets during hunting as in modern New Guinea (24, 25)] and kept in farming settlements. Were these females allowed to reach sexual maturity and breed with male domestics, the offspring would possess mtDNA (and some nuclear ancestry) associated with local wild boars. Although perhaps initiated as an accident, if the offspring of the wild-caught females were perceived to possess superior traits, the acquisition of wild female piglets may have become a regular practice.

If this admixture was limited (at least initially), and the gene flow from wild boars did not substantially affect the phenotype of the domestic population, it is possible that modern domestic pigs retain a sufficient, yet undetected, fraction of Near Eastern ancestry that underlies domestic traits (26). This scenario of continuous gene flow with European wild boars predicts a gradual and incomplete genomic replacement. If pig domestication was a completely independent process, European pigs would derive exclusively from European wild boars, resulting in a sharp discontinuity of Near Eastern ancestry.

Here, we assessed whether modern domestic pig genomes retain a Near Eastern component that is essential for maintaining their domestic characteristics, and characterized the extent, speed, and mechanisms by which pigs acquired European wild boars ancestry. To do so, we obtained mitochondrial (including PCR data [*n* = 230] and next-generation sequencing (NGS) data [*n* = 327]) and nuclear data, including 2 high-coverage (>10-fold), 7 medium-coverage (1- to 10-fold), and 54 low-coverage (<1-fold) genomes from an assessment of >500 archeological pig remains (Dataset S1). Our dataset (including publicly available sequences) spans the past 14,000 y and includes a total of 2,099 samples from the Near East and Europe, including samples from contexts that precede and follow the origins of pig domestication.

## Results and Discussion

### A Neolithic Mitochondrial Turnover.

**Fig. 1.**
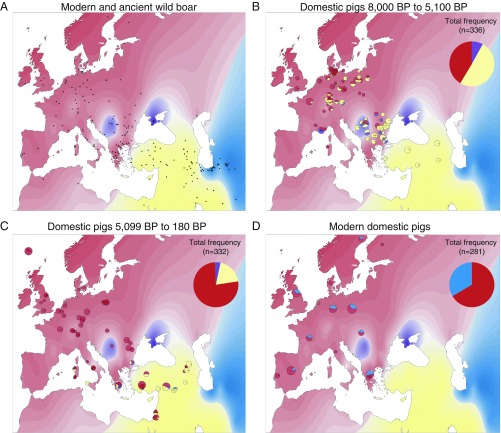
(*A*) Map representing the distribution of East Asian (blue), Near Eastern (including haplogroups mt-Y1 and mt-ArmT; yellow), European (including haplogroups mt-Italian, mt-A, mt-C, and mt-Y2; red), and Y2 (purple) haplogroups in wild boars. Black dots represent the locations of 696 modern and ancient wild boar. Haplogroup assignments were used to interpolate the underlying color distribution, which demonstrates the biogeographical boundaries of these 3 general haplogroups. (*B*) Large pie chart in the upper right corner of the map represents overall frequencies of these haplogroups in domestic pigs. Small pie charts on the map show the frequencies at various archeological sites/locations between 8,000 y BP and 5,100 y BP (*B*), between 5,099 and 180 y BP [before the Industrial Revolution and the introduction of Asian pigs in Europe (35) (*C*), and in modern pigs (*D*)]. A few samples from our datasets have been excluded from these plots; more details are provided in *SI Appendix*, Figs. S6 and S7.

Our mtDNA analysis revealed 2 broad groups: 1 from Western and Eastern Europe, including mt-Italian, mt-A, mt-C, and mt-Y2 haplogroups (Fig. 1*A* and *SI Appendix*, Figs. S7 and S8), and 1 from the Near East, including haplogroups mt-Y1 and mt-ArmT (Fig. 1*A* and *SI Appendix*, Figs. S7 and S8). These results substantiate previous findings that mt-Y1 and mt-ArmT are indigenous to the Near East, although mt-Y2, previously thought to be found exclusively in the Near East (21), also appears to be present in wild boars from the Balkans and northeast Italy (19, 27) (*SI Appendix*). In addition, the mt-Y1 signature, originally restricted to the Near East (Fig. 1*A*), was not only identified in early Neolithic contexts in the Near East and Europe but was also found in pigs that (based on context and traditional biometrical analysis) were assigned a domestic status (21, 28) (*SI Appendix*).

Altogether, this confirms that Near Eastern farmers brought domestic pigs possessing an mt-Y1 signature into Europe during the Neolithic expansion (21, 28). Our analysis of mtDNA data from 2,099 samples (557 newly generated data), including 1,318 ancient samples (262 of wild boars, 592 of domestic pigs, and 464 of unknown status) and 781 modern samples (467 of wild boars and 314 of domestic pigs), demonstrates that the first appearance of the mt-Y1 haplotype in our continental European dataset was ∼8,000 y ago in Neolithic Bulgarian pigs (Kovačevo: Kov18, Kov21), and its terminal appearance in a Neolithic context was ∼5,100 y ago in a Polish sample (AA134, Żegotki 2).

The few pigs possessing an mt-Y1 signature from post-Neolithic contexts were found mostly on islands beyond mainland Europe in southwestern Greece (4,350 to 3,250 y BP: MM495, MM486, MM303), in Crete (3,100 y BP), in Sardinia (∼3,750 y BP) (29), near Naples (∼800 y BP: VM_CM01, VM_CM02, VM_CM03), and in Corsica (modern noncommercial pigs) (21), as well as in Tuscany (∼800 y BP: VM_TM01) (Fig. 1 and *SI Appendix*). The persistence of the mt-Y1 signature within pigs on islands mimics the patterns seen in isolated island populations of both sheep and humans. For instance, sheep in Orkney and St. Kilda (30), and human populations in Sardinia (31), were not subjected to significant introgression from later migratory waves and, instead, possess a larger proportion of Anatolian/Near Eastern ancestry relative to their mainland counterparts.

### Gene Flow and a Corresponding Near-Complete Nuclear Turnover.

**Fig. 2.**
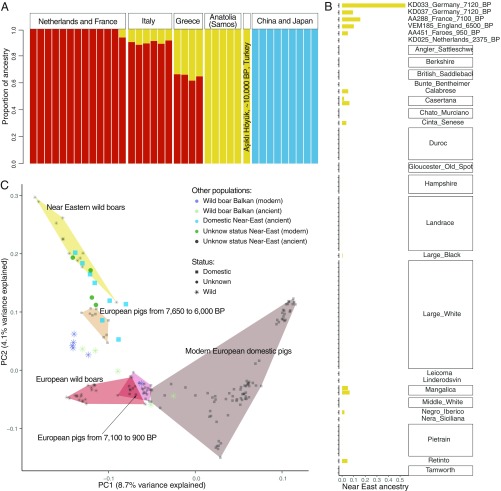
(*A*) Bar plots representing the proportion of ancestry from Europe (red), the Near East (yellow), and East Asia (blue) in Eurasian wild boar genomes. (*B*) Bar plots depicting the proportion of Near Eastern ancestry in modern and ancient European domestic pigs. (*C*) PCA (excluding East Asian domestic pigs; *SI Appendix*, Fig. S14) showing the existence of 2 groups of ancient domestic pigs: 1 close to Near Eastern wild boar and 1 close to European wild boar.

While these data confirm the existence of a complete turnover of mtDNA, this marker does not provide sufficient power to assess whether the turnover was the result of introgression with local female wild boars or the result of an indigenous domestication process (28). To address this issue, we sequenced 2 high-coverage, 7 medium-coverage, and 54 low-coverage ancient genomes spanning over 9,000 y. A neighbor-joining phylogenetic reconstruction of modern and ancient wild boars nuclear data reflects the distinct geographic partitioning of mtDNA data in western Eurasia (32). More specifically, distinct ancestries are present within ancient European and Near Eastern wild boars remains that predate domestication (Fig. 2*A* and *SI Appendix*, Fig. S10). An ADMIXTURE analysis of 38 wild boars nuclear genomes, including an ancient wild boars from Aşıklı Höyük (∼10,000 y BP, Turkey) reveals that modern wild individuals from Greece possess 33 to 38% Near Eastern nuclear ancestry, while those from Italy possess only 6 to 10% (Fig. 2*A*). The decreasing proportion of Near Eastern ancestry among wild boars from Greece to Italy most likely reflects admixture between wild populations from Anatolia into Greece and then into Italy (*SI Appendix*, Fig. S12). It is also possible, however, that a portion of the Anatolian ancestry found in Italian wild boars is the result of admixture from domestic pigs derived from the Near East into wild populations, instances of which have previously been shown to have occurred in northern Germany (33).

Additional ADMIXTURE analyses, including 111 genomes, clearly demonstrate that most modern domestic pigs (77 of 85) do not possess significant levels of Near Eastern ancestry (*SI Appendix*, Figs. S15 and S16). In fact, when modern European domestic pigs are treated as a single population, our haplotype-based analyses [GLOBETROTTER (34)] indicate that their overall Near Eastern ancestry is only ∼4% (*SI Appendix*), and most of this Near Eastern signal is derived from a few modern breeds from Italy, Hungary, and Spain that possessed 1.7 to 6.4% Near Eastern nuclear ancestry (Fig. 2*B*). Interestingly, the majority of these breeds occur in regions of Europe where modern wild boar possess, on average, higher levels of Near Eastern ancestry (6 to 33%; Fig. 2 *A* and *B*), and, as opposed to many other European populations, these breeds were not mixed with Chinese pigs during breed improvement programs during the 19th century (35, 36) (*SI Appendix*, Figs. S15 and S16). It is therefore likely that the limited Near Eastern ancestral component detected in these samples was acquired through gene flow with local wild boars (in Italy or the Balkans), and maintained as a result of a lack of admixture with introduced Chinese pigs.

We further assessed the degree of Near Eastern ancestry in archaeological pigs. Our ADMIXTURE analysis indicates that Bronze Age domestic pigs from western Iran (∼4,300 y BP: AA363) and Armenia (∼3,500 y BP: AA119) did not possess any European ancestry, and were exclusively derived from ancient Near Eastern wild boar (*SI Appendix*, Figs. S15 and S16). In Europe, 4 ancient high/medium-coverage domestic pigs did possess Near Eastern nuclear ancestry (Fig. 2*B*). Specifically, 2 early Neolithic samples from Herxheim, Germany (∼7,100 y BP: KD033, KD037) possessed ∼54% and ∼9% Near Eastern ancestry, respectively; a domestic pig from la Baume d’Oulen, France (∼7,100 y BP: AA288) possessed 15%; a Late Neolithic sample from Durrington Walls in Britain (∼4,500 y BP: VEM185) possessed ∼10%; and a 1,000-y-old Viking Age sample from the Faroe Islands (AA451) possessed only 5%. Of these, only the Herxheim sample (KD033), with ∼54% Near Eastern ancestry, possessed the Near Eastern mt-Y1 haplotype (Dataset S1), and also had substantially more Near Eastern ancestry than any of the ancient or modern European wild boar (Fig. 2*B*). This is supported by outgroup f3-statistics analysis, which indicates that KD033 shares more drift with Near Eastern wild boar than any other ancient or modern pig genome (*SI Appendix*, Fig. S17), as well as significant D-statistics of the form D (outgroup, Near Eastern wild boar; European wild boar: KD033) (Z << 3; *SI Appendix*, Fig. S18). These results indicate that European wild boars were being incorporated into domestic populations relatively soon after the latter were introduced from the Near East.

To obtain a more precise temporal and geographic resolution of the disappearance of Near Eastern genomic signatures in Europe, we performed additional analyses of 54 low-coverage ancient genomes (<1-fold) that possessed sufficient data (>5,000 SNPs covered from a panel of ∼12 million SNPs; *SI Appendix*) to be confidently projected onto a principal component analysis (PCA) alongside both modern and (high- and medium-coverage) ancient genomes. We analyzed these data together with those of Asian wild and domestic pigs. In this analysis, principal component 1 (PC1) separated European and Asian pigs, while PC2 separated Near Eastern and European pigs (*SI Appendix*, Fig. S14). After removing Asian pigs, PC1 separated modern European domestic pigs from all other samples, while PC2 separated European from Near Eastern pigs (Fig. 2*C*). The separation between European domestic pigs and all other samples on PC1 is most likely the result of admixture between Asian and European breeds following breed improvement programs in the 19th century (35, 36) (*SI Appendix*, Figs. S15 and S16).

The PCA revealed 2 groups of ancient European pigs (including 25 previously identified as domestic using a combination of morphometric and contextual data and 10 with unknown status) (Fig. 2*C*). The first group consisted of 8 domestic pigs that are closer to Near Eastern wild boars and ancient Near Eastern domestic pigs (Fig. 2*C*). In all, this group comprised Neolithic pigs from contexts dating from 7,650 to 6,100 y BP, including the following: Madzhari, Northern Macedonia (∼7,650 y BP: BLT022, BLT023); Herxheim, Germany (7,100 y BP: KD033, KD032); Măgura, Romania (7,100 y BP: BLT010); Pločnik, Serbia (∼6,650 y BP: AA212); Vinča Belo Brdo, Serbia (∼6,500 y BP: BLT014); and Căscioarele, Romania (∼6,000 y BP: AA072). Interestingly, 7 of these samples also possessed the Near Eastern mt-Y1 haplogroup (AA212 is unknown) (Dataset S1). We also identified 3 samples from Buran-Kaya, Crimea (∼7,000 y BP: AA380, AA480, AA483) that also cluster close to Near Eastern wild boars, although they each possess the mt-Y2 haplotype and so are thought to be local wild boars (Dataset S1).

The second group of ancient European samples was closer to wild and modern domestic pigs from Europe and included samples that are mostly younger in age than the first group. This second group consisted of 18 domestic samples from overall more recent archaeological sites dating from 7,100 to 900 y BP, including the following: Herxheim, Germany (7,100 y BP: KD037); Oulens, France (∼7,100 y BP: AA288); Bozdia, Poland (∼6,700 y BP: AA346; ∼900 y BP: AA343, AA341); Durrington Walls, England (∼4,500 y BP: VEM183, VEM184, VEM185); Utrecht, The Netherlands (∼2,300 y BP: KD025; ∼700 y BP: KD024); Basel, Switzerland (∼2,000 y BP: AA266); Coppergate, England (∼1,800 y BP: AA301); Undir Junkariusfløtti, Faroe Islands (∼1,000 y BP: AA451, AA411, AA414, AA418, AA440); and Ciechrz, Poland (∼900 y BP: AA139). This group also comprised 7 ancient samples that could not be identified as either wild or domestic, including the following: la Grotte du Taï, France (∼7,100 y BP: AA294); Santa Maria in Selva, Italy (Late Neolithic: AA629); and El Portalón, Spain (∼5,400 y BP: AA513; ∼4,500 y BP: AA507; ∼3,600 y BP: AA512, AA511;∼900 y BP: AA513). Lastly, 2 ancient wild boars, 1 from Birsmatten-Basisgrotte, Switzerland (∼7,700 y BP: AA241) and 1 from Siniarzewo, Poland (∼2,900 y BP: LG507) were also found to fall closer to modern European wild boars. All of these samples possessed a European mtDNA signature (Dataset S1).

Collectively, these results reveal a fluctuating temporal pattern of Near Eastern genomic ancestry in western Eurasian domestic pigs, and the general trend shows that the samples closer in time and space to the source of the first Near Eastern pigs possessed a greater proportion of Near Eastern ancestry. In mainland Europe, domestic pigs from Neolithic sites situated around the Styrmon (e.g., Northern Macedonia), Danube (e.g., Romania), and Rhin (e.g., Germany) river systems in Germany, Romania, Macedonia, and Serbia possessed substantially more Near Eastern ancestry than is present in European wild boar (Fig. 2 *B* and *C*). The timing of the first (∼8,000 y BP) and last (∼5,100 y BP) appearances of Near Eastern mtDNA signatures in continental Europe [apart from 4 Italian suids from AD 1800 (37)] is coincident with our nuclear data, indicating that <3,000 y after domestic pigs were introduced, their Near Eastern ancestry (at both mitochondrial and nuclear levels) had all but vanished. The hybrid nature of the high-coverage genome from the Neolithic Herxheim pig in Germany (7,100 y BP: KD033; Fig. 2*B*) indicates that this disappearance was most likely gradual, and was the result of gene flow from European wild boar into the introduced Near Eastern domestic pig populations.

### The Extent of Near Eastern Ancestry in Modern Domestic Pigs.

To assess the threshold above which we could confidently identify Near Eastern ancestry in our ancient data, we simulated genomes with predefined Near Eastern ancestry proportions and analyzed the data using ADMIXTURE (38). We then used a binomial distribution to compute the probability of successfully detecting Near Eastern ancestry in 8 of 85 genomes (reflecting our modern data) (Fig. 2*B* and *SI Appendix*, Fig. S19). For admixture values ≥5%, the probability of observing only 8 genomes with Near Eastern ancestry is <1% (*SI Appendix*, Fig. S19*A*). This indicates that ADMIXTURE should detect significantly more pigs with Near Eastern ancestry if the genome of every modern domestic pig possessed a Near Eastern component ≥5%. Additionally, our simulations indicate that the GLOBETROTTER (34) analysis can accurately detect 4% Near Eastern ancestry (*SI Appendix*, Fig. S19*B*), which is less than what is present in modern Italian and Balkan wild boar. If a degree of Near Eastern ancestry was essential for the maintenance of the domestic phenotype in Europe, we would therefore predict that the underlying causative variants are present in no more than ∼4% of the genome.

To further explore this possibility, we investigated whether regions of modern domestic pig genomes reported to be subjected to positive selection (26) were more closely related to either Near Eastern or European wild boar. To do so, we first phased modern and high-coverage ancient genome data using shapeit (39). For each positively selected region, we computed the nucleotide distance between every pair of domestic and wild haplotypes. For each domestic pig haplotype, we computed the normalized difference between the nucleotide distance of the closest European haplotype and the closest Near Eastern wild boar haplotype. We then plotted the mean and SD of this statistic for each sweep region (*SI Appendix*). Our results show that a large majority of domestic pig haplotypes within these sweep regions share a closer genetic affinity to European wild boars than to Near Eastern wild boar (271 of 298; *SI Appendix*, Fig. S20). In fact, we did not identify a single region that was closer to Near Eastern wild boars (*SI Appendix*, Fig. S20). This suggests that the majority of human-mediated selection that took place after the arrival of pigs in Europe most likely did not target haplotypes of Near Eastern origin. We could not, however, distinguish between European and Near Eastern ancestry in ∼10 sweep regions. Given the bias toward modern European wild boar haplotypes in our dataset, it is possible that our analysis did not possess sufficient power to identify Near Eastern ancestry in those ∼10 regions. Doing so will require additional sequencing of modern and ancient Near Eastern pigs.

### The Evolution and Dispersal of Black Coat Color.

To further assess the potential relevance of Near Eastern ancestry to the genetic and phenotypic makeup of early and modern domestic pigs, we investigated the Melanocortin 1 Receptor (*MC1R*) gene. This gene has been shown to harbor functional mutations (linked to the loss of camouflage coat color) that are highly correlated with domestic status (*SI Appendix*). Our analyses of previously published and novel modern and ancient *MC1R* sequence data (269 domestic pigs and 46 wild boar) demonstrate that a specific nonsynonymously derived mutation [D124N (40)], which is associated with black (or black and white spotted) coat color in western Eurasian domestic pigs, is almost absent in both modern and ancient wild boars from the Near East and Europe (1 of 92; *SI Appendix*, Fig. S8). The only wild boar that possessed 1 copy of the derived allele originated from a population in The Netherlands that is known to have recently interbred with domestic pigs (41). By characterizing this SNP in ancient domestic pigs (using NGS and PCR assays; Dataset S1), we identified 64 of 76 animals with at least 1 copy of the derived allele (the remaining 12 were homozygous for the wild type). Altogether, this suggests that while the ancestral allele at this locus cannot be used to unequivocally distinguish wild and domestic pigs, the derived allele is highly indicative of domestic status.

The earliest pigs that possessed the derived allele were found at Neolithic Ulucak Höyük in western Anatolia (∼8,650 y BP: AL1102; ∼8,250 y BP: Ulu48). The earliest European pigs that possess the derived allele are from Neolithic sites in Bulgaria (∼7,500 y BP: Cav6, Kov19), Romania (∼7,200 y BP: Uiv10), and Germany (∼7,100 y BP: KD033, KD037). Further phylogenetic analysis of the ∼100-kb region surrounding the *MC1R* gene indicated that 169 of 174 phased sequences, obtained from high-coverage modern and ancient domestic pigs that possessed the D124N allele, clustered in a monophyletic clade (*SI Appendix*, Fig. S9).

This result suggests that the D124N mutation found in Near Eastern and European pigs arose just once and was maintained, despite substantial gene flow with European wild boars. Interestingly, the nearest clade to this monophyletic cluster consisted of 2 haplotypes found in modern wild boar with European ancestry (The Netherlands) and Near Eastern ancestry (from Samos off the Anatolian west coast; *SI Appendix*, Fig. S9). This finding indicates that we do not possess the resolution to infer whether the D124N mutation (now fixed in many domestic breeds) first arose in the Near East or in Europe. Although we cannot definitively identify the geographic origin of the D124N mutation using phylogenetic analysis, the fact that it occurred in Anatolia before the arrival of domestic pigs into Europe, and that it likely arose only once, strongly suggests that this trait originated in Anatolia and was present in the first pigs that were transported into Europe.

## Conclusion

Our results indicate that the Anatolian wild boars domesticated ∼10,500 y ago were the ancestors of domestic pigs that were transported into Europe ∼8,500 y BP. By the late Neolithic (5,000 y BP), the Near Eastern genomic proportion of domestic pigs in Europe had dropped to <50%, and the Near Eastern fraction is now 0 to 4% in modern European domestic pigs. This near-complete genomic replacement and gradual disappearance of Near Eastern ancestry occurred over 3 millennia in continental Europe and was the result of hybridization between Near Eastern domestic pigs and European wild boars. This further implies that European domestic pigs did not originate from an independent domestication process, but rather from the continuous management of herds that were interbred (however intentionally) with local wild boar. In Mediterranean regions, including Sardinia (42), Corsica (42), Spain (43), Greece (44), and Roman Italy (45), swineherd management often allowed for pigs to seasonally range freely away from human settlements. Combined with other traditions such as pig transhumance (42), these practices likely offered the opportunity for reciprocal gene flow between wild boar and managed pigs, although, at least in some regions, a clear size difference persisted throughout. Our results suggest that these management strategies may have been practiced in Europe from the first introduction of pigs in the Neolithic.

The introgression from European wild boars eroded the proportion of Near Eastern ancestry in European pigs to levels that are potentially below our detection threshold. As predicted by a model in which European pigs were not independently domesticated, we found the existence of a genetic variant leading to black coat color (within the *MC1R* gene) that was transferred from the Near East into Europe by early farmers, where it resisted introgression from wild boar. This finding suggests that other regions of the genome that govern domestic phenotypes (e.g., smaller size) may also have retained their Near Eastern ancestry, but our analyses indicate that these regions make up no more than 4% of the genome. In fact, we show that the vast majority of human-mediated selection over the past 5,000 y focused instead on the genomic fraction derived from the European wild boars, and not on genomic variants that were selected by Near Eastern Neolithic farmers during the first 2,500 y of the domestication process.

Previous coalsecent simulations have shown that a genomic replacement of this magnitude, as a result of introgression from a local population into an invading population is expected, so long as the incoming population is relatively small and strong barriers to interbreeding do not exist (46). The degree to which the Near Eastern fraction of the earliest domestic pigs in Europe has been erased from the genome of modern European pigs is unprecedented. Despite the fact that introgression has also been shown to be common (47, 48) between local wild populations and translocated domestic animals [e.g., cattle (49), horses (50), dogs (51), chickens (52), goats (5)] and plant species [e.g., grapes (53), apples (54), maize (55, 56)], pigs are the only species that has experienced a genomic turnover so substantial that their original ancestry is barely detectable within modern populations. This suggests that pigs experienced a significantly smaller degree of reproductive isolation from their wild European counterparts than did other dispersing domesticates that encountered closely related wild species in the regions into which they were introduced [e.g., cattle (49), dogs (51)].

Overall, our results suggest that domestication narratives are not as straightforward as a simple dispersal of fully domesticated plants and animals out of the area of initial domestication. Instead, domestication is a protracted process, a significant proportion of which takes place through continual admixture and human-mediated selection. These perspectives underscore the temporally dynamic nature of the relationship between humans and domestic taxa, and our increasing ability to monitor this process by analyzing ancient genomic data within the context of metrical, isotopic, and other analyses.

## Supplementary Material

Supplementary File

Supplementary File

Supplementary File
