# Supplementary material for: Ancient pigs reveal a near-complete genomic turnover following their introduction to Europe
Source: Proc Natl Acad Sci U S A. 2019 Aug 12;116(35):17231–8. doi: 10.1073/pnas.1901169116 (PMC6717267; doi:10.1073/pnas.1901169116)
Supplement: Supplementary File [file pnas.1901169116.sapp.pdf]

Supplementary Information for

Ancient pig genomes reveal a near complete turnover following their introduction to Europe

Laurent A.F. Frantz, James Haile, Audrey T. Lin, Amelie Scheu, Christina Geörg, Norbert Benecke, Michelle Alexander,Anna Linderholm Victoria E. Mullin Kevin G. Daly, Vincent M. Battista, Max Price, Rose-Marie Arbogast, Benjamin Arbuckle, Adrian Bălăşescu, Ross Barnett, László Bartosiewicz, Gennady Baryshnikov, Clive Bonsall, Dušan Borić, Adina Boroneanţ, Jelena Bulatović, Canan Çakirlar, José-Miguel Carretero, John Chapman, Mike Church, Richard Crooijmans, Bea De Cupere, Cleia Detry, Vesna Dimitrijevic, Valentin Dumitrascu, Ceiridwen Edwards, Merih Erek, Aslı Erim-Özdoğan, Anton Ervynck, Domenico Fulgione, Mihai Gligor, Anders Götherström, Lionel Gourichon, Martien Groenen, Daniel Helmer, Hitomi Hongo, Liora K. Horwitz, Evan K. Irving-Pease, Ophélie Lebrasseur Joséphine Lesur, Caroline Malone, Ninna Manaseryan, Arkadiusz Marciniak, Holley Martlew, Marjan Mashkour, Roger Matthews, Giedre Matuzeviciute, Sepideh Maziar, Erik Meijaard, Tom McGovern, Hendrik-Jan Megens, Rebecca Miller, Azadeh Mohaseb, Jörg Orschiedt, D. Orton, Anastasia Papathanasiou, Mike Parker Pearson, Ron Pinhasi, Darko Radmanović, François-Xavier Ricaut, Mike Richards, Richard Sabin, Lucia Sarti, Wolfram Schier, Shiva Sheikhi, Elisabeth Stephan, John R. Stewart, Simon Stoddart, Antonio Tagliacozzo, Nenad Tasić, Katerina Trantalidou, Anne Tresset, Cristina Valdiosera, Youri van den Hurk, Sophie Van Poucke, Jean-Denis Vigne, Alexander Yanevich, Andrea Zeeb-Lanz, M. Thomas P. Gilbert Jörg Schibler, Melinda Zeder, Joris Peters Thomas Cucchi, Daniel G. Bradley, Keith Dobney, Joachim Burger, Allowen Evin, Linus Girdland-Flink, and Greger Larson

Corresponding authors: Laurent A. F. Frantz – laurent.frantz@qmul.ac.uk ; Greger Larson – greger.larson@arch.ox.ac.uk

This PDF file includes:

Materials and Methods
Figs. S1 to S22

Other Supporting Online Material for this manuscript includes the following:

Tables S1, and S2 (Excel)

www.pnas.org/cgi/doi/10.1073/pnas.1901169116

Archaeological Site Descriptions **3**

Ancient DNA **53**

Data generation - mtDNA - Durham *54*

Data generation - MC1R/mtDNA *55*

Data generation - Nuclear and mtDNA capture - Oxford *57*

Data generation - Nuclear - Stockholm *57*

Data Processing **58**

Sexing *58*

MapDamage *58*

Publicly available sequences *59*

High coverage ancient genomes *59*

Ascertainment and ancient genome quality *59*

Low coverage data 60

mtDNA analyses **60**

Filtering 60

Building a database for haplogroup assignment 60

Phylogenetic analysis and reference sequences 60

A method for rapid haplogroup assignment 61

Low coverage data 62

mtDNA lineage geographic distribution (Figure 1) 62

Samples excluded from Figure 1 62

Nuclear DNA analyses **64**

MC1R and domestic/wild status 64

Ancestry analysis 65

Neighbour Joining tree 65

Modelling population history of wild boars *66*

Low-coverage data - testing PCA *66*

PCA *67*

ADMIXTURE *67*

F3 & D-statistics *68*

Phasing, Chromopainter and GLOBETROTTER *69*

ADMIXTURE simulations *70*

GLOBETROTTER simulations *70*

Sweep ancestry *71*

X chromosome *71*

Supplementary Tables **72**

Supplementary Figures **73**

Archaeological Site Descriptions

Abri Fuchskirche I near Allendorf (Thuringia), Germany
Contact: Norbert Benecke
Abri Fuchskirche I is a rockshelter site near Allendorf in Thuringia, Germany. Occupation layers are of the late glacial period (Bølling – Allerød); there are four 14C dates on bones of elk, wild horse, and aurochs (1). One wild boar specimen (GL115) was analyzed.

Abu Ghosh, Israel
Contact: Liora Kolska Horwitz
Abu Ghosh is situated in the Judean Hills, ca. 12 km west of the city of Jerusalem. Several excavations at the site since the 1950s have documented remains of a mid-Pre-Pottery Neolithic B (PPNB; 10,100-9,250 cal BP) village, containing rectangular houses with plaster floors, installations, intra-mural human burials and a rich assemblage of lithic artifacts, groundstone vessels and fauna (2); (3) and papers therein). The single sample (AA042) analyzed in this study from this site is wild boar.

Acemhöyük, Turkey
Contact: Benjamin Arbuckle
Acemhöyük is a large mound located at an elevation of 950 m on the alluvial fan of the Melendiz river near the Tüz Gölü (Salt Lake) in Central Turkey. The mound is approximately 800 x 600 m in dimension rising 20 m above the surrounding Yesilova plain and represents the remains of a large, fortified Bronze Age urban center. The site has been excavated since 1962 by Dr. N. Özgüç, Ankara University in association with the Turkish Historical Society and the General Directorate of Antiquities and Museums and by Prof. Dr. A. Öztan of the same institution since 1989 (4); (5). Acemhöyük consists of 12 levels with deposits representing Chalcolithic to Medieval occupations.

Four *Sus* samples (all representing small sized and therefore probably domestic pigs) were submitted for ancient DNA analysis. Two of these samples (AA525 and AA526) derive from deposits stratigraphically assigned to level XI (associated radiocarbon date c. 4,000 cal BP), representing the Early Bronze Age; the remaining two specimens (AA324 and AA325) derive from level III representing the Assyrian Colony period of the Middle Bronze Age (3,700s cal BP).

The animal economy at MBA Acemhöyük is dominated by domestic sheep, goats and cattle. Domestic pigs are present in MBA levels II and III where they represent c. 10-20% of the identified remains depending on context (6). The Acemhöyük faunal assemblage is curated on site at the Acemhöyük dighouse, Yesilova, Aksaray, Turkey. Specimens were exported with the permission of the Konya Museum and the Turkish Ministry of Culture and Tourism.

Ajvide, Sweden
Contact: Göran Burenhult
The Stone-Age settlement and burial ground at Ajvide in Eksta Parish is situated on the south-west coast of the island of Gotland in the Baltic Sea. During the period of use, c. 5,000-4,300 cal BP, the site was located at a lagoon by the sea. Today, the site is situated on a slope between 12 and 17 m above the sea level. It belongs to the Pitted-Ware Culture in the late Middle-Neolithic period of the Swedish Stone Age.

The site covers an area of c. 200,000 m^2, and has been partially excavated 1983-2009. The excavated area is c. 3,000 m^2, and so far 85 skeleton graves have been documented. Thanks to the limestone-rich soil, the skeletons, as well as the animal bones, are exceptionally well preserved and both osteological and DNA analyses have been successfully performed. The settlement predates the burial ground, and was occupied between c. 5,000-4,750 cal BP. The earliest graves can be dated to c. 4,750 cal BP, and the burial ground was used until c. 4,300 cal BP (7); (8); (9). The samples from the site include AA289, AA290, AA291, AA292, and AA293.

Alba Iulia-Lumea Nouă, Romania
Contact: Mihai Gligor, John Chapman
The Neolithic and Eneolithic settlement at Alba Iulia-Lumea Nouă was discovered by chance in the early 1940s, when the area was subject to public utility works. The site is located on the second terrace of the Mureş river in the north-eastern part of the city of Alba Iulia, Transylvania (Romania), at 240-246 m altitude, and covers some 40 ha. In the past 15 years, continuing residential development of the city has resulted in several rescue excavations, which have provided evidence of habitation by Vinča and Lumea Nouă cultures (Middle Neolithic), Foeni and Petreşti cultures (Early and Middle Eneolithic). The site is considered one of the most important settlements in Transylvania on the basis of the quality of painted pottery and funerary complexes with commingled human remains. The six animal bone samples analyzed in this study (AA384, AA385, AA386, AA387, AA388, and AA389) belongs to the Early Eneolithic Foeni period (5,770-5,550 BP) (10); (11); (12); (13); (14).

Albertfalva Roman vicus (Budapest), Hungary
Contact: Keith Dobney, László Bartosiewicz
The Albertfalva Roman vicus was located in Pannonia province on the right bank of the Danube in the southern outskirts of present-day Budapest, Hungary (15). This section of the river once formed the eastern limes of the Roman Empire. Approximately one quarter (25.9%) of the 1,888 identifiable bones (NISP) previously published by Bökönyi (16) originated from domestic pig. The material was dominated by the remains of domesticates, including a relatively high proportion of horse, explained by the presence of Roman cavalry units nearby. The two samples from this site (AA408 and AA409) date to the 2/3rd century AD.

Alepotrypa Cave, Greece
Contact: Anastasia Papathanasiou, Mike Richards
Alepotrypa Cave is located at Diros Bay, Lakonia, Greece. The cave is about 300 m long and it is situated 50 m from the present Mediterranean shoreline, in an arid and rocky limestone environment. It has been excavated since 1970 and it is dated from 8,000 to 5,200 BP, from the Early to the Final Neolithic. Artefacts include a variety of pottery, lithic tools, grindstones, personal decoration items, figurines and food remains consisting of cultivated cereal, legume, and fruit remains, a large number of animal bones from domesticated species, and to a lesser degree of wild plant and animal resources, fish and shells. Stable isotope analysis suggests a primarily agricultural diet with emphasis on plant resources. The cave also yielded a large human skeletal assemblage of primary and secondary disposals and evidence of rich ritualistic expression, including massive concentrations of deliberately broken pots

possibly associated with mortuary practices (17); (18). One specimen (GL1081) analyzed dates to the Middle Neolithic.

Altenburg-Rheinau, Germany
Contact: Joris Peters
Altenburg-Rheinau is a La Tène culture site founded in the middle of the 4th century BP its abandonment likely coincided with the Roman conquest of the northern alpine forelands 2,015 BP. Site occupation therefore essentially coincided with the La Tène D cultural period. Excavations in the 1970s produced a large faunal assemblage comprising more than 10,000 remains of pigs, the second most important taxon in terms of numbers after cattle (19). Ten specimens from this site (LG161, LG162, LG577, LG578, LG579, LG580, LG582, LG583, LG584, and LG585) date to the Iron Age.

Arbon Bleiche 3, Switzerland
Contact: Jörg Schibler
Arbon Bleiche 3 is a Neolithic lakeshore site in Switzerland situated at the southern shore of Lake Constance. The site is known since more than 130 years but was excavated for the first time in 1983 (46 m^2) but to a larger extent (1,050 m^2) between 1993 and 1995 (20). Drilling exploration revealed that at least one third and at most half of the Neolithic village was excavated. As typical for wetland sites Arbon Bleiche 3 is dated by dendrochronology. The settlement period spans in total 15 years, most probably between 5,384 BP and 5,370 BP. During the excavation (1993 and 1995) 71,472 animal bones were excavated, and 32,261 could be identified to the species level (21). 23 specimens (AA181, AA182, AA183, AA184, AA185, AA186, AA187, AA189, AA191, AA192, AA193, AA195, AA255, AA256, AA257, AA258, AA259, AA260, AA261, AA262, AA263, AA264, AA265) from this site date from the Late Neolithic to the Bronze Age. The bones were provided by Amt für Archäologie Thurgau for this study.

Aşağı Pınar, Turkey
Contact: Norbert Benecke
Aşağı Pınar (lower well) is the name of Middle to Late Neolithic *tell* (8,200-6,300 cal BP) in the eastern part of Turkish Thrace close to Kırklareli. Its location alone already makes it predestined to mirror the relationships between the two areas, and the archaeological finds indeed resemble both, Anatolian and Balkan Cultures (22). The domestic faunal assemblage reveals a quite stable relative composition throughout the settlement history with a slight dominance of sheep and goat, followed by cattle and only few pigs (up to 7%) (23). The 15 samples analyzed here represent both the Middle and Late Neolithic horizons and date to 7,500-7,000 cal BP (AP23, AP24, AP25, AP26, AP27, AP28, AP29, AP30, AP31, AP32, AP33, AP34, AP35, AP36, AP37). One domestic sample (AP26) turned out to come from an only around 2,500 year old Hellenistic intrusion (KIA-42161: 2415 ± 30 BP). One wild boar and eight domestic pigs were successfully analyzed for a partial mtDNA fragment.

Aşıklı Höyük, Turkey
Contact: Mihriban Özbaşaran, Joris Peters
Aceramic Neolithic Aşıklı Höyük is a 16 m high settlement mound located next to the Melendiz River in east Central Anatolia, the volcanic region named historically as Cappadocia. Site habitation started prior to 10,300 BP and came to an end after 9,500

BP. Stratigraphic analysis allows defining five occupation levels with multiple building phases, distinguished by their architecture and material culture. This archaeological sequence is pivotal to our understanding of the process of Neolithization in Central Anatolia. Of particular interest from a faunal viewpoint is the discovery of extensive dung layers at the site, implying that people held animals captive in the village from the earliest habitation onwards. Zooarchaeological analyses point to intensifying management of sheep and goat with time, alongside fishing, fowling, and hunting: Besides aurochs and red deer preferred game also included wild boar (24); (25); (26). 14 samples (AL643, AL644, AL645, AL646, AL647, AL706, AL708, AL712, AL714, AL718, AL720, AL724, AL725, AL726) date to the Aceramic Neolithic.

Atij, Syria
Contact: Melinda Zeder
Atij dates to c. 4,700-4,400 BP, and is located on the middle Khabur River in northeastern Syria (27). The number of large silos and other storage facilities encountered here suggests that that may have served as entrepots involved in the coordination and transshipment of agro-pastoral products (28); (29). One suid sample (AA051) dates to the Ninevite V period.

Augusta Raurica, Switzerland
Contact: Jörg Schibler
Augusta Raurica is a roman town about 12 km east of Basel, Switzerland. The town was founded in the early years of the 1st century AD and was settled as a civil town until the 4th century AD. Animal bones are systematically identified during the 1950s and 1960s by Elisabeth Schmid. This archaeozoological data are published in Schibler and Furger (30), but other archaeozoological data have been published elsewhere (e.g. (31); (32)). Two samples (AA268 and AA269) dates to the Roman period. The bones were provided by Museum Augusta Raurica for this study.

Bademağacı Höyük, Turkey
Contact: Bea De Cupere
Bademağacı Höyük is situated in the south of the Lake District (SW Turkey), about 50 km north of Antalya. This oval shaped mound lies at an altitude of 780 m a.s.l. on a small plain surrounded on all sides by mountains. The Early Bronze Age (EBA II) is evidenced by three architectural levels and a stone paved terrace and covers an Early Neolithic occupation (9th millennium BP) (33). 5 samples (AA305, AA306, AA307, AA309, AA310) date to the Neolithic.

Balanbash (Krasny Yar), Russia
Contact: Gennady Baryshnikov
Balanbash settlement is situated at Belaja River (Volga River basin) near Krasny Yar village, close to Sterlitamak city, in Russia. It is dated by the middle part of Bronze Age (34). The bone material of *Sus scrofa domesticus* (ZIN 18451) came from K. Salnikov excavation in 1934. It comprises 43 bone fragments from 7 adult specimens (calculated by G. Baryshnikov). One sample (LG236) was analyzed that dates to the Iron Age.

Basel-Gasfabrik, Switzerland
Contact: Jörg Schibler

Basel-Gasfabrik is an urban centre site with two cemeteries of the late Iron Age period. The site is situated at the southern Rhine shore in Basel. Most of the archaeological material are dated between 2,200-2,150-2,080 BP and belong to the La Tène periods C2 and D1. Several excavations between 1911 and today took place. The urban site was unfortified and covered an area of about 15 ha that offered space for several 100 people (35). Two domestic samples (AA266 and AA267) analyzed date to the late Iron Age. The bones were provided by Archäologische Bodenforschung Basel-Stadt for this study.

Bercy, France
Contact: Anne Tresset
Bercy (Paris, France) is a site located on the right bank of the Seine River, downstream from the confluence with the Marne River. The site corresponds to a hamlet set on the riverbank. The main period of occupation is dated to ca. 6,000 cal BP and belongs to the Chasséen septentrional culture (regional Middle Neolithic). The faunal assemblage is dominated by domesticates. The suid bones included in the present study came from this cultural horizon and accounted for ca. 10-20% of the identified mammal remains depending on the layer considered (36). 14 samples (AA492, AA493, AA494, AA495, AA496, AA498, AA499, AA500, AA501, AA502, AA503, AA504, AA505, AA506) date to the French middle Neolithic.

Birsmatten-Basisgrotte, Switzerland
Contact: Jörg Schibler
Birsmatten-Basisgrotte is a Mesolithic rock shelter in the north-west of Switzerland about 18 km south of Basel in the Village Nenzlingen. The site was excavated by Carl Lüdin between 1940 and 1945 and 1955-56 under the direction of Hans-Georg Bandi. The excavated material was divided in five horizons (I to V). All five horizons were typologically dated to the Mesolithic. 14C samples were analyzed in the early 1960s and published by Gfeller (37). The calibrated (Oxcal) dates show for the horizons V to III Mesolithic values of the 9th and 10th millennium cal BP, whereas for the horizon II Mesolithic and Neolithic and for horizon I only Neolithic dates are found. Elisabeth Schmid (38) has identified the animal bones and mentioned only for the horizon I the possibility that domestic pig could be present due to the smaller sizes of the bones. Eleven samples (AA241, AA242, AA243, AA245, AA263, AA264, AA265, AA267, AA268, AA269, AA270) date from the Mesolithic to the Neolithic.The bones were provided by Archäologie Baselland and Naturhistorisches Museum Bern for this study.

Blätterhöhle Hagen, Germany
Contact: Jörg Orschiedt
The Blätterhöhle site is located in Hagen in North Rhine-Westphalia, Germany. Archaeological excavations in the cave and rock shelter site started in 2006 and research is ongoing. The rock shelter contains a stratigraphy from the late Mesolithic to the late Palaeolithic. Finds include stone artefacts, faunal and human remains as well as several hearths with activity zones. In the cave, the stratigraphy shows two main layers. The upper layer contains human remains from the late Neolithic (according to local chronology), and the lower layer early Mesolithic human remains. Faunal remains are present in all layers. Radiocarbon dates are between 11,200 and 10,600 cal BP for the early Mesolithic, and 5,900 and 5,000 cal BP for the Late Neolithic. Neolithic collective burials in caves and rock shelters are known for the 6th

millennium BP in other areas (Belgium, British Isles, Ireland) but not for the Westphalian mid-range mountain area. The Blätterhöhle provides the first clue that this burial practice. Although from bioturbated sediments and without anatomical context the very well preserved human remains, provide a rich source of information.

Ancient DNA sampling showed results both for Mesolithic and Neolithic remains. The results suggest that the Mesolithic population of the Blätterhöhle represents a typical hunter-gatherer population (mitochondrial haplogroups U5/U4), whereas the Neolithic population seems to be an admixture of hunter-gatherer (haplogroup U5) and farmer lineages (haplogroup H and others). Additionally the analysis of stable Isotopes (13C/15N/34S) was carried out in order to reconstruct long-term diets. The results show three distinct clusters with significant differences. Terrestrial diet was evident for both the Mesolithic and a Neolithic group, but the diet of one Neolithic group was based on the consumption of freshwater fish. This group consists of people exclusively with haplogroup U5. This result demonstrates a "non-Neolithic" way of life in the 6th millennium BP (39); (40); (41). Three specimens (Hag1, Hag2, Hag3) date to the the Mesolithic.

Bodzia 1, Poland
Contact: Arkadiusz Marciniak
The site in Kujavia, in the Polish lowlands was discovered in 2000 and excavated in the 2000s as a part of large rescue excavation project preceding the construction the A1 motorway. An area of nearly 2 ha was exposed, and 7 cultural levels spanning from the Neolithic to the early modern period were distinguished. The most distinct were LBK and Late Lengyel settlements represented by longhouses and more than 200 different character features. A large cemetery with elaborated chamber-like graves and rich grave goods dated back to the 10th-11th centuries AD have no parallels in Europe. Out of Four samples were dated to LBK (AA154, AA336, AA337, AA339), four to Neolithic (AA330, AA334, AA335, AA338), one to Bronze Age (AA331) and ten to Early Medieval period (AA149, AA155, AA332, AA333, AA340, AA341, AA342, AA343, AA344, AA345) (42); (43). LG750 dates to the late 9th millennium BP.

Boulancourt, France
Contact: Adrian Bălășescu, Jean Denis Vigne
The site of "Le Chatelet" (Seine-et-Marne, France), located on the western edge of the Gâtinais Beauceron plateau, is a fortified hill settlement (44) occupied at the end of the Late Bronze Age IIIb. It has produced more than 33,000 faunal remains of which almost 8,000 have been determined. The choice of particular products of stock-breeding for quality meat consumption and the diversity of hunting, fishing and trapping suggest a privileged social milieu, and fits the aristocratic nature of the site. The analysis of the fauna reveals a strong predominance of the remains of domestic species, within which the pig represents more than 60% in the number of remains (45). Five pig mandibles (AA296, AA297, AA298, AA299, AA300) were analyzed in this study.

Bozejewice 28, Poland
Contact: Arkadiusz Marciniak
The site was discovered in the 1990s during the rescue excavation project preceding the construction of the Yamal pipeline from Siberia to western Europe. Remains of

three cultures were unearthed: (a) Late Lengyel trapezoidal longhouse, (b) Bell Beaker burial, and (c) Lusatian culture settlement (46). Four samples (LG761, LG762, LG763, LG764) date to the Neolithic.

Bruchsal-Aue, Germany
Contact: Elisabeth Stephan
This Michelsberg enclosure located northeast of Karlsruhe in southwest Germany was excavated between 1987 and 1993. The site consisted of three ditches with numerous gateways, partially encircling a crest of a hill which was nearly devoid of finds, probably because of erosional processes. The ditch fillings were abundant with ceramics and animal bones and a segment of the outer ditch contained several aurochs skulls, deposited vessels and burials at the bottom and alongside the ditch, indicating potentially an additional function of parts of this site as sanctuary. Based on typological and chronological analysis of the pottery and radiocarbon measurements of bones the earthwork is dated from c. 6,250-5,650 BP. Nine specimens of domestic pigs were analyzed (LG602, LG603, LG604, LG606, LG608, LG609, LG611, LG618, LG619).

Bundsø, Denmark
Contact: Kim Aaris-Sørensen, Peter Rowley-Conwy
Bundsø is a Neolithic settlement situated on the Island of Als. Bundsø is a causewayed camp defined as a site demarcated by one or more parallel rows of long or short ditches interrupted by causeways and flanked on the inner side by a palisade. A feature shared by most causewayed camps is their position on projecting landscape formations. Bundsø is found on a flat, low-lying ground on the Flintholm peninsula, protruding into a now-drained fjord. A rich deposit of settlement material has been excavated on the bank of the peninsula, mainly dating from MN AIII, with a smaller part from MN AV. The excavation in 1980–81 located a double row of ditches running across the neck of the peninsula, covered by layers with settlement material from the MN AIII. Human skulls have been found among the settlement remains on various occasions, for example found outside the ditches, embedded in waste from later settlements (47).

Pigs excavated from Bundsø come from the early part of the Middle Neolithic, specifically Funnel Beaker Culture (5,300-4,500 BP), and forms the type assemblage of the MN A III period. The site comprised a 200 x 100 m area of cultural debris; no house plans discerned although burnt clay daub suggests they were present. Many finds of cultivated cereals and domestic animal bones attest to a predominantly agricultural economy. The earliest domestic pig samples found from southern Scandinavia date to the Middle Neolithic (48). One domestic pig specimen (AA015) was analyzed.

Bunnik Odijk, Utrecht, Netherlands
Contact: Youri van der Hurk
In 2000, near Bunnik Odijk, Utrecht the remains of a settlement were discovered. Several postholes and ditches were found, including those of a what appears to have been a Middle Iron Age granary. Pottery remains suggest that the site was occupied during the Middle Iron Age (2,500-2,250 BP) and during the Late Roman/Early Medieval period (4-7th century AD). The sample taken from this site (KD025) dates to the Middle Iron Age (49).

Buran-Kaya IV, Ukraine
Contact: Alexandr Yanevich
Buran-Kaya IV is located in the South Ukraine in the mountainous part of the Crimean peninsula at a distance 30 km northeast from Simferopol. The rock shelter site is situated 8 m up the bank of a small stream at 326 m in altitude above sea level. Structures of the habitation of the Neolithic layers of the site are represented only by ash and charcoal lenses from season fireplaces. Late Neolithic layers have AMS-dates 7,800-7,300 BP. The status of the pigs from Buran-Kaya IV is unclear. Archaeological context of the Crimean Neolithic allows to assume, that there were wild pigs, but the presence of the domesticated individuals cannot be excluded. 29 samples (AA372, AA373, AA375, AA376, AA377, AA378, AA380, AA381, AA382, AA476, AA477, AA478, AA479, AA480, AA481, AA482, AA483, AA484, AA485, AA486, AA487, AA488, AA489, AA490) were analyzed.

The Y2 haplogroup pigs have Balkanian origin: they came to Crimea from Balkans in the Final Pleistocene. Status of the pigs from Buran-Kaya IV and Crimean Neolithic in general is unclear. The lack of the sedentism of the Neolithic population and evidence of the developed hunters-gatherers economy allows to assume, that there were wild pigs.

Buxheim, Germany
Contact: Joris Peters
Buxheim, a Late Neolithic settlement of the Münchshöfen culture, was inhabited between ca. 6,500-6,150 BP. Excavations between 1996 and 1998 revealed a diverse faunal assemblage dominated by domestic animals but with a considerable contribution of game species (c. 30%), including red deer, wild boar, roe deer, brown bear and beaver. Pigs are the second most important domestic species. Faunal analysis has been carried out by C. Kitagawa and J. Peters, but the results are not published yet. Two domestic samples (LG177 and LG558) date to the Late Neolithic.

Cabeço da Amoreira, Portugal
Contact: Cleia Detry
Cabeço da Amoreira is part of the same complex of the Muge shell middens, and is located in the left bank of the Muge river, in front of C. Arruda. It was a smaller site only excavated since the 1930s, and continues to be excavated today. Occupied by the last hunter-gatherers, the only domestic species is the dog represented by scarce fragments of bones (50); the suids (AA350, AA351, AA354) have been all classified has wild boar (51), and date to the Mesolithic.

Cabeço da Arruda, Portugal
Contact: Cleia Detry
Cabeço da Arruda is an archaeological site part of a complex of Mesolithic shell middens located in the Tagus estuary (Portugal), that were occupied from 8,200 cal BP to 7,600 cal BP. The sites were produced by the last hunter-gatherers in the Iberian Peninsula which had no domestic species except for dogs (51). Therefore the suids from this site (AA352 and AA353) can clearly be classified as wild boar .

Çadır Höyük, Turkey
Contact: Benjamin Arbuckle

Çadır Höyük is a 32 m high mound located in the Kanak Su basin situated in the Yozgat Province of north central Turkey. The modestly sized 240 x 185 m mound boasts an occupational history spanning 6,000 years (ca. 7,200 BP to 13th century AD). This sequence has been explored since 1993 by the Çadır Höyük Archaeological Project led by Ron Gorny and more recently Greg MacMahon and Sharon Steadman (52); (53).

Four *Sus* specimens from Çadır Höyük were submitted for ancient DNA analysis. Three of these specimens (AL1105, AL1106, AL1108) derive from Late Chalcolithic deposits dated to the late 6th millennium BP. One specimen (AL1107) was recovered from a locus assigned to the Iron Age. Pigs represent 16-30% of the faunal assemblages from multiple phases of the Late Chalcolithic and 5% of the mammalian remains from the Iron Age at Çadır. The vast majority of pig remains are small-bodied, domestic animals (54). The Çadır Höyük faunal assemblage is curated on site at the Çadır Höyük dighouse, Peyniryemez, Sorgun, Turkey. Specimens were exported with the permission of the Yozgat Museum and the Turkish Ministry of Culture and Tourism.

Çamlıbel Tarlası, Turkey
Contact: László Bartosiewicz
The Late Chalcolithic (5,590–5,470 cal BP) settlement of Çamlıbel Tarlası was located on the Anatolian plateau near Boğazkale, 200 km east of Ankara. The small rural settlement, built on rolling hills consisted of stone buildings. Evidence of early copper smelting was identified at the site (55). Pig remains made up one third (33.6%) of the bone material consisting of 864 identifiable specimens originating overwhelmingly from domesticates (56). Eight specimens were analyzed: BLT011, BLT012, BLT015, BLT016, BLT017, BLT018, BLT019, and BLT020.

Cârlomanesti - La Arman, Romania
Contact: Adrian Bălășescu
The site at Cârlomanesti - *La Arman* (settlement and necropolis) is located on a small plateau on the valleys of Buzău River and its tributary, the Nişcov River, on the southwestern part of the village of Cârlomăneşti (Buzău county). Both the settlement and the necropolis were dated to the Middle Bronze Age (Monteoru culture), with features attributed to the settlement at times overlapping directly the burials. The remains of an Early Medieval settlement (6th-7th centuries AD) overlap and cut into the Middle Bronze Age occupations (57). Middle Bronze Age faunal remains consist predominantly of domesticate bones, with only very few wild species represented. The present sample (AA078) from a pig mandible originates from the Monteoru feature labelled C4 (58).

Casa Solima (Troina), Italy
Contact: Simon Stoddart
Casa Solima was a hut preserved under colluvium on a south facing terrace at 636 m a.s.l., sheltered to the north by a flysch outcrop, in the southern approaches to the Nebrodi mountains in northeastern Sicily, dating to the Serraferlicchio phase of the Chalcolithic (c. 5,500 BP). As such, in a tradition of research that concentrates on the funerary, it represents very rare settlement evidence containing highly fragmented food refuse where sheep (62%) and cattle (16%) were dominant, but pig (11%) was also present including samples contributing to this paper. Amongst the cereals einkorn

and hulled barley were the most frequent, as well as fruits and oil rich seeds. The settlement was probably more extensive, but only preserved in this one distinct geomorphological location (59). Four samples from this site were analyzed: AA270, AA271, AA272, AA273.

Căscioarele A, Romania
Contact: Adrian Bălăşescu
Ostrovel point is *tell* with a 103 x 57 m diameter with 5 m of archaeological deposits, located on an island on Cătălui Lake, Călăraşi County. The prehistoric settlement (60) is among the few intensely studied at that time, giving us a glimpse on the evolution of the fauna and the animal economy. The lowermost level of the *tell* is the Spanţov stage of the Boian culture, followed by massive debris of a Gumelniţa A2 settlement. The next level, entirely excavated, is dated in the Gumelniţa B1 phase (61). The pig bones originate in the Gumelniţa A2 (6,500-6,200 BP) level (5 remains from domestic pig - AA067, AA069, AA071, AA072, AA073; and 2 from wild boar - AA070, GL566).

Čavdar, Bulgaria
Contact: Norbert Benecke
The early Neolithic settlement of Čavdar is located east of the Bulgarian capital of Sofia in the southern part of a narrow basin between Sredna Gora and the Balkan mountains (62). The faunal assemblage of Čavdar is characterised by 90% of domesticated animals, slightly dominated by sheep and goat. Most of the adult pigs represent wild boar (63). The six *Sus* remains analyzed in this study (four domestic, two wild, one probably wild - Cav1, Cav2, Cav3, Cav4, Cav5, Cav6) come from Early Neolithic phases (Karanovo I/Kremikovci I) and date to approximately 8,000-8,500 cal BP.

Cave of Cyclops (Youra), Greece
Contact: Katerina Trantalidou
The rocky island of Youra, whose total surface area is 11 km², lies at the edge of two of the most fish productive areas in the Aegean, a crucial parameter for a period of increasing exploitation of marine resources as a response to rising sea-levels. Excavations (1992-1996) at the Cave of Cyclops (37°27′N, 24°10′E) attested human presence, mainly, from the Mesolithic to the Final Neolithic period (ca. 10,600-5,500 BP) as well as during the first centuries AD. The buck of organic remains (in terms of density per volume) and the bone implements offer a rare glimpse into the subsistence patterns of Mesolithic - EN fishing/bird hunting communities. Engaging in fishing suggests also knowledge of seafaring, while the material culture (e.g. the stone artefacts) evidenced the existence of exchanging networks on both sides of the Aegean Sea and the Islands.

In the lowest deposits of the Cave, suids were the only mammalian vertebrates to be found. However, from Upper Mesolithic onward, they did not exceed the 1% among Ungulates (in terms of NISP). Suids, which could have circulated freely on the island could have provided an alternative source of protein when fish and birds were not accessible.

The bone sample (LG481), mainly that of the Lower Mesolithic, evidenced the following characteristics and interaction with humans: 1) their diet relied on vegetal

resources; 2) the comparison of the height from all periods showed that swine ranged from medium-sized (LM) to small sized (UM) -similar morphometrics have been attested for the suids of Maroulas on Kythnos, Cyclades (10,930-10,410 cal BP); 3) there were twice as many females as there were male animals; 4) the slaughter pattern affected the younger animals (97% were killed up to two years old); 5) the common effects of domestication, such as the shortening of skull, can be seen from the EN period onward. Two specimens (LG481, LG482) produced Y2 and Y1 haplotypes, and have ages corresponding to the Upper Mesolithic and Early Neolithic cultural periods (64); (65); (66); (67); (68); (69).

The site was excavated by Professor Ad. Sampson, University of the Aegean.

Çayönü, Turkey
Contact: Hitomi Hongo
Çayönü Tepesi is located on one of the small tributaries of the Tigris at an elevation of about 800 m at the foot of the Taurus Mountains in the plain of Ergani, 60 km north of Diyarbakır in southeastern Turkey. The occupation of the site spans about 3,000 years (c. 12,000-8,500 cal BP), from Late PPNA into the early Pottery Neolithic.
The Prepottery Neolithic period at Çayönü is divided into six subphases, with each period named for characteristic architectural types. The Round Building subphase (ca. 10,200-9,400 uncal BP) and the early Grill Building subphase (9,400-9,200 uncal BP) are contemporary with the Prepottery Neolithic A (PPNA) period. The late Grill Subphase (9,200-9,100? uncal BP) and the Channelled Building Subphase (9,100-9,000 uncal. BP) belong to the Early PPNB, followed by the Cobble Paved Building Subphase (9,000-8,600? Uncal BP, Middle PPNB), the Cell Building Subphase (8,600-8,300 uncal BP, Late PPNB) and the Large Room Building Subphase (8,200-8,000? uncal BP. The Prepottery sequence is immediately followed by early Pottery Neolithic Phase, but only a small area of this phase has been excavated. The Pottery Neolithic layer is partially disturbed by the Early Bronze Age graves and Early Iron Age buildings (70); (71); (72).

Sample AA514 comes from a mixed context that mainly belongs to Cell/Large Room subphase, but with some pottery was also found. Sample AA515 came from a context of late Cell Subphase. Sample LG459 was also analyzed, and dates to 7,000-6,000 BC. Although these samples came from a feature that has been described as the late/final PPNB/PN mixed (thus the date of c.7500-6000 cal BC). However, Çayönü's PN contexts is often mixed with Iron Age material, therefore it cannot be excluded that this sample possibly belonged to the Iron Age unless it is directly dated.

Cazis-Petrushügel, Switzerland
Contact: Jörg Schibler
Cazis-Petrushügel is a Neolithic dryland site that is dated to the later Horgen Culture (4,800 BP-4,900 BP) by 14C-dating. The site is situated in the alpine region of the canton Grison 3 km northeast of the village Thusis 740 m over sea level. The site was excavated 1938-39 (Walo Burkart) and 1981-82 (Margarita Primas). A small sample of animal bones were identified and published in (73). The specimen analyzed AA074.

Cencelle, Italy

Contact: Claudia Minniti
Excavations in area III of the town of Leopoli-Cencelle (ancient Centumcellae) was carried out in the 1990s by the Ėcole Françoise of Rome under the direction of François Bougard (74). The town was built in 854 AD at the behest of Pope Leo IV and in 1416 it was listed as destroyed and uninhabited in an inventory of papal territories. The area III is located on the slope of the hill at the eastern urban gate. Excavations have brought to light several buildings with different functions, whose life has developed into a narrow chronological range. Although evidence from the late 9th century AD has been identified, the most consistent is dated to the second half of the 14th century AD. A huge quantity of animal remains (>10,000 fragments) were found, among them pigs are largely dominant (75). The samples analyzed are: GL326, GL345, GL367, GL372, GL438, GL456, GL464.

Chagar Bazar, Syria
Contact: Augusta McMahon
Chagar Bazar lies on the west bank of the Wadi Khanzir (Pig Wadi), a tributary of the Khabur River in the Jezirah region of northeast Syria. The mounded portion of the site is approximately 12 ha, with a maximum height of 21 m above plain level. It was occupied from the 8th through early 4th millennia BP. The domestic pig specimen analyzed (AA053) comes from domestic rubbish dating to the final occupation in the early 4th millennium BP, when the site was an administrative node within a small regional state, the Kingdom of Upper Mesopotamia.

Chamalevri, Greece
Contact: Anastasia Papathanasiou, Mike Richards
Chamalevri is a site in the Bolanis field in the village of Chamalevri, located not far from the coast and 12 km east of Rethymnon in west Crete. The finds date from Middle Minoan IA (ca. 4,160-4,000 BP) to Late Minoan IIIC1 (ca. 3,190-3,130 BP). Chamalevri is of great importance because a Middle Minoan aromatic workshop was discovered at the site through organic residue analysis carried out on Middle Minoan IA sherds by the late Prof. Curt Beck, (Vassar College, USA). The site was excavated in 1992 by Dr. Maria Andreadaki-Vlazaki, General Secretary, Hellenic Ministry of Culture. The two samples (GL1086, GL1087) from this site dates to 1,775-1,650 BC.

Chedworth Roman villa, England
Contact: Andy Hammon
Chedworth Roman villa is a major Romano-British complex situated at the head of a small valley that leads directly into the valley of the River Coln in the Cotswolds. The building began as a simple villa in the first half of the 1st century AD, partially burnt down in the late 2nd century AD and was immediately rebuilt and extended. By the 4th century AD, a garden court had been created with the addition of more rooms and verandas. The villa continued to increase in luxury with a larger bath suite, Turkish-style baths and a water cistern, which was turned into an elaborate shrine. The dining rooms were enlarged and improved and up until the late 4th century the villa complex became steadily more opulent. It was eventually abandoned in the early 5th century AD (76). The one specimen from this site (GL527) is a wild boar.

Ciechrz 25, Poland
Contact: Arkadiusz Marciniak

Cierchz 25 is a Neolithic and Early Iron Age site in Kujavia in the Polish lowlands. The site was discovered in the 1990s during the rescue excavation project preceding the construction of the Yamal pipeline from Siberia to western Europe. The major discoveries at this partially excavated site comprised the Globular Amphora Culture settlement represented by pits and postholes as well as the Corded Ware Culture burial ground. Four specimens provided successful amplification of their DNA, two of them represented Neolithic (AA136, AA138), one Early Iron Age (AA137) and one Early Medieval period (AA139) (77).

Climente II, Romania
Contact: Clive Bonsall, Adina Boroneanț
Climente II (44.59001° N, 22.25763° E) is a cave site in the upper gorge of the Iron Gates region, in southwest Romania. The cave was excavated in 1968-9. Human remains were recovered from contexts identified as "Clisurean" (Final Epigravettian), along with ~6,000 chipped stone artifacts, bone tools including awls, arrowheads and a fragment of a harpoon, and shell and animal tooth ornaments. 14C dates for the Climente II main burial and animal remains from the "Clisurean" layer fall in the Bølling-Allerød Interstadial and the early Younger Dryas, between ~14,460-12,780 cal BP (78); (79). Comparison of the Climente II archaeological inventory with that from later, fisher-hunter-gatherer settlements in the Iron Gates indicates continuity of mortuary ritual, lithic tradition and subsistence practices from the Lateglacial into the Early Holocene. The two samples in this study (both wild boar - LG411_R1, LG412_R1) originate from the alleged "Clisurean" cultural layer (80); (81).

Coppergate, England
Contact: Terry O'Connor
Coppergate, York, is located between the historic centre of the city and the River Foss, to the south-east of the Roman fortress. Excavations in 1976-81 revealed 9th to 14th century settlement at urban density, based on four tenement plots set out in the early 10th century and most intensively occupied through the 10th to 11th centuries. Later levels include extensive refuse spreads, and the stratigraphy was truncated by modern construction. The specimens reported here (AA301, AA302) are from a securely late 12th century refuse spread (82).

Dęby 29, Poland
Contact: Arkadiusz Marciniak
This is Mesolithic hunter-gatherer site of the Janisławice culture in Kujavia, located in the Polish lowlands. The site yielded over 3,100 items made of chocolate flint. Specimens were imported from its outcrops located in the Holy-cross Mountains in southern Poland, ca. 250 km from the Dęby site (83); (84). The one sample analyzed (LG723) dates to the LBK period.

Derekh Namir, Israel
Contact: Liora Kolska Horwitz
Derekh Namir was a short-lived late Chalcolithic village (late 7th–early 6th millennium BP), located on the outskirts of the city of Tel Aviv in central Israel along a fluvial channel that led into the Ayalon River. Architecture was ephemeral but included several deep and narrow human-made shafts as well as a constructed water-well head, Based on examination of the animal bones, flint tools and grinding stones found at the site, the subsistence of the Chalcolithic inhabitants was based on a mixture of

agriculture, caprine and cattle husbandry, as well as hunting and fishing. The pig remains analyzed in this study (AA039 and AA044) are domestic Near Eastern pigs.

Direkli Cave, Turkey
Contact: Benjamin Arbuckle
Direkli Cave is located in the central Taurus mountains of southern Turkey at elevation of 1,100 meters a.s.l. in the province of Kahramanmaras. Excavated by Dr. C.M. Erek since 2007, the site has revealed a prehistoric sequence dating to Epipaleolithic period. Radiocarbon dates for the Epipaleolithic levels place the occupation at the Terminal Pleistocene between 14,000-11,000 cal BP (OxA-34195; OS-79556; OS-79558; Beta-276742; Beta-414912; Beta-414913). These dates are supported by a lithic assemblage dominated of microliths with parallels to the Natufian industry of the Levant.

The remains of hearth features, round structures, baked clay figurines, as well as a human burial have been uncovered in these levels. Based on analysis of the faunal remains the cave was used as a seasonal (summer/fall) campsite associated with hunter-gatherers in the region exploiting upland resources including primarily wild goats (38%) and secondarily deer (4.1%) and also large numbers of tortoise remains (35%). Wild boar represent less than one percent of the recovered faunal assemblage (N=6). Three specimens (AA522, AA523, AA524) recovered from the 2013 excavation season were identified as wild boar (based on the age of the deposits), *Sus scrofa*, and were submitted for ancient DNA analysis (DM2992 maxilla; DM2401 second phalanx [GL=28.9mm, Bp=20.7; SD=17.2; Bd=17.9]; DM3757 tarsal III). The Direkli Cave faunal assemblage is curated on site at the project dighouse, Döngel, Kahramanmaras, Turkey. Specimens were exported with the permission of the Kahramanmaras Museum and the Turkish Ministry of Culture and Tourism (85); (86).

Drama-Gerena and *Drama-Merdžumekja, Bulgaria*
Contact: Norbert Benecke
The two prehistoric settlements, Gerena and Merdžumekja, are located at the Southern and Northern bank of the river Kalnica close to the village of Drama in Thrace, Southeastern Bulgaria. The older settlement Drama-Gerena dates to the Middle Neolithic with three defined settlement phases (A-C) which can be paralleled with Karanovo II-IV. The seven animal samples analyzed here (Dra14, Dra15, Dra16, Dra17, Dra18, Dra19, Dra20) come from the older layers A and B dating to around 7,500-7,200 cal BP. The faunal assemblage is dominated by domesticated animals, especially by sheep and goat (up to 77% in phase A). Pigs are particularly rare with only about 7% among all domestic remains (87). Four domestic of the seven *Sus* remains analyzed provided successful amplification of their partial mtDNA.

Drama-Merdžumekja is a Late Neolithic settlement only 300 m apart from Drama-Gerena. Its most significant cultural horizons can be paralleled with Karanovo V-VI (6,800-6,400 cal BP). Just as in Gerena, the faunal assemblage is of mostly domesticated origin (94%) but is dominated by cows (around half of the remains), followed by the small ruminants. Pigs only played a minor role (around 10%). However, compared to Merdžumekja, wild boar are more frequently found among the game animals, pointing towards a significant environmental change in the area (87). Of the ten domesticated animals analyzed here (Dra31, Dra32, Dra33, Dra38, Dra41,

Dra45, Dra46, Dra47, Dra48, Dra49), four could be successfully analyzed for their partial mtDNA.

Durostorum-Ostrov Farm 4, Romania
Contact: Adrian Bălășescu
Durostorum-Ostrov Farm 4 site is located at 132 km by the DN3A road from Bucharest to Constanța, in southern Drobrudja. The Roman site (dated to the 2th -3th centuries AD) is considered to be part of the civilian crafts settlement that had close economic and possibly administrative ties with the ancient (Roman) town of Durostorum, located some 3.5 km west (88). The present sample (AA084) is a domestic pig bone comes from Pit 33 (89).

Durrington Walls, England
Contact: Umberto Albarella
Durrington Walls is a large Neolithic henge 3 km northeast of Stonehenge. Its encircling bank and ditch, 440 m in diameter, were constructed over the remains of a large Late Neolithic village consisting of many square-plan houses and a series of ceremonial timber circles. Large quantities of pig and cattle bones were recovered during excavations in 1967 and 2004-2007, most of these from pits, middens and postholes as well as house floors (90); (91). The village was in use in the decades around 4,500 BP, replaced by the henge earthworks around 4,450 BP. Its period of occupation coincides with the building of Stonehenge during its second stage when the iconic stone circle broadly took the form in which it appears today. Durrington Walls Neolithic village is likely to have been occupied by the builders of Stonehenge and has produced much evidence of feasting, especially during the winter season (92); (93); (94); (95). The samples analyzed are: VEM183, VEM184, VEM185.

Eggolsheim, Germany
Contact: Joris Peters
The site of Eggolsheim is located in the so-called Magna Germania, a region encompassing north-central Europe and situated north of the Roman frontier. Inhabited by a Germanic tribe, site occupation lasted from the 2nd until the 5th century AD. Contributing slightly less than 50% of the total livestock sample identified, raising pigs must have been a major economic activity at the site. Its husbandry certainly benefited from the extensive woodlands characterising the local landscape and site catchment, populated in late Antiquity by varied large game species including aurochs, European bison, elk, red deer, brown bear, and wild boar (96). The specimens analyzed (LG638, LG639, LG640, LG641, LG643, LG6414) date between the 4th to the 6th century AD.

Eilsleben, Germany
Contact: Hans-Jürgen Döhle
Eilsleben is situated 25 km southeast from Magdeburg, the capital of the federal state of Saxony-Anhalt. It is an enclosed settlement of LBK (LBK enclosure) dated ca. 7,500 up to 6,900 BP (all pig samples are LBK). Excavations of the former Landesmuseum für Vorgeschichte (County Museum of Prehistory) Halle were carried out between 1974-1990. The area excavated/investigated is 15 ha in total. Moreover further neolithic settlement traces were detected coming from: Stichbandkeramik, Rössener Kultur, Bernburger Kultur as well as burials from Aunjetitzer Kultur (97). The samples analyzed (AA460, AA461, AA462) date to the LBK era.

El Portalón (Portalon Cueva Mayor), Spain
Contact: José-Miguel Carretero, Cristina Valdiosera
The El Portalón is an Holocene archaeological site in the Sierra de Atapuerca, a hill located 15 km east of the city of Burgos (Spain). The Sierra forms part of the foothills of the Iberian mountain range in the area known as the "Corredor de la Bureba", which connects the northern Iberian Meseta and the Ebro river valley. Within the Sierra, there is a karst system reaching 3,700 m in length, known as the Cueva Mayor-Cueva del Silo Karst system. Inside this system, numerous archaeo-paleontological sites have been discovered with records from the Pleistocene (98) and the Holocene (99).

Among the Holocene archaeological sites found in the Cueva Mayor-Cueva del Silo, the site of El Portalón, located at its current entrance, is of particular interest due to the large stratigraphic sequence (exceeds 10 m of potential) starting in the Late Pleistocene and showing evidence of human occupation throughout the Holocene. Its complex formation is the result of a combination of natural processes, domestic anthropogenic activities, farming and funerary ritual practices (100); (101); (102). A reported detailed radiocarbon dates for the entire stratigraphy range from 30,000 BP to 1,000 BP and the Holocene phase of El Portalón, includes Mesolithic, Neolithic, Chalcolithic, Bronze Age, Iron Age, Roman and Medieval periods of human occupation (99); (102). Previous studies have successfully retrieved DNA from both humans (103); (104); (105) and faunal (106); (107) remains from the El Portalón site. The samples analyzed (AA509, AA510, AA511, AA512, AA513) date from the late Neolithic to the Medieval period.

Erbaba Höyük, Turkey
Contact: Benjamin Arbuckle
Erbaba Höyük is a low mound site located on alluvial deposits on the east side of Lake Beysehir, approximately 10 km northwest of the city of Beysehir, southwest Turkey at an elevation of 1,150 m. It was excavated by J. Bordaz as part of the Beysehir-Sugla Project during four seasons from 1969 to 1977 (108); (109); (110). The site represents the remains of a small Pottery Neolithic village settlement of approximately 0.5 ha dating to 8,500-8,000 BP. Ceramic and architectural parallels link the lower stratigraphic level (level III) of Erbaba to the upper levels at levels at Çatalhöyük (VIII-0), whereas the upper layers of the site (II and I) have close ceramic and figurine parallels with the late Neolithic levels at Hacilar VI-IX (111).

A large faunal assemblage has been studied by Perkins (112), Makarewicz (113), and Arbuckle (6) and is thought to represent the remains of an animal economy focused on the husbandry of domestic sheep, goat, and cattle. Pigs represent 5-10% of the faunal assemblage and based on large body size and limited evidence for juveniles are thought to represent Anatolian wild boar (*Sus scrofa)* (114). The lack of domestic pigs in the Pottery Neolithic at Erbaba is somewhat surprising but fits within a regional pattern characteristic of central Anatolia in which domestic pigs are absent from the region for the entirety of the Aceramic and Pottery Neolithic periods (115). Eight *Sus* specimens (AA099, AA102, AA105, AA106, AA107, AA108, AA109, AA110) were submitted for ancient DNA analysis. The Erbaba Höyük faunal assemblage is curated at the Peabody Museum, Harvard University.

Exedra of Crypta Balbi, Italy
Contact: Jacopo de Grossi Mazzorin, Claudia Minniti
Excavations at the exedra of Crypta Balbi (Rome) were conducted by the *Soprintendenza Archeologica di Roma* in the 1990s under the direction of Lucia Saguì (University of Rome La Sapienza (116)).The Crypta Balbi is a portico of a Roman theatre (Balbo theatre) that was originally built under Augustus in the 1st century BC and it remained functional until its abandonment in the 5th century AD (116). In the following centuries, the area was mainly used for rubbish disposal and the exedra became occupied by a midden (116); (117). This was largely composed of material probably deriving from the nearby monastic complex of San Lorenzo in Pallacinis. In addition to the huge number of animal bones (>15,000 fragments), the midden produced a large variety of different finds, ranging from pottery to coins, metals and glass remains (118); Saguì (117). The material (GL707, GL723), is dated between the 7th and the 10th century AD.

Faimingen, Germany
Contact: Joris Peters
Faimingen, Roman Phoebiana, was a Roman military station north of the Danube River erected ca. 70 AD. It secured the river's left bank and was essential for supplying soldiers and settlers during territorial expansion further north. Phoebiana thus developed into a major traffic junction for the transportation of persons and goods along and across the Danube. Roads leaving Faimingen also linked up with the Via Claudia Augusta, the major road connecting the Province Rhaetia with northern Italy and the valley of the Po River across the Alps. Site occupation at Phoebiana came to an end by the middle of the 3rd century AD, when raids by Germanic tribes forced troops and civilians to abandon Roman territory north of the Danube and withdrew south of the river, which became the new border. The large faunal assemblage still awaits documentation (119). The samples analyzed include LG654, LG655, LG657, and LG658.

Flynderhage, Denmark
Contact: Kim Aaris-Sørensen, Peter Rowley-Conwy
Flynderhage is a large Mesolithic Ertebølle site on the interior part of Norsminde Fjord, on the east coast of Jutland. Flynderhage is the largest type of coastal settlement referred to as Køkkenmøddinger, which are characterised by a dominant shell refuse mixed with cultural debris such as flint, bone, antler, charcoal, ceramics, ash, firecracked stones ('potboilers'), and features such as hearths, pits, stake-holes and graves (120). The midden comprises mainly oyster shells, so the fjord was more saline in the Late Mesolithic than it is today. Adjacent to other large and small Ertebølle shell middens including Norsminde. Few features were found but the midden contained many sherds of pointed-based Ertebølle ceramics and bones of wild mammals. No domestic mammals were present except dog. The three samples (AA017, GL429, GL907) analyzed date to the Late Mesolithic and of are of wild boar.

Fontbrégoua, France
Contact: Lionel Gourichon
Fontbrégoua is a large cave located in the Provence region, southeastern France, ca. 50 km from the Mediterranean coastline. Mainly occupied during the Early and Middle Neolithic (ca. 7,400-5,600 BP), the site yielded rich material culture attributed

to the Late Cardial and Chassey cultures (121); (122). 20 of the 36 selected specimens provided successful results from the DNA analysis. Among the 6 specimens dated to the Cardial period (ca. 7,300-7,050 BP), 5 belong to domestic pigs (LG188, LG194, LG195, LG197, LG200), and the other is wild boar (LG210). Regarding the following phases (Post-Cardial and Chassey), the animals studied were mostly identified as domestic (LG204, LG211). The other samples analyzed include: LG360, LG361, LG363, LG367, LG369, LG370, LG370).

Franchthi Cave, Greece
Contact: Anastasia Papathanasiou, Mike Richards
Franchthi cave is located at south-western Argolid, Greece, at the tip of a limestone peninsula, overlooking a coastal plain. It is about 120 meters long and it was used for approximately 25–30,000 years spanning the Upper Palaeolithic to the end of the Neolithic (123). The site from the Early Neolithic includes also Paralia, an adjusted, open air settlement. It was excavated in the 1970s and 1980s by Thomas Jacobsen and Indiana University and has yielded evidence for occupation and mortuary ritual as implied by a well preserved human osteological assemblage, a wide spectrum of plant and animal remains, stone tools as well as pottery in the Neolithic. It also yielded evidence for seafaring in the Paleolithic and the Mesolithic as implied by the obsidian from Melos Island and by the numerous tuna vertebrae. The two samples analyzed are GL1081 and GL1082.

Friesack, Germany
Contact: Bernhard Gramsch
Friesack is located in northern Germany, Havelland district of Brandenberg state, and contains 2 Mesolithic marshland sites, Friesack 4 and Friesack 27. The sites are in the westernmost part of the Warsaw-Berlin ice-marginal valley, and filled with fluvioglacial sand and peat. A multi-stratified sequence of sediments consisting of peat muds, sands, and humose sands indicates many Mesolithic occupations from the middle Pre-boreal (~9,700 uncal BP) to the middle Atlantic (~6,600 uncal BP). Flint, stone, wood, bark, and zooarchaeological artefacts of bone, antler, teeth, have been found, in addition to bast including fragments of nets, strings and ropes (124). The wild boar samples analyzed are GL122 and GL538.

Frilli W, Italy
Contact: Domenico Lo Vetro, Lucia Sarti
Frilli W is a multiphase site, part of a broad settlement unit that is positioned in the east of the area of Sesto Fiorentino. It is located in the upper part of the alluvial plain close to the foothill side of Monte Morello. It is a multi-stratified site with evidence spanning from Roman to Bell Beaker period. The Roman, Iron Age, Middle Bronze Age, Early Bronze Age and Bell Beaker phases are separated by alluvial layers and spread over a wide Settlement Unit. Samples are related to Middle Bronze Age dwelling activities. (125). Five wild boar specimens were analysed: AA630, AA631, AA632, AA633, AA634.

Ganj Dareh, Iran
Contact: Melinda Zeder

Ganj Dareh is a small aceramic Neolithic upland (1,400 m) site located in Iranian Kurdistan consisting of five building levels occupied over a relatively brief period (c. 2-300 years) at about 9,900 cal BP (126); (127). Demographic evidence points to initial management of morphologically wild goats at the site. One specimen (AA046) dates to the Pre-Pottery Neolithic.

Glaumbaer III (Skagafjordur), Iceland
Contact: Mike Church
The 2 pig bones sampled (AA400, AA401) were recovered from midden material associated with the occupation of a Norse farmstead. The midden was dated by 14C and tephra to between 984-1104 cal AD (128).

Göbekli Tepe, Turkey
Contact: Joris Peters
Göbekli Tepe is an aceramic Neolithic site dating to the early 12th millennium BP and 11th millennium BP. The site is not only outstanding for its unique stone architecture with conspicuous T-shaped pillars, but also because of its various animal and other motifs carved on the pillars that are illustrative of a complex belief system (129); (130). The faunal assemblage hitherto studied comprises more than 60 wild mammal and bird taxa. So far, Göbekli Tepe's fauna did not provide indications of efforts to stable and/or manage ungulates, which is in contrast to the site's wider surroundings, where management and early domestication of ungulates are well underway by the second half of the 11th millennium BP, e.g. at Çayönü (131) and Nevalı Çori (132). The two specimens analyzed are AA317 and AA318.

Gohar Tepe, Iran
Contact: Marjan Mashkour
Gohar Tepe is located in the Mazandaran Province in northern Iran, southern Caspian Sea (36°40'42"N 53°24'07"E). The site was excavated by A. Mahforouzi (ICCHTO). The site was occupied during the Bronze and Iron Age, however Late Chalcolithic, (middle of 6th millennium BP) remains also very occasionally found. The settlement reached more than 10 ha in the Middle Bronze Age, and some parts were used as a cemetery during the Late Bronze Age. Various sources of subsistence based mainly on agro-pastoralism with additional input of wild marine and terrestrial resources (133); (134). The specimens analyzed are AA027, and AA028, which were determined to be wild boar.

Gomolava, Serbia
Contact: David Orton
Gomolava is a large multi-period *tell* settlement on the left bank of the river Sava in Vojvodina, Serbia, near the village of Hrtkovci and around 60 km west of Belgrade. Around 5,000 m^2 were excavated out of an estimated original area of 13,800 m^2 [(135)], much of which was lost to river erosion prior to excavation. The specimens included here are from the first phase, Gomolava I, belonging to the late Neolithic/Copper Age Vinča group. Three subphases were identified (135) and have subsequently been radiocarbon dated (136); (137):
Ia (Vinča B2-C; c.6,900-6,800 cal BP)
Ia-b (Vinča C; c.6,850-6,750 cal BP)
Ib (Vinča D; c.6,800-6,600 cal BP)

Occupation was probably continuous throughout this date range. Where Gomolava Ia is characterised mainly by large pits, Gomolava Iab features large houses recognised from post-holes, and Gomolava Ib has a large number of smaller but densely packed burnt houses. Gomolava Ib also features the only known intra-settlement cemetery from the Vinča period, probably representing the latest phase of Neolithic activity at the site.

Animal bones have been studied from two areas, Blok I (138) and Blok VII (139). In each case wild animals represented between a third and a half of the identified mammal bone assemblage, with (domestic) cattle, red deer, and pigs being the three most important taxa. Of the pigs, the relative numbers of identified as wild and as domestic vary considerably between the two studies, but each form appears to have been present in roughly equal quantities overall. In Blok VII, wild pigs appear to have been preferentially deposited in pits. Specimens AL1062, AL1068, and AL1115 are domestic pigs; AL1063, AL1070, AL1110, AL1112 are wild boar; and the other undetermined specimens are: AL1109, AL1111, AL1113, and AL1114.

Gordion, Turkey
Contact: Melinda Zeder
Gordion is a large multi-component site located in Central Anatolia 80 m southwest of Ankara. Capital of the Iron Age Phrygian Empire (3,200-2,550 BP) the site also has levels dating back to the Late Bronze Age Hittite period up through the Roman era with a major Galatian occupation of the site (140). Pigs were a particularly important component of the animal economy at Gordion in the Middle and Late Phrygian periods (2,800-2,330 BP) (141). Six specimens (two Late Bronze - AA087, AA089, two Late Phrygian - AA088, AA090, one Hellenistic - AA091, one III-I century BC - AA087) identified as domestic pig on the basis of size and demographic profiles were successfully analyzed.

Griesstetten, Germany
Contact: Joris Peters
Griesstetten is a final Neolithic settlement pertaining to the Cham culture dating 5,500-4,700 BP. Excavated between 1988 and 1991 the site produced a large assemblage of faunal remains. The dominant species are cattle and red deer, followed by pigs. Other game species exploited for food include roe deer, aurochs, wild boar, brown bear, and beaver. Two distinct size groups can be observed in the *Sus* assemblage, a smaller one considered domestic and a larger one considered wild (142). One wild boar was analyzed in this study (LG622).

Grotta del Santuario della Madonna, Italy
Contact: Antonio Tagliacozzo
The cave is located at Praia a Mare (CS), in Northern Calabria, Southern Italy. The archaeological deposit, over 8 m thick, evidencing human frequentation from the Upper Palaeolithic to the Middle Ages. The Mesolithic layers are characterized by the presence of hearth structures, shell middens, lithic industry and bone remains of wild mammals, tortoises and malacofauna. The lithic assemblage, with very low microlithic component, is known as Undifferentiated Epipalaeolithic. The new dates frame the Mesolithic levels between 10,430 and 9,040 cal. BP. Only a tooth of wild boar (GL1000) from Mesolithic layers were successfully analyzed in this study.

Grotta Verde (Alghero, Sardinia), Italy
Contact: Domenico Fulgione, Barbara Wilkens
Grotta Verde (Alghero, Sardinia Italy) is located in the northwestern part of Sardinia. The entrance of the cave is situated 75 m above present day sea level, on the steep east slope of Capo Caccia, which is a coastal promontory (containing several karstic caves) next to the bay of Porto Conte. Underwater excavations in the chamber revealed a series of human burials, along a wall of the chamber, in the natural niches and in rock cavities. On the walls, graffiti as well as other undisputed signs of a human presence are still recognizable. The sample AL612 collected in Grotta Verde, dates back to 5,500-4,700 cal BP, and is a humerus of a domestic pig.

Gudnja, Croatia
Contact: Preston Miracle
Within the East Adriatic, the first traces of the Neolithic by impressed Ware pottery and domesticated animals are found in caves on the Adriatic islands just before 8,000 BP (143). Gudnja is a cavern site within the base of the Pelješac Peninsula, Dalmatia, in the southern end of the Adriatic. Gudnja culture, distinguished by Impressed Ware pottery, is stratigraphically well-defined, and a completely self-contained cultural phenomenon in Neolithic Dalmatia, as characterised by unique ceramographic and iconographic properties. Neolithic Gudnja is dated to 9,170 ± 70 BP, [GrN-10315; 1 sd range 8,160-7,920 BP] (144); (145); (143). The specimens analyzed (LG343_R1, LG344_R1, LG346_R1, LG347_R1) date to the Neolithic.

Guran Tepe, Iran
Contact: Melinda Zeder
Guran Tepe is a Neolithic age site located in upland (c. 700 m) elevations in Luristan, Iran (146). Animal economy in Aceramic levels at the site (c. 9,000 cal BP) is dominated by managed goats with a mix of wild animals, including wild boar, while ceramic Neolithic age levels at the site (c. 8,500 cal BP) have evidence of the introduction of domestic sheep and an increased emphasis on the hunting of gazelle (147). Two specimens (AA123, AA124) from ceramic Neolithic levels were analyzed here, both presumed to be wild.

Gürcütepe, Turkey
Contact: Joris Peters
Gürcütepe is located south to Göbekli Tepe, in the Harran plain, at the altitude of about 450 m a.s.l.. A total of 5 pig specimens (AA319, AA320, AA321, AA322, AA323) from Gürcütepe, dated to the 10th millennium BP, were analyzed in the present study, providing no successful amplification of DNA (148); (149).

Haftavan Tepe, Iran
Contact: Azadeh Mohaseb and Marjan Mashkour
Haftavan Tepe is located in the middle of the small plain of Salmas, in the northwestern part of Lake Urmia in the province of Western Azerbaijan in Iran. Haftavan Tepe is one of the largest protohistoric urban sites in the region, excavated during the '70s. Architectural remains from the Early Bronze Age (4,300-3,800 BP according to the original dating of the site) through to the beginning of the Islamic period were uncovered and show strong ties with Caucasus. A very large assemblage of animal bones was studied within a PhD project by F.A. Mohaseb and supervised by M. Mashkour that constitutes a reliable source of information in particular for Bronze

Age and Iron Age subsistence economy and pig domestication in this part of the Iranian Plateau (150–152). The samples analyzed include the following: AA360, AA361, AA362, LG799.

Hallan Çemi, Turkey
Contact: Melinda Zeder
Hallan Çemi is an Epipaleolithic (c. 11,600 cal BP) site in the Batman drainage basin of southeastern Anatolia (153). Among the earliest sedentary communities in the region occupied year-round, the site consisted of an estimated 10-15 round houses arrayed around a central activity area. Despite the site's relatively brief occupation (200-300 years), salvage excavations yielded an exceptionally large faunal assemblage (estimated at approximately 2 metric tons), consisting of a diverse array of large and small game species. Initial arguments for early pig management at the site (154) are currently under review. The following specimens were analyzed: AA047, AA048, AA063, AA066, AA100, AA101, AA103, AA104, AA180, BLT001, BLT002, BLT003.

Hârşova-tell, Romania
Contact: Adrian Bălăşescu
Hârşova-*tell* is located on a terrace on the eastern bank of the Danube River in southeast Romania (Constanţa County). One of the largest *tells* in Europe, the thickness of the stratigraphic sequence has ca. 12 m. After to the development of the *tell*, the Danube moved its course northwards, substantially eroding the settlement. The stratigraphic sequence is very complex, ranging from the Late Neolithic (Hamangia and Boian cultures) to the Late Eneolithic (Gumelniţa and Cernavodă I cultures) (155); (156). The present bone sample (*Sus* sp., AA081) comes from the Cernavodă I levels (5,700-5,300 cal BP - (157)).

Hassek Höyük, Turkey
Contact: Bea de Cupere
Hassek Höyük. Hassek Höyük is located in the Karababa Basin in the Urfa and Adiyaman provinces in southeastern Anatolia. Altitudes range between 400 and 600 m. Studies of the faunal material has been published (158); (159); (160). Based upon stratigraphical sequencing of the sites and AMS radiocarbon dating, pig samples from Hassek Höyük were dated to the Chalcolithic and Early Bronze Age. A total of 25 specimens from Hassek Höyük were analyzed, of which three (AA312, AA313, AA314) yielded positive and reproducible amplifications of DNA (161); (158); (159); (160).

Herxheim, Germany
Contact: Andrea Zeeb-Lanz, Rose-Marie Arbogast
The early Neolithic site of Herxheim, (Rhineland-Palatinate, Germany), occupied between 7,300 and 6,950 BP, has left vestiges (pits and houses) of a occupation attributed to Linearband culture surrounded by a double earthwork. Herxheim was "normally" inhabited during the 350 years of the Bandkeramik culture left of the river Rhine, but at the end of its existence the site drastically changed its function and became a central ritual place where more than 1,000 humans were sacrificed and their skeletons smashed into small fragments. Together with equally destroyed high quality pottery, stone implements and grinding stones the rests were then disposed of in huge concentrations in a double ditch around the settlement; the parallel ditches had been

dug to receive the rests of the rituals which lack any tradition in Bandkeramik behaviour and were also not repeated in later cultures.

The faunal remains from the different features amount to a little less than 10,500 pieces. Remains of pig form an important part of these assemblages, and represent most often the most intensely exploited domestic animal after cattle. Boar hunting was also regularly practiced, as attested by the consistent. contribution of this animal in all assemblages. These characteristics reflect the key role occupied by the exploitation of the suinae in the economy of this early neolithic occupation. The Neolithic specimens analyzed are KD032, KD033, KD034.

Homolka, Czech Republic
Contact: Richard Meadow, Peter I. Bogucki
Homolka is a fortified hilltop settlement of the Řivnáč Culture in central Bohemia located 21 km northwest of Prague. It was excavated in 1929-31 and 1960-61. The Řivnáč Culture is dated ca. 5,100-4,800 BP. Homolka is a rounded hill of Algonkian filite, a soft and flaky shale with occasional outgroups of quartz. It which rises to the 20 m high above the narrow valley east of the village of Stehelceves in the county of Kladno. The floor of the valley is rich with fertile alluvial deposits. The top of Homolka resembles a roughly pointed horseshoe, the widest point approximately 100 m long and a width of 60 m. Two successive palisade systems enclosed an area of approximately one hectare that contained about two dozen small post structures surrounding an open area. Pits and postholes yielded samples of animal bones and artifacts. Animal bones found at the site include the following: domesticated -- cattle, pig, sheep, dog, and possibly cat; wild: red and roe deer, red fox, beaver, badger, cat, otter, hare, bear, horse; birds: goose, crane, black cock (synsacrum of a large gallinaceous bird); miscellaneous: frog, hamster (162); (163); (164). The specimens analyzed from this site include: GL37and GL38.

Houten-Hoogdijk, Utrecht, Netherlands
Contact: Youri van den Hurk
During excavations in Houten-Hoogdijk, Utrecht in 1999-2000, eight farmhouses were discovered. These farmhouses were dated to the 12th-13th century AD. The faunal remains, as well as the pottery and metal finds, suggest that the farmers were not particularly rich. The sample taken from this site (KD024) also dates to the 12th-13th century AD (165).

Hrísheimar, Iceland
Contact: Mike Church
The 16 pig bones (SI003, SI004, SI006, SI007, SI008, SI009, SI010, SI011, SI012, SI013, SI014, SI015, SI016, SI017, SI018, SI019) were recovered from a sheet midden, associated with turf buildings of Viking age. The midden immediately overlay the Landnám tephra (dated to 871±2 cal AD - (166) and was in turn overlain by V-Sv tephra (dated to c. 940 cal AD - (167)), producing a date range of 870-940 cal AD for the material. This range corresponded very well with 14C dating of bone material from the midden layers (168).

Icoana, Romania
Contact: Adina Boroneanț, Clive Bonsall

Icoana (44.65167° N, 22.30056° E) in the Iron Gates gorge in southwest Romania was an open-air site located on a narrow strip of land bordering the Danube. As with most sites in the area the site was flooded in 1971 during construction of the Iron Gates I dam and hydropower plant. The site was pluristratified but the Holocene deposits had been extensively eroded by the Danube prior to archaeological investigation, leaving traces of an Early Neolithic Starčevo-Criş culture (mainly pit features and sunken huts) and Mesolithic occupation (trapezoidal- and rectangular-plan dwellings). The faunal remains originated mainly from the identified features rather than the so-called cultural layers. The samples included in the present study were assigned stratigraphically to the Middle Mesolithic, radiocarbon dates for this phase ranging between ~10,800–9,500 cal BP (169). The two directly dated samples (BLT006/ICO-07, BLT008/ICO-09; (170)) fall into this time range (80); (81). The other samples analyzed (BLT007, BLT029, BLT029) also date to this period.

Jarmo, Iraq
Contact: Max Price
Qalat Jarmo is a 1.3 ha site located in the foothills of the Zagros Mountains, near the town of Chemchemal in northern Iraq. The occupation of Jarmo straddles the final PPNB and early Pottery Neolithic (late 10th to early 9th millennia cal BP). The site provides some of the earliest evidence for domestic pigs in the Zagros region (171); (172); (173); (174). The specimens analyzed are AA057, AA060, and AA061.

Kanlıgeçit, Turkey
Contact: Norbert Benecke
Kanlıgeçit is a multiphased Bronze Age tell in the eastern part of Turkish Thrace close to Kırklareli and southeast of the Neolithic tell of Aşağı Pınar. In contrast to Aşağı Pınar, cattle are dominating the faunal assemblage of the Bronze Age settlement, followed by goat/sheep and pig (one out of four each) (23). The five wild boar (Kan14, Kan15, Kan16, Kan17, Kan18) and six domestic pigs (Kan8, Kan9, Kan10, Kan11, Kan12, Kan13) analyzed here are from Early Bronze Age Layers (II-III) . All but one wild boar could be successfully analyzed for their partial mtDNA.

Karlburg, Germany
Contact: Joris Peters
Early medieval Karlburg was one of the castles granted by King Pippin to the bishopric of Würzburg founded in 741-742 AD. Occupation lasted until the 13th century AD. Excavations were carried out in the castle and associated valley settlement. Faunal analysis revealed differences between the two contexts. Obviously, the castle's inhabitants preferred the meat of juvenile pigs and cattle as well as poultry and venison (red deer), whereas the farming community in the valley had to be content primarily with the meat of aged cattle, pigs, and even horses, although forbidden by the church (175). Three domestic pigs (LG624, LG625, LG626) from the Late Medieval period were analyzed here.

Khatunarkh, Armenia
Contact: Ninna Manaseryan
Khatunarkh is a site in Etchmiadzin region dated by the 7th and beginning of the 6th millennium BP. The excavations are made by R. Torosyan. Khatunarkh is located in the Etchmiadzin region, the Ararat plain. Echmiadzin (Vagharshapat) is the 4th-largest city in Armenia and the most populous municipal community of Armavir Province,

by about 18 km (11 mi) west of the capital Yerevan, and 10 km (6 mi) north of the closed Turkish-Armenian border (176). One wild boar specimen (AA113) was analyzed.

Klimonas, Cyprus
Contact: Jean-Denis Vigne, Thomas Cucchi
Klimonas (Ayios Tychonas, Limassol District; 137 m a.s.l.) is an open-air site located on a sea facing hillslope overlooking a small valley abundant in high quality flints, less than 3 km from the South modern seashore of Cyprus. The huge material culture documentation is homogeneous and refers to a regional culture dating to the Late PPNA, principally characterized by the absence of bidirectional knapping. More than 20 radiocarbon dating (charcoal and charred seed) indicate human occupation developed here during around 10,800 cal. BP. In terms of buildings, the most striking discovery was a 10 m circular semi-embedded feature, which was interpreted as a communal building, similar to the largest ones discovered in the PPNA villages of Southeast Anatolia and the Euphrates valley (177); (178). The rest of the 20 domestic buildings of smaller size, made out of earth and trassed into the hillside, were also excavated. Together with many other aspects of the material culture and the micromorphological analyses of the mud used for the walls and floors of the buildings, scarce archaeobotanical data indicated that the Klimonas villagers were cultivating cereals, especially emmer wheat (*Triticum dicoccoides/dicoccum*), likely introduced from the continent. More than 25,000 animal remains indicate that people were hunting the only large game living on the island at that time: the small wild boar (*Sus scrofa* sp.; used in the present study), which represents more than 95% of the identified bone remains. Domestic dogs (*Canis familiaris*) and commensal cats *(Felis s. lybica*), the latter for catching mice, were living in the village.

The wild boar which was the main source of hunted animal food at Klimonas has been introduced to Cyprus ca. 12,500 cal. BP by the Epipaleolithic hunters, likely in the aim to restock the island game resources short after the extinction of the Pleistocene endemic dwarf hippo fauna (179); (180). The suspected origin is the North Levant / Southwest Anatolia. It rapidly proliferated on the island, evolving toward an insular lineage 15-20% smaller in size as the contemporaneous wild boars on the nearby continent. They were the main source of animal food until the introduction of the Mesopotamian fallow deer and the domestic ruminants, at the beginning of the 10th millennium BP (Middle PPNB), nearly 1,000 years after the occupation of the PPNA Klimonas village (181). Two Y1 wild boar specimens (AL1024 and AL1031) dating to the PPNA were analyzed. As for AL1017, AL1024, AL1024, and AL1026, it is unknown if they are wild or domestic.

Klingenberg-Schlossberg and Hetzenberg, Germany
Contact: Elisabeth Stephan
Klingenberg-Schlossberg and Hetzenberg are Michelsberg enclosures near Heilbronn in the Neckar valley in southwest Germany. Excavated in 1986-1987, the Klingenberg enclosure has two ditches across a loess promontory, traces of a palisade inside the inner ditch, remains of burnt superstructure in both ditches, numerous pits both inside and outside (182). More than 90 AMS-dates of human and animal bones, botanical remains, and charcoal suggest that the site was occupied from 40th–39th centuries to the mid-37th century cal BC (183). Eight specimens of domestic pigs (LG143, LG145, LG592, LG593, LG595, LG598, LG599, LG600) were analyzed.

The excavations of the Hetzenberg enclosure in 1966, 1989 and 1990 revealed three parallel ditches, but due to erosional processes no other features are preserved. According to radiocarbon dates and archaeological finds these enclosure was constructed earlier than the Klingenberg earthwork and used from c. 6,300-6,000 BP (182). Four specimens of domestic pigs (LG147, LG169, LG171, LG173) were analyzed.

Kohne Tepesi, Iran
Contact: Sepideh Maziar Marjan Mashkour, Shiva Sheikhi Seno, F.A. Mohaseb
Kohne Tepesi is located in the southern part of the Araxes River, in northwestern Iran. Despite its small size (0.2 ha), it contains 6 m deposits of Kura-Araxes culture (Early Bronze Age), and two burial tombs. This phase based on C14 dating, dated to the 4,636-4,339 cal BP (184). A garrison was built later on top of the Kura-Araxes remains, which dated to the Parthian period. The Archaeozoological material was studied by S. Sheikhi Seno, M. Mashkour and A. Mohaseb (in prep).

The suid assemblage of Kohneh Tepesi because of their cultural and chronological relation were analyzed both metrically and genetically (partly exploited through GMM in (161). All of the seven specimens (AA363, AA364, AA365, AA366, AA367, AA368, AA369) selected for analysis in this paper are allocated to the domestic pig and all belong to the Early Bronze Age levels. It is not known if specimens LG217, LG219, LG221, LG222 are wild boar or domestic pig.

Kolosy tér 6, (Aquincum / Budapest), Hungary
Contact: László Bartosiewicz
The Roman period settlement at Kolosy tér 6 (Budapest, Hungary), fell within the southern periphery of Aquincum, the capital of Pannonia province. It was located in the broad floodplain on the right bank of the Danube (185). Excavations brought to light floor remains in the southern section of a small building. They were found underneath a thick Roman period destruction layer dated to the 2nd–3rd century AD (186). The function of the building remains unclear. The building pre-dates the Roman finds and is thus attributable to the Celtic remains below. No zooarchaeological analysis has been carried out at the site. For this study, two domestic pigs have been analyzed (AA407 and AA410).

Koprivec, Bulgaria
Contact: Norbert Benecke
The Neolithic settlement of Koprivec lays southeast of the eponymous contemporary village at the river Baniski Lom in Northern Bulgaria (187). The two wild boar samples (Kop8, Kop9) successfully analyzed here for their partial mtDNA come from early Neolithic layers of the settlement dating to between 8,200-7,800 cal BP. They are part of a faunal assemblage that is vastly dominated by domestics (more than 80% throughout all phases), but revealed only 1-13% of domestic pig remains (63).

Kovačevo, Bulgaria
Contact: Norbert Benecke
The Bulgarian site of Kovačevo is located in the Middle Struma Valley at the foot of the Pirin Mountains close to the eponymous contemporary village. Interestingly, the Early Neolithic phases reveal Near Eastern elements additionally to Anatolian

relations (188). The domestic faunal spectrum is dominated by sheep and goat (65%), followed by pigs (almost 21%) and only few cattle (14%). Out of the 13 *Sus* remains analyzed here (Kov13, Kov14, Kov15, Kov16, Kov17, Kov18, Kov19, Kov20, Kov21, Kov29, Kov30, Kov36, Kov39) from Early Neolithic phases (8,200-7,600 cal BP), five were successfully analyzed for a partial mtDNA fragment, two of them represent wild boar.

Kuczkowo 5, Poland
Contact: Arkadiusz Marciniak
Kuczkowo 5 is a Neolithic site in Kujavia in the Polish lowlands. The site was discovered in the 1990s during the rescue excavation project preceding the construction of the Yamal pipeline from Siberia to western Europe. Remains of three Neolithic cultures were unearthed comprising: (a) LBK longhouses, (b) Late Lengyel cattle burial, and (c) round barrow burial with a palisade ditch of the Corded Ware Culture (189); (190). Six domestic pig samples from the Neolithic were analyzed: LG733, LG735, LG736, LG737, LG738, LG739.

Künzing-Unternberg, Germany
Contact: Joris Peters
Künzing-Unternberg is a middle Neolithic ditch earthwork dating ca. 6,900-6,500 cal BP. Excavated in the 1980s, the site produced a large faunal assemblage dominated by remains of red deer and cattle, while pig ranks third (191). Apart from red deer game comprised roe deer, wild boar, aurochs, brown bear, wild horse and elk, i.e. taxa indicative of extensive woodland habitat in the site catchment. Comparison with other broadly contemporaneous Neolithic *Sus* populations classified as wild or domestic using dental and post-cranial size does not point to admixture between the two forms (191), despite the fact that it could have been expected (192). Thirteen specimens were analyzed: LG148, LG153, LG155, LG156, LG159, LG174, LG255, LG256, LG628, LG269, LG633, LG634, LG635.

Kush, Ras al-Khaimah, United Arab Emirates
Contact: Derek Kennett
Kush is a small (c. 1 ha) archaeological tell close to the shore in the Shimal area of northern Ras al-Khaimah (UAE). It is located on the Arabian/Persian Gulf about 80 km inside the Straits of Hormuz close to a key maritime trade route that has operated since at least the Bronze Age. The site has been partially excavated and has yielded an occupation sequence dating from the Sasanian period (c. 5th AD) to about the mid 13th century AD (193). One domestic pig specimen (AA019) analyzed is from the Sasnian culture.

Latronico (Potenza, Basilicata), Italy
Contact: Domenico Fulgione, Barbara Wilkens
Latronico (Potenza, Italy) is a cave that opens in a travertine bench referable to the middle upper Pleistocene. The caves are located at the left side of the upper valley of the River Sinni, at about three kilometers from the town of Latronico, in the Province of Potenza (Basilicata, Italy). Evidence of Mesolithic, Castelnovian occupation has been found here. Using radiocarbon dating, four eras can be assigned to this cave, ranging from 7800±90 BP (R-449) to 7400±90 BP (R-447). The clearest and most complete stratigraphy is that of sectors III and V (grotta 3), which show the cultural

succession from the Mesolithic to the Metal Age. The sample of the present study comes from Metal Age and is a first phalanx (AL616) of a presumably wild boar.

Lchashen, Armenia
Contact: Ninna Manaseryan
Lchashen are burials close to villages Lchashen and Chkalovka. These burials are situated on the drained land in 150-200 m far from the Lake Sevan shore line, and are dated to the 4th millennium BP. Part of these burials are also dated to the Early Iron Age (the first half of the 3rd millennium BP). The excavations are made by archaeologists A. Mnatsakanyan and L. Petrosyan (129). Five specimens were analyzed: AA111, AA112, AA115, AA116, AA119.

Le Taï (Grotte du Tai), France
Contact: Stéphanie Bréhard, Jean-Denis Vigne, Allowen Evin
The site of Le Taï is located at Remoulins, in the Gard department (southern France). It is located about 125 m .a.s.l at the bottom of a ravine with steep slope in the Gardon valley, very close to the Rhône River. Intensive archaeological research have been conducted recently (194). The excavations documented a long stratigraphic sequence from Early Neolithic to Early Bronze Age. The Early Neolithic phase belongs to the Epicardial culture. Currently available radiocarbon dates indicate a continuous occupation between c. 7,250 and 7,100 cal BP. During the Epicardial phase, the site was intensively used, with occupations both inside the cave and outside. Given the diversity and density of archaeological structures (hearths, pits and post-holes) and the characteristics of the faunal, ceramic and macro-lithic remains, it is likely that the site was occupied permanently. The excavation of the Epicardial levels provided a large sample of well-preserved faunal remains: the number of identified specimens (NISP) is 3,100 for mammals. The site is characterized by a high proportion of lagomorph remains (which are food refuse) but if we exclude these small wild mammals, the faunal spectrum is greatly dominated by domestic sheep/goats and cattle (91% of the NISP). And caprines, mostly sheep, are clearly the main domestic species (71% of the NISP). Suids and wild carnivores have been identified (respectively 5% and 4% of the NISP) but they did not play an important role in the economic system. *Sus* remains mostly belong to young or subadult individuals so determine if they correspond to wild or domestic animals was not possible. The specimens examined for this site date to the Early Neolithic (AA294, AA295).

Leilan, Syria
Contact: Melinda Zeder
Leilan is large urban settlement with occupations dating to the mid-late 5th millennium and the 4th millennium BP. Early Bronze Age-Leilan consisted of an upper town with large public buildings and a surrounding lower town (195). Domestic pigs were an important component of the subsistence economy of the site. One specimen from (AA120, c. 4,300 BP) level of the lower town was analyzed (28). Two specimens from somewhat earlier levels of the upper town (AA122, AA329, c. 4,500 BP) was also analyzed.

Londerzeel, Belgium
Contact: Anton Ervynck
The castle of Londerzeel is now located in the centre of a village in the former duchy of Brabant (Flanders, Belgium). This medieval feudal stronghold originated as a

motte-and-bailey castle and later was transformed into a brick castle, belonging to the noble house of Vianden (196). From this period (end 13th- early 14th c. AD), a large collection of consumption remains has been recovered (197), including the domestic pig bones (AA280, AA281) analyzed in this study.

Ludwinowo 7, Poland
Contact: Arkadiusz Marciniak
The site Ludwinowo 7, located in Kujavia within the Polish lowlands was discovered in 2000 and excavated in the 2000s as a part of large rescue excavation project preceding the construction the A1 motorway. Its major part comprise a large LBK settlement which size is estimated to be 9.5 ha. Altogether, 14 longhouses accompanied by numerous features, including two wells and two graves, were discovered (198). Three Neolithic domestic pig specimens were analyzed (LG724, LG726, LG727).

Luncaviţa-Cetăţuie, Romania
Contact: Adrian Bălăşescu
Luncaviţa-Cetăţuie is a *tell*-settlement on a terrace near the Luncaviţa stream, 4.5 km southeast of the Luncaviţa village (Tulcea County). The stratigraphic sequence (with a thickness of 3.5 m) consists of mainly of six Gumelniţa A (phases A1 and A2) levels (199). Scarce finds were attributed to the Hallstatt period and five burials to the Late Medieval (18th century (200)). The faunal assemblage of the studied Gumelnița A2 level (ca. 6,490-6,263 cal BP) comprise both domestic and wild species, almost equally represented (201); (157) (LG800, LG802, LG803, LG804, LG805, LG806, LG807, LG808, LG809, LG811, LG812, LG813).

Madzhari, Republic of North Macedonia
Contact: Lionel Gourichon
Tumba Madzhari (or Madžari) is an open-air Neolithic site situated in the north-east neighbourhood of Skopje (Republic of North Macedonia). Recent re-excavations of the site indicate a continuous occupation during the first part of the 6th millennium (8,060-7,500 cal BP), i.e. at the end of the Early Neolithic and the very beginning of the Middle Neolithic (202). The two specimens (BLT022, BLT023) analyzed belong to domestic pigs (as identified by biometric data in comparison to samples from other Macedonian sites) with a Y1 Near Eastern signature.

Măgura, Romania
Contact Adrian Bălăşescu
The locality Măgura-Boldul lui Moș Ivănuș is part of the Măgura Buduiasca, being its earliest Early Neolithic occupational phase (ca. 8,064-7,746 cal BP). It was suggested that the earliest Neolithic communities settled first at Boldul lui Moş Ivănuş, and in time, the settlement developed at a larger scale within the Buduiasca zone. It is so far the earliest dated Early Neolithic site in southeast Romania. Two wild boar bones, from pit-features Starčevo- Criş C51 (BLT009) and C57 (LG432) were analyzed. The latter was dated ca. 8,060-7,903 cal BP (203).

Măgura-Buduiasca is located in southern Romania, 10 km north of Alexandria (Teleorman County), in the vicinity of Măgura village on the Teleorman River lower terrace. The stratigraphic sequence of the site comprises several Neolithic occupations, overlapped in some areas by remains from later occupations (e.g. Bronze

Age, Iron Age, Migration Period, and Middle Age). Archaeological excavations confirmed the following Neolithic stratigraphy: Early Neolithic (Starčevo-Criş culture), Middle Neolithic (Dudeşti culture), and Late Neolithic (Vădastra culture), covering a time span between ca. 8,064 and 7,078 cal BP. The analyzed pig bone from C22 (and attributed to the Late Neolithic Vădastra culture - BLT010) is dated 7,318-7,078 cal BP (204); (203).

Marmaray -Yenikapı
Contact: Canan Çakirlar
This is a Neolithic (ca. 8,500-7,000 cal BP) site located on the western coast of Istanbul, situated underneath a Byzantine harbor close to the southern end of the Bosphorus Channel. The settlement and its environs were investigated through extensive rescue excavations led by the Istanbul Archaeological Museum. Although the pottery and other material cultural remains display features similar to key Neolithic sites such as Fikirtepe on the eastern coast of Istanbul, burial practices show higher diversity at Marmaray-Yenikapi, and evidence for cereal cultivation is unequivocal, thanks to partial wet-preservation. Sheep, goat, cattle, and pig appear in morphologically domesticated form. It should be noted excavated areas are huge, and stratigraphic studies are still ongoing (205). One domestic pig specimen was analyzed (AL1104).

Mashnaqa, Syria
Contact: Melinda Zeder
Mashnaqa is a small settlement in the middle Khabur basin of northeastern Syria with occupations dating from the 6th to the 5th millennium BP (206); (207). One specimen (AA052) dating to Late Ubaid levels (6,600-2400 BP), presumed to be domestic pig, was analyzed.

Matarrah, Iraq
Contact: Max Price
Tell Matarrah is a Pottery Neolithic site (mid-late 9th Millennium cal BP), with similarities to the Hassuna tradition. It is located on the Piedmont plain adjacent to the foothills of the Zagros Mountains, near modern-day Kirkuk in northern Iraq. A small number of pig remains (although constituting ca. 25% of the assemblage) all appear to be morphologically domestic (208); (171); (174). Specimens AA058 and AA062 were analyzed.

Montaigle, Belgium
Contact: Bea De Cupere
The site of Montaigle is located on a promontory at the confluence of the Molignée and Flavion rivers, about 25 km south of Namur and 8 km to the east of the Meuse river. Below the ruins of a late 13th-16th century castle, human presence has been attested for the Iron Age (around 450 BC), followed by a late Roman level (270 AD to 5th century) which corresponds to the occupation of a small military garrison (209); (210). One domestic specimen (AA279) was analyzed.

Mount Petrino (Mondragone, Caserta), Italy
Contact: Domenico Fulgione, Barbara Wilkens
Mount Petrino (Mondragone, Caserta Italy) site is located on the small terraces of the upper slopes of the South-East side of Monte Petrino, at an altitude of about 190-212

m.a.s.l.. Archaeological excavations started in the 80s, when numerous ceramic fragments were found. A sample used in this study (coded AL1116) was dated 3,600-3,200 BP in the stratigraphic/cultural Bronze Age. It is a lower molar M2 of *Sus scrofa domesticus* from the mandible of a small cooked specimen. Other samples included in this style are of domestic and undetermined status (AL1124, AL1125, AL1126). Other domesticated species found are ox (*Bos taurus*), goat and sheep (*Capra hircus* and *Ovis aries*). Interestingly, this site shows a clear separation between wild and domestic forms.

Mureybet, Syria
Contact: Lionel Gourichon
Tell Mureybet was an archaeological mound situated on the left bank of the Euphrates River, ca. 100 km south from the Syrian-Turkish border, and excavated by J. Cauvin in the early 1970s before the creation of Assad Lake in Syria. The stratigraphy covers a long sequence ranging from the end of the 13th millennium to the 10th millennium BP, i.e. a large part of the neolithization process in the Near East. According to the botanical studies cultivation of morphologically wild cereals was attested since the 12th millennium BP while hunting of large and small game, including a great species diversity of mammals and birds was the main economy until the latest occupations. The two pig specimens (AA355, AA356) analyzed come from the Pre-Pottery Neolithic A (PPNA) levels, dated approximately to the 12th millennium BP (211); (212).

Narva I, Estonia
Contact: Gennady Baryshnikov
Narva I (Riigiküla I in Estonia) is the open site, one of three Neolithic sites (Narva I, II, III) located on the sand dune on the left bank of Narva River, 8-9 km northwards of the city of Narva, in Estonia. Narva I is situated at height of 8.5 m above the river. It is dated by 5th millennium BP (213). Fossils of *Sus scrofa* have been recorded in Narva (layers II and III, early Holocene), including 71 bone fragments (23.1% of all ungulate remains) and in Narva (layer I, middle Holocene), including 140 bone fragments (46.7% of ungulate remains) (214). Collection ZIN 25885 (LG234) from Narva I involves bone material from N.N. Gurina excavation in 1951-1953. It contains 493 bones from 8 adult individuals (calculated by G. Baryshnikov). The sample taken from a humerus (ZIN 25885-65) has been provided for the analysis.

Nivå (Nivaa), Denmark
Contact: Kim Aaris-Sørensen, Peter Rowley-Conwy
Nivå is a multiple Late Kongemose and Early Ertebølle settlement and activity areas on a former east-facing fjord in northeast Zealand, just north of the better-known Vedbæk Fjord. The major settlement of Nivå 10 produced the sites of 4 small huts, as well as 6 inhumation and 3 cremation burials, suggesting the presence of a cemetary like that at Vedbæk. If the economy was similar to Vedbæk, subsistence would have been dominated by small marine fish. No shell middens were present. The sample analyzed (AA014) dates from the Late Mesolithic period.

North Sea, Netherlands
Contact: Dick Mol
The southern North Sea is a rich source of fossil remains dating from the entire Pleistocene (e.g. (215); (216); (217); (218); (219)). Fishermen have been finding

fossils in their nets since at least 1874 (220). In the beginning, localities near the Brown Bank, half-way the Dutch IJmuiden and the English Lowestoft, were recognized as being sources of proliferous finds, but later, many other fossil-rich sites have become known. Smaller vessels that fish within the 12-mile zone of the Netherlands also brought many skeletal remains ashore. These fossils originated from an area north of the mouth of the Westerschelde estuary, offshore from the provinces of Zeeland and South Holland. In the early 1990s these smaller ships started fishing in the so-called Eurogeul, the 28 m deep shipping lane leading toward the Rotterdam harbour. This appears to be the most productive locality nowadays, together with the region south of it. Now we have an enormous wealth of material from the area of the North Sea that has become known as the Southern Bight. The area southwest of the Brown Bank and the surroundings of the locality called "De Stekels" (52°27'05" N. lat. and 03°10'08" E. long.) is well-known for an Early Holocene fauna including remains of humans and wild boar.

The fossil record is dominated by a Last Interglacial Fauna including terrestrial mammal species such as *Stephanorhinus kirchbergensis*, *Elephas antiquus*, and *Hippopotamus incognitus* of which the radiocarbon date is >45,000 BP followed by a Late Pleistocene Fauna with 14C dates ranging between 24,670 BP and 48,400 BP. This terrestrial fauna includes species such as *Ursus sp*., *Castor fiber*, *Crocuta spelaea*, *Panthera leo spelaea*, *Megaloceros giganteus*, *Rangifer tarandus*, *Bison priscus*, *Ovibos moschatus*, *Equus caballus*, *Canis lupus*, *Coelodonta antiquitatis*, *Mammuthus primigenius*, and *Lepus sp*. Remains of an Early Holocene terrestrial fauna has been recorded including the following species: *Castor fiber, Cervus elaphus, Alces alces, Capreolus capreolus, Canis familiaris, Homo sapiens, Lutra lutra, and Sus scrofa*. This Early Holocene fauna with 14C dates in the range from 7,780 to 8,780 BP indicate that the southern bight of the North Sea between the British Islands and the continent of Europe was still dry land and the area was inhabited by humans hunting on, among others, *Sus scrofa* (221); (222). The specimens analyzed are GL208, GL209, GL210, GL211, GL212, GL515, GL516.

Noyen (Haut-des-Nachère), France
Contact: Jean-Denis Vigne
The open-air site of the Haut-des-Nachères, at Noyen-sur-Seine (Seine-et-Marne), is located on the bank of the Seine river, in the center of the Paris Basin, at c. 60 m a.s.l. Besides the rests of a large Middle Neolithic Michelsberg fortified occupation, the excavations conducted between 1983 and 1987 provided five main loci with stratified Mesolithic deposits, totaling nearly 1,000 m² spread across an exposed area of approximately 3 ha (223). These deposits were composed of peat and sandy stratigraphic units accumulated in fluvial channels which were used for rejecting domestic refuses by human settlements which were located on the nearby banks and which are nowadays completely eroded. The excavations provided more than 7,000 animal bones remains referable to both hunting and fishing, worked objects in bone and wood (including a dugout canoe), evidence of wickerwork, human remains as well as a sparse and "atypical" lithic industry. Two topo-chronological systems could be distinguished: the oldest one was radiocarbon dated (wood; N=8) to between 9,190 and 7,970 cal BP (N=6) and attributed to the Middle Mesolithic, and the most recent to the north, dated to between 8,060 and 6,995 cal BP and assigned to the Late/Final Mesolithic with Montbani bladelets. Although they are still incompletely published, these data represent one of the richest and best quality documentation for the Middle

and Late Mesolithic of the Northwest Europe (224). The specimens analyzed (AA357, AA358, AA359, GL431, LG370) date to the Middle Mesolithic.

The rich collection of 7,200 vertebrate remains (5,350 identifiable) also represents a reference collection, remarkable not only for the its state of preservation, but also for the care in which it was collected (large volumes of wet-sieved sediment). During the seemingly year-round Middle Mesolithic occupations, red deer (*Cervus elaphus*) was the main prey species (43% NISP), followed by wild boar (*Sus scrofa*; 27%; used in the present study), aurochs (*Bos primigenius*) and roe deer (*Capreolus capreolus*). Patterns of Late/Final Mesolithic faunal remains depart significantly from those of the Middle Mesolithic as wild boar come to represent 70% of the prey signals. Hunting practices targeted females with their young. Seasonality data is consistent with the results obtained from fish remains (mostly pikes and eels) in the same deposits and indicates a small number of temporally specific hunting episodes most likely situated at the end of the summer (225); (226); (224).

Okolište, Bosnia-Herzegovina
Contact: Norbert Benecke
Okolište is a late Neolithic tell in Central Bosnia (Bosnia-Herzegovina) belonging to Butmir Culture. It was located directly at the river Bosna, which, together with the river Neretva, was connecting the Central Danubian with the Adriatic area. Most of the animal remains found during the excavations of 2002 and 2004 are of domestic origin (82%), which in turn are largely dominated by cattle (227). Out of the only 47 domestic *Sus* remains described, twelve samples (Oko1, Oko2, Oko3, Oko4, Oko12, Oko13, Oko14, Oko15, Oko16, Oko17, Oko18, Oko19) dating to around 5,100-5,000 were analyzed within this study. Five of them (Oko1, Oko3, Oko13, Oko15, Oko16) provided successful PCR amplification of their partial mtDNA.

Oudenburg, Belgium
Contact: Anton Ervynck
The castellum of Oudenburg is a Roman military fortification located on a sand ridge overlooking the Flemish coastal plain (Flanders, Belgium). Its history and food economy are well-known through recent excavations (228); (229). The domestic pig remains used (AA282, AA283, AA284, AA285, AA286) for this study derive from a dark earth layer, deposited (and reworked) during late Roman and early medieval times. Most of the material, however, dates back to the Roman period (2nd to early 5th c. AD).

Oulen (Baume d'Oulen ou d'Oullins), France
Contact: Jean-Denis Vigne, Daniel Helmer
The site of Oulen (Garn, Gard) is mostly composed of a vast cave porch (50 m wide, 15 m high; 220 m a.s.l.), dominating the right bank of the Ardèche River, near the lower Rhône Valley. A thick stratigraphy has been deposited between the Late Mousterian and the Neolithic times. The excavation of the Neolithic deposits has been processed during the eighties (230); (231). They revealed a thick, rich and rather well preserved deposits (fireplaces, pole holes, abundant domestic refuses) dating to the early Neolithic Cardial (layer 6) and Epicardial phases (layer 5), respectively dated to c. 7,300 and 6,900 cal BP, topped by a far less well preserved and rich succession of layers dating to the Chasséan Middle Neolithic and Ferrière Late Neolithic (5th to 4th millennia cal BC; 18 radiocarbon dates on bone collagen, Vigne & Helmer, unpubl.).

These deposits result from the sub-permanent, then seasonal occupations of the porch of the cave by groups of caprine herders (232).

With nearly 4,800 identified specimens, the faunal remains of the Cardial/Epicardial Neolithic deposits of Oulen represents one of the three most important archaeozoological collections in the French Midi for this period. It is highly dominated by domestic sheep and goat (58%), domestic cattle and suids (used in the present study) representing 16% each. The unpublished osteometric study demonstrated that most of the suids were small sized (thus attributed to domestic ones), some larger individuals being considered as hunted wild boar (Vigne & Helmer unpubl.). Two specimens were analyzed (AA287, AA288) date to the Early Neolithic.

Ovčarovo-gorata, Bulgaria
Contact: Norbert Benecke
Ovčarovo-gorata is an Early Neolithic *tell* in Northeastern Bulgaria close to Târgoviște belonging to Ovčarovo-Samovodene-Culture (paralleled with Karanovo II). It was part of a probably densely populated settlement chamber continuously inhabited from the Neolithic to Copper Age (62). The *tell* itself dates to between 7,700-7,500 cal BP and can be paralleled with Karanovo II. The faunal remains represent typical food deposits of a settlement with only around 6% of wild animals. Cattle are dominating the domestic faunal record (72%) followed by sheep and goat (20%) and pigs (8%). One (Ovc1) of the two (Ovc12) domestic pig samples analyzed here provided successful PCR amplification of its partial mtDNA, and was typed as Y1.

Palaikastro, Greece
Contact: Anastasia Papathanasiou, Mike Richards
Palaikastro is located on the coast of east Crete (about 145 km from Herakleion via Agios Nikolaous). It is the site of a large and wealthy Minoan town first excavated by British archaeologists Bosanquet and Dawkins (1902-1906). From the 1960s to the present, work has continued under the auspices of the British School at Athens. The total area uncovered is about one-third of a square kilometre but remote sensing indicates there is a great deal more to be found in the south and east, including the possibility of a palace. A multitude of important artefacts have been found at the site, including Linear A tablets and an exquisite ivory sculpture of a bull leaper. It was a wealthy town with fine houses and a drainage system. It was inhabited from the middle of the third millennium until post-3,450 BP, the time of the destruction of most Minoan settlements, although with its living standards at a much reduced level. Three specimens (GL1078, GL1079, GL1080) that date to the Bronze Age were analyzed.

Palegawra, Iraq
Contact: Max Price
Palegawra Cave is located in the Zagros foothills, just to the east of Jarmo. The site was occupied in the Epipaleolithic, with material culture attributed to the Zarzian tradition (Ca. 16,000?-11,500? cal BP). All the suids found were hunted wild boar (233). One wild boar specimen (AA059) from the Zarzian culture was analyzed, and dates to approximately 12,200-9,600 BP.

Pendimoun, France

Contact: Lionel Gourichon
The rock-shelter of Pendimoun is located on the French Riviera about 700 m a.s.l. and 4 km from the actual Mediterranean seashore. This place was regularly used by human groups from the Mesolithic (Sauveterrian) to the 5th millennium BP and is one of the earliest Neolithic settlements in Western Mediterranean (early 8th millennium BP, Impressa Ware culture (234). Suid remains are relatively scarce along the Neolithic sequence and are generally highly fragmented (122). Among the 3 specimens analyzed, all coming from the late phase of the Early Neolithic (Square Mouth Culture culture, ca. 7,200-6,950 BP), only one (LG215) gave genetic data which indicate a ANC-Italy haplogroup pig.

Pietrele, Romania
Contact: Norbert Benecke
The settlement mound of Măgura Gorgana lays close to the village Pietrele in Southern Romania next to the Danube river. While the settlement surrounding the *tell* was inhabited already during the Late Neolithic Boian culture, the tell itself revealed finds from Copper Age Gumelniţa Culture (235). During the Copper Age phase, domestic and wild pigs dominate the faunal record (around 75% and 56%, respectively). The samples analyzed here stem exclusively from the Copper Age *tell* itself and date to around 6,450-6,250 cal BP. Eight of ten samples were successfully analyzed for a partial mtDNA fragment, four of them represent wild boar (Pie1, Pie2, Pie3, Pie4), and the others domestic pig (Pie12, Pie13, Pie14, Pie15, Pie16).

Pločnik, Serbia
Contact: Jelena Bulatović, David Orton
Pločnik is very important site located on the left bank of the Toplica river in south Serbia. Based on its very abundant and specific ceramic finds, the late phase of the Late Neolithic Vinča culture was named after Pločnik (236). Besides numerous copper artefacts, traces of early metallurgical activity are found across the deposit of the settlement, as well as a unique find of in situ metallurgical workshop for casting and/or repair of metal tools in the central Balkans (237). This settlement with c. 3.6 m thick cultural layer (238) was occupied for c. 600 years, between 7,200-6,650 cal BP (136); (237). In total, 18 specimens of domestic pigs were analyzed (AA204, AA206, AA209, AA210, AA211, AA212, AA213, AA215, AA216, AA217, AA218, AA219, AA220, AA221, AA222, AA223, AA224, AA225).

Popești, Romania
Contact: Valentin Dumitrascu
The site at Popești is located at a distance of approximately 25 km southwest of Bucharest, on the right bank of the Argeş River (Giurgiu County). The complex stratigraphy ranges from Bronze Age to La Tène period (239). The late Bronze levels and are not separated by a sterile layer from the ones above them: the ceramic layer of the Zimnicea-Plovdiv type (about 14th or 13th centuries BC) and the Fundeni-Govora Ceramic Layer dated by radiocarbon before 3,400 BP. Two samples from domestic pigs were analyzed from the last layer. An Early Hallstatt layer (dated back to 10th century BC) overlaps a layer of building debris, pre-Basarabi and Novaci pottery (about 9th century BC). The Middle Hallstatt consists mainly of sporadic architectural remains and Basarabi type fragmentary ceramics (about 8th century BC). A wild boar mandible was analyzed from this context. The La Tène is represented by 5 successive levels, dated between approximately 2,150 BP and year 2 (or 4) AD. The settlement is

urban and is considered to be a regional center of power. A domestic pig mandible (AA083) was analyzed from a level dated around 2,100 BP (240). Five specimens were analyzed (AA079, AA080, AA082, AA083, GL150).

Pupićina Cave, Croatia
Contact: Preston Miracle
Pupićina Cave is situated at 220 m a.s.l. in the lower reaches of Vela Draga canyon in north-eastern Istria, Croatia. The cave is funnel-shaped; the southeast-facing entrance is about 20 m wide and 8 m high. Thick deposits revealed deep stratigraphic sequence with archaeological layers spanning from the late glacial period to the late Roman period (241). Eleven specimens (AA456, AA457, AA458, AA459, AA471, AA472, AA473, AA474, AA475, GL726, GL980) were analyzed.

Racot 18, Poland
Contact: Arkadiusz Marciniak
The Late Lengyel settlement in the western part of the Polish lowlands south of Poznań excavated in the years 1984-1987. The excavated part revealed 14 trapezoidal longhouses accompanied by numerous external and internal pits and one burial. The site extends over an area of ca. 3 ha with an estimated number of 40-48 to 48-56 longhouses (242). Three domestic specimens analyzed (LG711, LG712, LG721) date to the Neolithic.

Regensburg-Harting, Germany
Contact: Joris Peters
The remains collected at Regensburg-Harting originate from a Roman villa rustica that was built towards the end of the 1st century AD and abandoned in the 4th century AD. Analysis of the faunal remains has been concluded but the faunal results still await publication (243). Five specimens were analyzed, of which four are domestic pigs (LG164, LG165, LG589, LG590) and one is unknown (LG588).

Rosstal, Germany
Contact: Joris Peters
Erected in Carolingian times, Rosstal castle was inhabited from the early 9th until the 11th century AD. Its fauna is largely dominated by the remains of pigs. Compared to contemporaneous *Sus* populations elsewhere in Bavaria, the pigs from Rosstal are large-sized animals, with shoulder heights ranging between ca. 70 and 94 cm. Pig husbandry likely benefited from the extensive deciduous forests occurring in the region. Provided silvopasturing was practiced by the site inhabitants, one explanation for the large size of the pigs could be admixture with wild boar (244). Six early Medieval suids were analyzed (LG646, LG647, LG649, LG650, LG651, LG652).

Roucadour, France
Contact: Josephine Lesur, Anne Tresset, Jean-Denis Vigne
Roucadour (Lot, France) is a site located south-west of the Central Massif, in a sinkhole at 346 m a.s.l.. It has been occupied during a long period, from the Neolithic up to the Iron Age. The suid bones included in this study come from a Middle Neolithic horizon belonging to the Chasséen culture and dated between the end of the 7th millennium and the beginning of the 6th millennium cal BP. The faunal assemblage is strongly dominated by wild species, which is somehow surprising in a site from the

Chasséen culture. Suid remains account for ca. 26% of the identified mammal remains (245). Three wild boar (GL321, GL470, GL485) specimens were analyzed.

Rottenburg-Siebenlinden, Germany
Contact: Elisabeth Stephan
Rottenburg-Siebenlinden is a Mesolithic open-air site on the banks of the river Neckar about 50 km south of Stuttgart in southwest Germany. Excavated extensively between 1990 and 2004 a site of about 480 m^2 was investigated and several thousand lithic artefacts as well as wild animal remains were recovered from four different Mesolithic layers. Layer II dates to the Late Mesolithic (c. 8,500-8,100 cal BP, Atlantic period), and layer IIIo, III, and IV to the Middle Mesolithic (III: Beuronien C 9,400-9,100 cal BP, Late Boreal period; IV: Beuronien B 10,100-9,700 cal BP, Early Boreal period). One wild boar specimen (LG166) from layer IV was analyzed.

Sagalassos, Turkey
Contact: Bea De Cupere
Sagalassos is an urban site in southwestern Turkey near the village of Ağlasun (Burdur province), in a region known as Pisidia in ancient times. The town was built on a south-facing hill slope in the western portion of the Taurus mountain range and mainly inhabited during the Roman-Early Byzantine period. The nearby fortified village of Düzen Tepe dates to the Classical/Hellenistic period (2,500-2,200 BP) (246). Specimens dating to the Imperial period to the Early Byzantine were analyzed (AA030, AA031, AA032, AA033, AA034, AA035, AA036, AA037, AA038).

Samovodene, Bulgaria
Contact: Norbert Benecke
Samovodene is a Neolithic *tell* in Bulgaria North of Veliko Tarnovo at the river Jantra. The earliest settlement phase is paralleled with Early Neolithic Karanovo II (7,800-7,500 cal BP) and belongs to Ovčarovo-Samovodene-Culture (187). This early phase represented by one wild boar and one domesticated pig sample in the present study. We also analyzed two wild boar from the early middle Neolithic period (around 7,500 cal BP, paralleled with Karanovo II/III) and three late Neolithic wild boar (paralleled with Karanovo IV, 7,300-6,900 cal BP). Analyses of the faunal remains revealed a focus on cattle farming (more than 80%) and a only minor role of domestic pigs (63). Four (Sam5, Sam9, Sam10, Sam18) out of the seven samples (Sam6, Sam19, Sam20) provided successful amplification of the partial mtDNA fragment, including the domestic pig (Sam5).

Šandalja II
Contact: Preston Miracle
Šandalja II (44° 52' 57" N, 13° 53' 48" E) is a “fossil” cave, located at the southern tip of Istrian Peninsula in the northern part of the eastern Adriatic coast at an absolute elevation of 72 m/a.s.l. (247); (248). The deposits were divided into layers A – i. Layer A was of the Holocene age, layer Bg has dates from both the Early Holocene and the Late Glacial interstadial, while the rest of the layers were deposited during the Late Pleistocene (249). One specimen (LG335_R1) was analyzed.

Santa Maria in Selva, Italy
Contact: Domenico Fulgione

The site of Santa Maria in Selva is located near the city of Macerata, in the center of Italy, on the top of a hill. In it were found various findings of domestic animals (bred) and various objects including vases with a spool handle and with curled margins in the guise of cornets, a truncated cone shell with a wide mouthpiece adorned entirely by triangular incisions. fragments of copper confirm the dating of this facies to the final Neolithic. In the village were also used domestic cereals that were also found in the lining of huts made of clay. the suids are attributable to domesticated forms (250). One wild boar specimen (AA629) dating to the Late Neolithic was analyzed.

Schela Cladovei, Romania
Contact: Clive Bonsall
Schela Cladovei (44.62612° N, 22.60591° E) is a large, open-air settlement in the Iron Gates section of the Danube Valley in southwest Romania. The site occupies an Early Holocene terrace adjacent to the Danube, ~7 km downriver from the Iron Gates I dam. Discovered in 1964, early investigations (1965-1968, 1982-1991) were undertaken by Vasile Boroneanţ. Since 1992 the excavations have been conducted mainly as a joint Romanian-British research project, co-directed by V. Boroneanţ (later A. Boroneanţ) and C. Bonsall. In the excavated areas of the site the archaeological remains relate mainly to Late Mesolithic (~9,200-8,300 cal BP) and Early Neolithic (~8,000-7,600 cal BP) occupations (80), but there is sporadic evidence of later (Bronze Age, Iron Age and Medieval) activity. The 5 samples (AA402, AA403, AA404, AA405, AA406) included in this paper were excavated from Area VI in 1992-1996 by V. Boroneanţ and C. Bonsall, and they were submitted for aDNA analysis by Alexandru Dinu without the details of their archaeological contexts, which makes chronological placements uncertain for those samples without direct AMS ^{14}C dates.Of the two directly dated samples, AA406 (2-sigma calibrated range 9,247-8,649 cal BP) is Late Mesolithic and AA402 (2-sigma calibrated range 7,756-7,505 cal BP) is Early Neolithic (251)

Shan-Koba (Abri Šan-Koba), Ukraine
Contact: Norbert Benecke, Gennady Baryshnikov
Shan-Koba shelter is located in south-western, mountainous, Crimea, in the Baidar valley. Excavations by G.A. Bonch-Osmolovsky in 1928 and by S.N. Bibikov in 1935-1936 revealed 6 cultural layers. It was inhabited during Mesolithic and Neolithic times. Layers 1a is dated by Neolithic, layers 2-4 – Mesolithic, layers 5 and 6 – Final Paleolithic (252). Archaeozoological investigations showed that the faunal remains mostly constitute food waste of human hunters (253). Two Mesolithic (around 7,900-9,500 cal BP - San3, San6) and three Neolithic (around 7,300-7,700 cal BP - San7, San8, San9) of the nine wild boar (San1, San2, San3, San4, San5, San6, San7, San8, San9) in this study provided successful PCR amplification of their partial mtDNA. *Sus scrofa* is represented by 101 bone fragments belonging to 8 individuals, from layers 4-6 (San2), and by 482 remains belonging to 10 individuals, from layers 2-3 (San3, San6) (254). Bone material recovered by Bibikov excavation is kept in Zoological Institute, Russian Academy of Sciences in St. Petersburg (ZIN).

Siniarzewo 1, Poland
Contact: Arkadiusz Marciniak
Siniarzewo 1 is a Late Lengyel settlement in Kujavia in the Polish lowlands. The site was discovered in the 1990s during the rescue excavation project preceding the construction of the Yamal pipeline from Siberia to western Europe. The discovered

remains comprised a trapezoidal longhouse with accompanying pits in addition to a pig burial in one of the pits. Remains of Bronze Age settlement were also revealed. Out of the specimens that provided successful amplification of their DNA, two of them represented Late Lengyel (AA140, AA141) while remaining are Bronze Age (AA144, AA145, LG507, LG508, LG513, LG514) (255).

Sirkeli Höyük, Turkey
Contact: Joris Peters
Sirkeli Höyük is located in Cilicia, the south coastal region near Adana in present-day Turkey. The site was inhabited from the Chalcolithic period till the Iron Age (6,000-2,500 BP). Because of the semi-arid climatic conditions prevailing in the region, however, pig husbandry was not that important in the site's economy, but relative frequencies increase throughout site occupation. The game species most commonly hunted by the site inhabitants was Mesopotamian fallow deer, but from time to time wild boar was killed as well (256). Four Iron Age specimens (LG522, LG524, LG527, LG529) were analyzed, of which three are domestic.

Sofia-Slatina, Bulgaria
Contact: Norbert Benecke
Sofia-Slatina is a Neolithic settlement south of the former village of Slatina, which is now a district of the Bulgarian capital of Sofia. Excavations started in 1985 as a rescue excavation and revealed finds dating back up to the Early Neolithic.The four domestic pigs analyzed here come from horizons belonging to Kremikovci Culture (paralleled with Karanovo II) and date to 7,750-7,600 cal BP. The faunal spectrum of the site is dominated by domestics with pigs playing only a minor role (below 10%) (63). Three of the four samples (Sof1, Sof2, Sof3, Sof4) were successfully analyzed for a partial mtDNA fragment.

Soroca III near Soroka, Moldova
Contact: Norbert Benecke
Soroca III is a Mesolithic/Neolithic settlement on the left bank of the Dnestr river in Moldova, dating to 8th–7th millennium BP (257). One Neolithic wild boar specimen (GL126) was analyzed.

Shpan-Koba near Lesnoe Kipčak (Crimea), Ukraine
Contact: Alexandr Yanevich, Norbert Benecke
This site is a rockshelter at a distance 15 km east from Simferopol in the mountainous part of the Crimean peninsula at an altitude of 800 m above sea level, on the south-eastern edge of the mountain range Dolgorukovskaya Yayla. It was discovered in 1925 by A.S. Trusova. Excavations by A.A.Yanevich in 1988-1989 (258) revealed there 6 cultural layers with occupation during the Mesolithic and Neolithic periods (Preboreal-Atlantic). There are 12 14C dates (c. 12,500-9,000 cal BP) (259). Cultural layers are left during seasonal visits of hunting groups in the warm season. Two wild boar specimens (GL128, GL536) were analyzed.

Spazzavento, Italy
Contact: Domenico Lo Vetro, Lucia Sarti
The Neolithic Spazzavento area is part of the larger Spazzavento-Neto-Via Leopardi Settlement Unit in the Sesto Fiorentino area of Italy (260). The site is part of a broad settlement unit which is formed by excavation areas: Neto-via Verga, Neto di Bolasse

and Spazzavento. The unit, positioned in the west of Sesto Fiorentino territory, is located on a sort of morphological top formed by depositional accumulations of two alluvial fans. The stratigraphic sequence as a whole covers a wide chronological range which indicates that the area was occupied from the 5th millennium until the Roman period (261); (262). 3 Neolithic wild boar samples (AA635-AA637) could not be used in the analysis because the haplogroup tests failed.

Staraya Ladoga, Russia
Contact: Gennady Baryshnikov
Staraya Ladoga, is an ancient city in the lower stream of Volkhov River near Ladoga Lake, Russia. Bone material has been collected there during N. Repnikov excavation in 1909-1913, from Zemlianoe gorodishche, which is characterized by several temporal levels dated by the second half of 8th century to 10th century (263). Collection of *Sus scrofa domestica* (ZIN 18757) comprises 394 bone remains belonging to 13 adult individuals (calculated by G. Baryshnikov). The sample taken from tibia ZIN 18757-105 (LG239) has been given for the analysis.

Stuttgart-Mühlhausen, Germany
Contact: Elisabeth Stephan
Stuttgart-Mühlhausen 'Viesenhäuser Hof' is located on a loess ridge in the Neckar river basin in the north of Stuttgart in southwest Germany. The excavations between 1931 and 1993 have revealed remains of the oldest, older and younger Linearbandkeramik as well as the Middle Neolithic, the Middle and Late Bronze Age, and the Early Iron Age (Late Hallstatt/Early Latène). During the LBK a settlement and large cemeteries existed. Two specimens of domestic pigs from the LBK settlement were analyzed (LG103, LG167).

Suberde Höyük, Turkey
Contact: Benjamin Arbuckle
Suberde Höyük is located in the intermontaine Sugla basin in southwest Turkey (264) on a limestone ridge along the margin of Lake Sugla at an elevation of 1,070 m. Suberde was first recorded in a survey by R. Solecki (265) and was excavated for two seasons in 1964 and 1965 by Jacques Bordaz as part of the Beysehir-Sugla Project. Bordaz described four stratigraphic levels at the site with levels I-III consisting of cultural remains, while level IV consisted of sterile sediments. Levels II and III consist of the remains of plastered floors and rectilinear mudbrick architecture including storage and bench features. Pottery is rare or absent in these levels which are thought to represent the remains of a small, 0.5 ha, village settlement dating to the latest part of the Aceramic Neolithic Pottery (264). Level I represents a mixed surface layer consisting of Roman to Islamic period burials, and a small number of Neolithic, Chalcolithic, and Bronze Age sherds. The prehistoric levels of the site are radiocarbon dated to 9,500-9,000 BP (266). Ten *Sus* specimens from these prehistoric levels were submitted for ancient DNA analysis, and 9 were analyzed (AA390, AA391, AA392, AA393, AA394, AA395, AA396, AA397, AA398).

The faunal assemblage from Suberde is dominated by the remains of morphologically wild, but probably managed, sheep and goats followed by pigs which represent 13% of the mammalian remains (267). Wild cattle and red deer are present in significant but relatively small numbers. The pig remains from Suberde are interpreted as representing Anatolian wild boar. Recent archaeozoological analysis of the *Sus*

remains has shown that the Suberde pigs are comparable in postcranial breadth and depth measurements (and therefore body size) to prehistoric wild boar documented at Göbekli Tepe, Erbaba Höyük, Çatalhöyük, and Asıklı Höyük (268). Only a small portion of the original Suberde faunal assemblage remains following its export from Turkey by Dexter Perkins in the 1960s (see (266)). The remaining assemblage is curated in the Zooarchaeology Laboratory, Department of Anthropology, University of North Carolina at Chapel Hill.

Surb nahatak (Sevcar), Armenia
Contact: Ninna Manaseryan
Surb nahatak (Sevcar) is a fortress is located on the southern outskirts of the village of Sevkar (Ijevan district). It dates from the 2,700-2,500 BP. The excavations were performed by archaeologist S. Yesayan (176). One domestic specimen (AA118) was analyzed.

Swinegate, England
Contact: Terry O'Connor
The site in Swinegate, York, is located at the heart of the medieval city, in the southernmost quarter of the Roman fortress. The medieval occupation consisted in the main of small properties clustered around St Benet's churchyard, now extinct. The specimens reported here derive from mid-14th century activity (269). Two specimens (AA303 and AA304) were analyzed.

Tel Bet Shean, Israel
Contact: Liora Kolska Horwitz
Tel Bet Shean is favorably situated in the central Jordan Valley, at the intersection of trade routes running north-east from the coast into the hinterland. The main occupation levels date to the Late Bronze Age II through to the end of the Iron Age (ca. late 3,600-3,400 BP to 2,700-2,500 BP), although earlier remains dating to the Early Bronze Age have been found at the site as well as fragmentary evidence for occupation in the Roman, Byzantine and Islamic periods (270). Pigs occur in low frequencies throughout the sequence. The two specimens (AA041, AA043) examined in this study derive from the Late Bronze Age II strata (ca. 3,550-3,400 BP) and represent domestic swine (271).

Tel es-Safi/Gath, Israel
Contact: Liora Kolska Horwitz
Tel es-Safi/Gath is located ca. 53 km east of the city of Ashkelon in the central coastal plain of Israel. Current excavations at the *tell* have demonstrated that it was occupied from the Early Bronze Age to the Ottoman period (272). Human activity at the site peaked during the Iron Age (3,200-2,700 BP), when it was one of the main Philistine cities. The domestic pig specimen (AA043) examined here, derives from these layers. Notably, the Philistine occupation was accompanied by the introduction of pigs from the Aegean, who were characterized by European haplotypes and subsequently altered the genotypic profile of local swine populations (273). Two specimens (AA041, AA043) were analyzed.

Tel Miqne-Ekron, Israel
Contact: Liora Kolska Horwitz

The *tell* is located on the Israeli coastal plain (Shephelah) ca. 35 km south-west of Jerusalem. It sits on trade routes going north-east from the coast into the hinterland. The site was excavated under the direction of Trude Dothan (Hebrew University) and Seymour Gitin (274) beginning in 1981 through 1996 (274); (275). Tel Miqne-Ekron has been identified with biblical Ekron, one of the five Philistine cities that existed on the central Israeli coastal plain in the Late Bronze Age II through to the end of the Iron Age (ca. 3,600-3,400 BP to 2,700-2,500 BP). Scanty earlier remains dating to the Chalcolithic and Early Bronze Age have been found at the site as well as fragmentary evidence for occupation in the Roman, Byzantine and Islamic periods.
At its zenith in the Iron Age, site size is estimated as 20 ha, with a lower (elite) area of 40-65 acres and an upper area, ca. 10 acres. The site experienced a complex history of destruction, abandonment and re-occupation. Architectural finds include a fortification system (Iron Age I), residential and industrial areas, and off-site agricultural installations. Philistine material culture attests to Aegean and Cypriot affinities, as well as interactions between the Philistines, Israelites, Phoenicians, Assyrians, and Egyptians.

Faunal assemblages from different parts of the *tell* have been studied by the late Brian Hesse, Justin Lev-Tov and Edward Maher (276); (277); (278); (279). In almost all periods, domestic caprines formed the mainstay of the economy and were closely followed by cattle and, at the height of Philistine rule, also by pigs whose frequency drops off in the late Iron Age. Remains of domestic equids, dogs and wild animals are negligible in all periods but are often found in ritual contexts (278). Sheep-goat proportions change over time, with a predominance of sheep in the late Iron Age indicative of wool-production. Mortality patterns also change and are interpreted by Lev-Tov (277) as evidence for a shift from a local, household-oriented caprine production system focused on production for meat and secondary products, to a market-oriented system geared primarily toward secondary products in the late Iron Age. Three specimens (AA276, AA277, AA278) dating to the Iron Age were analyzed.

Tell Aqab, Syria
Contact: László Bartosiewicz
The two pig samples (BLT004, BLT005) from Tell Aqab, Syria presumably come from Middle Halaf Period layers. They included a mandibula fragment and a proximal radius fragment. This is a *tell* site in Jezirah Province, NE Syria. A single Middle Halaf sample has been 14C dated (Aqab 212, AA-30498) to 7,579–7,350 BP (280).

Tell Brak, Syria
Contact: Roger Matthews
Tell Brak is located in the Upper Khabur region of northeast Syria, approximately halfway between the modern towns of Hassake and Qamishli. Tell Brak, ancient Nagar, is one of the most important and long-lived archaeological sites of Upper Mesopotamia, with occupation spanning ca 8,000 to 3,200 BP. Major features of its history including evidence for early urbanisation at ca. 5,500 BP, a multi-phase Eye Temple containing thousands of stone ‘eye idols’, major administrative and religious buildings of the 5th millennium BP, and a Mitanni palace on top of the mound at ca. 3,500 BP. Archaeological evidence indicates that the site was a major focus of trade, administration and trans-regional engagement across Upper Mesopotamia and well beyond, taking advantage of its prime location at a transection of east-west and north-

south trade routes. Nine suid specimens (AA020, AA021, AA023, AA093, AA094, AA095, AA096, AA097, AA098) were analyzed.

Tell-Afis Saraqeb, Syria
Contact: Domenico Fulgione, Barbara Wilkens
Tell-Afis Saraqeb, Syria is an archaeological site in the district of Idlib. Excavations of the massive city wall in the lower town and a large sacred building of the *in antis* type on the acropolis, prove that this site was flourishing between the second and third Iron Age. The sample from this site (piece of skull, coded AL613 *domesticus*) was excavated in 2000-2001, in an area that shed more light on the first Iron Age, by discovering many other hunting species, besides wild boar, such as roe deer, gazelle, hare, fox and leopard. During this excavation, also domestic fauna was found, such as sheep, goat, cattle and, even though less frequent, pig. The latter species is less common in the Bronze Age.

Thebes, Greece
Contact: Vassilis Aravantinos, Efrossini Vika
Thebes is found in the region of Boeotia, in the centre of mainland Greece. The region consists of two extensive plains crossed by rivers and surrounded by mountains. Large, freshwater lakes and many natural springs were present in the area. Natural geomorphology and climate were not significantly different in ancient times. The alluvial soils were fertile, suitable for crops and livestock, thus the area was beneficial for human settlement. Several important Bronze Age settlements were located in Boeotia, culminating in the emergence of a Mycenaean palatial building in the Late Bronze Age.

The samples come from the same site and represent the two phases of its use. The site is an Early Helladic building of the characteristic for the time ellipsoidal architectural form, that was destroyed for reasons unknown to research so far. The debris together with a mass burial that was placed over the ruins, was covered under a mud brick tumulus and left to abandonment, until the next period, the Middle Helladic, when it was used as a burial ground. The study of the ceramic assemblage from the site reveals the co-existence of a local ceramic tradition together with shapes of the “Anatolianising” type (Lefkandi I group), with both categories being produced locally (281); (282); (283). One Early Bronze Age specimen (AA370) and one Middle Bronze Age specimen (AA371) was analyzed.

Tmbatir (Achajur), Armenia
Contact: Ninna Manaseryan
Tmbatir (Achajur) is located on the North West boundary of villages Achajur (Ijevan district). It dates from the 2,600-2,4000 BP. The excavations were performed by archaeologist S. Yesayan (176). One Iron Age specimen (AA117) was analyzed.

Tol e Bashi, Iran
Contact: Marjan Mashkour
Tol e Bashi (Iran) is a Late Neolithic in the Province of Fars in Southwest of Iran. The site was excavated by Kamyar Abdi, Susan Pollock and Reinhard Bernbeck. Archaeozoological studies were performed by M. Mashkour and Salvador Bailon (284). Tol e Bashi has a highly pastoral economy. The pig remains are very scarce

in the assemblage and do not seem to derive from the domestic stock. One specimen from possibly Neolithic contexts (AA025) was analyzed.

Trelleborg, Denmark
Contact: Kim Aaris-Sørensen, Peter Rowley-Conwy
Trelleborg is a Viking-period geometric ring fort on the island of Sjælland. The military fortress was constructed using timbers felled in AD 980, probably under the orders of King Harold Bluetooth. It is a causewayed camp situated on a plateau of morainic clay projecting between the rivers Tude Å and Vårby Å in southeastern Zealand. Its position on a peninsula between the two great rivers was probably intended to dominate passage through the Great Belt. It is 137 m in diameter and had four entrances on the cardinal points. Transverse roads connecting opposing entrances divided the interior into four quadrants, each of which contained four major halls arranged in a square. The 16 halls would have provided accommodation for over 1,000 people. 125 pits were found during excavation of the Viking fortress in 1934–42, some of which were later identified as ditches distributed in two parallel rows (285). The various features contained finds from all periods of the Middle Neolithic Funnel-Beaker Culture (286); (47). Two domestic pig specimens (AA016, GL306) were analyzed.

Trou Al'Wesse, Belgium
Contact: John Stewart
Trou Al'Wesse is a cave site located in the valley of the Hoyoux river, a tributary of the Meuse, adjacent to the Belgian Ardennes. The slope deposits on the terrace immediately outside the cave entrance have been excavated revealing several facies of C14-dated Mesolithic (Couches 4b-α, 4b-β, 4b-γ, 5a), transitional Mesolithic/Neolithic (LaH), and Neolithic (Couche 4a) occupations. Seven samples were analyzed from layers 4b-α (1 sample dated to ca. 10,000 BP), 4a (5 samples dated to ca. 5,500 BP), and 2 (1 sample undated) (287).

The following were successful:
TAW 2004 G12 piece 56 (AA128) – Comes from Couche 4a
TAW 2004 G12 piece 48 (AA129) – Comes from Couche 4a
TAW 03 I9 piece 29 (AA131) – Comes from Couche 2
TAW 2004 H7 piece 33 (AA126) – Comes from Couche 4a

Tsakaektsy, Armenia
Contact: Ninna Manaseryan
Tsakaektsy is a rural settlement of the XI-XIY centuries on the ravine of the Agstev river. It is a part of the Ijevan-Taush historical-ethnographic zone of Armenia. The settlement is located 5 km to the east of the village of Sari Glukh (15 km east of the city Ijevan). The area of the settlement is 900 m^2. The excavations are made by H. Yesayan (the son of S. Yesayan)

Six species of animals have been identified there from osteological materials excavated at Tsakaektsy. The results of the analysis returned more than 250 identifiable bone fragments. The fragments show that 97% of them belong to domestic animals (cattle, sheep, pig, and horse). The wild fauna are represented by the roe deer and the turtle. One suid sample (AA114) was analyzed.

Twann-Bahnhof, Switzerland
Contact: Jörg Schibler
Twann-Bahnhof is a Neolithic lakeshore site situated at the northern shore of lake Bienne. The site was excavated between 1974 and 1976. An area of 160 m of 14.5 m of the site had to be excavated because of the construction of a small highway. Within this area a rich stratigraphy of 13 settlement layers was found. 10 settlement layers belong to the Cortaillod Culture, these layers are dated between 5,838 BP and 5,532 BP by dendrochronology. Three settlement layers belong to the Horgen Culture and are dated between 5,405 BP and 5,072 BP. The used sample bone belongs to the youngest layer of the Horgen Culture that is dated to 5,093-5,072 BP. In total 203,743 animal bones were identified from the Cortaillod layers (288); (289); (290). Only 8,085 animal bones were identified from the Horgen layers (291). One Neolithic domestic specimen (LG691) was analyzed. The bones were provided by Archäologischer Dienst des Kantons Bern for this study.

Uivar, Romania
Contact: Wolfram Schier
Gomila is a late Neolithic to early Eneolithic tell close to the village of Uivar, around 40 km southwest of Timişoara in Southwest Romania. It has been surveyed and excavated 1998-2009 by a Romanian-German team and yielded finds from mostly middle to late Neolithic Szakálhát and Vinča (phase C) and early Copper Age Tiszapolgár Culture (292). While domesticated animals were dominating the faunal record of all Vinča phases, especially the number of cattle remains decreased until the final C2 phase from around 70% to 50%. Increased hunting activities and focus on the small domestics pigs, sheep and goat point towards climatic changes at the end of the Neolithic (293). Two wild (Uiv9, Uiv11) and one domestic (Uiv10) *Sus* remains were successfully analyzed for a partial mtDNA fragment. They were excavated in 2008 and come from middle Neolithic Szakálhát / Vinča phase B, more specifically from building phases 5a (7,205-7,090 cal BP (95%), (292), and 4a/3d (7,090-6,990 cal BP (95%) (292)). The third pig sample, belonging to the same building phase 4a/3d, was dated directly (KIA-42156: 6,560 ± 35 BP). This date, attributable to the later Early Neolithic, suggests a rediposited older bone, although no Early Neolithic (Starčevo) settlement could be identified at Uivar.

Ulucak Höyük, Turkey
Contact: Özlem Çevik Canan Çakırlar
Ulucak Höyük is favorably situated along one of the main arteries between the Aegean coast and inland Anatolia in the Izmir province. Well-preserved Neolithic deposits in Levels IV and V represent a material culture akin both with the Lake District and the Greek Neolithic (294), whereas Level VI is unique in the region with pre-pottery deposits on red-plastered floors and material culture with direct links to sites in Syria, Jordan and SE Turkey (295). Pigs in these layers dating to 8,800-8,500 cal BP are morphologically domestic (295); (296). 17 pig specimens that were associated with levels IV and V date from 8,400-7,900 BP (296). Five of these samples, all identified as domestic pigs (AA327, Ulu1, Ulu48, AL1102, AL1103), were successfully analyzed previously (161).

Umm Qseir, Syria
Contact: Melinda Zeder

Umm Qseir is a small settlement on the middle Khabur Basin. Halafian (8th millennium BP) levels at the site, dating to about 7,900-7,000 BP, represent a year-round settlement occupied by a small number of families engaged in mixed agro-pastoral pursuits that included management of domestic sheep, goats, and pigs, supplemented by wild game (297). A later 6th millennium BP Uruk period, occupation is more ephemeral and may represent the encampment of both local and non-local people possibly from southern Mesopotamia. Two specimens (AA049, AA050) from Uruk age deposits were analyzed.

Undir Junkarinsfløtti, Faroe Islands
Contact: Mike Church
The 28 pig bones were recovered from a series of midden and occupation layers, sampled from the coastal erosion section cutting through the extensive farm-mound of Undir Junkarinsfløtti (298). 3 main phases have been identified at the site: 1) UJF1 represents the basal midden layers which contains material dating from the initial Viking landnám in the Faroes, as well as later material from 11th and 12th centuries cal AD that were mixed in with these basal layers (14C dated to 880-1,220 cal AD); 2) two later phases (UJF2 & UJF3) were 14C dated to 1,010-1,220 cal AD. Unusually in North Atlantic zooarchaeological assemblages, pig bones were recovered in significant numbers from all 3 phases, and the pigs were kept both on the settlement and the wider outfield (299); (300). All 37 (AA411, AA412, AA413, AA414, AA415, AA416, AA417, AA418, AA432, AA433, AA434, AA435, AA436, AA437, AA438, AA439, AA440, AA441, AA442, AA443, AA444, AA445, AA446, AA447, AA448, AA449, AA450, AA451, SF010, SF011, SF012, SF013, SF014, SF015, SF016, SF017, SF028) specimens analyzed are of domestic pigs.

Untervaz-Haselbodensenke, Switzerland
Contact: Jörg Schibler
Untervaz-Haselbodensenke is a Neolithic dryland site that was excavated 2001 by the archaeological service of canton Grison (301). Three different Neolithic settlement layers were found. The sample bone belongs to the youngest settlement that is dated typological (by ceramic pot shred) to the Corded Ware Culture (4,700-4,500 BP). The site is situated near the border between the canton Grison and St. Gallen in the region of the upper Rhine valley. The animal bones of the site were identified by S. Braschler. Only 167 bone fragment out of a total of 974 could be identified because the bones were highly fragmented. Four specimens (AA075, AA076, AA077, ASPig15), both wild and domestic, were analyzed.

Vărăşti, Romania
Contact: Adrian Bălășescu
Vărăşti is located on the small island of Grădiştea Ulmilor (nowadays disappeared) on Lake Boian, Vărăşti commune, Călăraşi County. The faunal remains come from a Boian culture context, the Vidra phase (6,700-6,500 BP), named the settlement Boian A by the archaeologist Eugen Comşa (302). The palaeofaunistic remains, not very numerous (260 determined), come from a closed complex (303). One *Sus* species specimen dating to the Middle/Late Neolithic - Eneolithic (AA068) was analyzed in this study.

Verga 3 & Verga 5 (Neto-via Verga), Italy
Contact: Domenico Lo Vetro / Lucia Sarti

Verga 3 and Verga 5 are part of the Neto-Via Verga area within the greater Spazzavento–Neto–Via Leopardi Settlement Unit in the Sesto Fiorentino area of Italy (260). This is a multiphase Neolithic and Copper Age settlement, and is part of a broad settlement unit formed by excavation areas: Neto-via Verga, Neto di Bolasse and Spazzavento. The unit, positioned in the west of Sesto Fiorentino territory, is located on a sort of morphological top formed by depositional accumulations of two alluvial fans. The stratigraphic sequence as a whole covers a wide chronological range which indicates that the area was occupied from the beginning of the 5th millennium until the Roman period. In particular, Via Verga is a multistratified spread in 5 excavation areas: Roman, Bronze Age, Bell Beaker, Copper Age, Neolithic phases are separated by alluvial layers.

The samples are related to area 3 and Area 5 which are referred to Neolithic phase (orizzonte 7) at the end of 5th millennium AD (304); (262); (305). 3 Neolithic wild boar samples (AA638, AA639, AA640) could not be used in the analysis because the haplogroup tests failed.

Via Neruda, Italy
Contact: Domenico Lo Vetro, Lucia Sarti
The archaeological site of Via Neruda is located in Sesto Fiorentino, Italy. The site dates to the Multiphase Copper and Bronze Age (306). The site, positioned in the East of Sesto Fiorentino territory, is located in the upper part of the alluvial plain close to the foothill side of Monte Morello. Two different layers (layers F and G) testify dwelling activities within an abandoned palaeoriver bed occurred during Early Bronze Age. The samples are related to layers F and G (262). The 6 wild boar samples (AA623, AA624, AA625, AA626, AA627, AA628) could not be used in the analysis because the haplogroup tests failed.

Vinča Belo Brdo, Serbia
Contact: Vesna Dimitrijević
Vinča – Belo Brdo (44º 77' N, 20º 62' E) is situated on the right bank of the Danube, 14 km southeast of Belgrade, Serbia. It is the eponymous site for the Late Neolithic/Eneolithic Vinča culture group. Excavations started in 1908, continued sporadically until 1936, followed up in 1978–84, resumed in 1998 (307), and are currently ongoing. Sequence of cultural levels, more than 9 m deep, is mostly built up of Vinča culture deposits. At the lowermost level, a Middle Neolithic Starčevo collective burial is found, while at the top of the sequence, there are Late Eneolithic and Bronze Age deposits, as well as the late medieval necropolis. Vinča Belo Brdo is a *tell*-type site, embracing multiple horizons of large settlements composed of rectangular wattle and daub houses. Material culture is best known for elaborated pottery and anthropomorphic figurines. In the sample of animal remains from the upper levels of the Vinča culture sequence (excavation campaigns 1998-2003), pig is, after cattle, the second best represented domestic species, while wild pig is also frequent among hunted animals (308). Forty pig specimens (AA156, AA157, AA158, AA159, AA160, AA168, AA169, AA170, AA171, AA172, AA173, AA174, AA175, AA176, AA177, AA178, AA179, AA420, AA421, AA422, AA423, AA424, AA425, AA426, AA427, AA428, AA429, AA430, AA431, BLT013, BLT014, BLT021, LG058, LG180, LG181, LG183, LG184, LG669, LG679, LG681) were analyzed.

Vităneşti-Măgurice, Romania
Contact: Adrian Bălăşescu
Vităneşti-Măgurice is *tell* settlement located in a swampy area on the Teleorman valley, 7 km north of Alexandria (Teleorman County). The 5.5 m thick occupational layers were attributed to the Eneolithic Gumelniţa culture (phases A1, A2 and B1, (309). The two wild boar samples (LG423, LG426) come from the Gumelniţa A2 level (ca. 6,449-6,162 cal BP) (310); (311).

Vlasac, Serbia
Contact: Vesna Dimitrijević, Dušan Borić
Vlasac (44.53368° N, 22.05032° E) is a large open-air settlement in the upper gorge of the Iron Gates region, on the right (Serbian) bank of the Danube. The site was investigated in 1970-1 by D. Srejović and Z. Letica who examined an area of 640 m^2 along the river bank below 70 m a.s.l., which was subsequently submerged beneath the reservoir created by the Iron Gates I dam (312). Further excavations were undertaken between 2006-9 by Dušan Borić, who examined a further 326 m^2 upslope of the area excavated in 1970-1 (313). The site was occupied at various times during the Mesolithic and early Neolithic, though the majority of the 14C dates fall in the later part of the time range between ~9,200 and 7,800 cal BP. Of the 5 samples (BLT024, BLT025, BLT026, BLT0247, LG263) from Vlasac included in the present study, three (BLT025, BLT026, LG263) have been AMS 14C dated to between ~9,150 and 8,700 cal BP (170), corresponding to the early part of the Late Mesolithic (80) (81).

Voldtofte, Denmark
Contact: Kim Aaris-Sørensen, Peter Rowley-Conwy
Voldtofte is a large Early Bronze Age settlement in southwestern Fyn, located approximately halfway between Assens and Harby on West Funen. It is adjacent to "Lusehøj", one of Denmark's largest Late Bronze Age burial mounds. Excavations that took place between 1976 and 1977 yielded a wealth of material. Some possible house sites were found, in addition to nearly 100 pits containing thousands of potsherds and flints. The cereal grain assemblage was dominated by cultivated barley. Out of 3,397 bone fragments collected, 1,359 could be identified to the species. 82% of the material were of cattle (*Bos taurus domesticus*), 11% pig (*Sus scrofa*), 5% sheep (*Ovis aries*), 2% horse (*Equus caballus*), 0.2% dog (*Canis familiaris*), and 0.1% red deer (*Cervus elaphus*). Immediately striking is the almost total lack of wild game as well as the two predominant groups of cattle and pig (314). One domestic specimen (AA013) was analyzed.

Wroxeter, England
Contact: Andy Hammon
Wroxeter, *Viroconium Cornoviorum*, developed out of a civil settlement adjacent to a 1st century AD legionary fortress between about 90-150 AD to become the tribal capital of the Cornovii. It grew from the 2nd century AD to be the fourth largest town in Roman Britain, with a defined area of 78 ha. Following periods of growth and relative decline in the 2nd, 3rd and 4th centuries AD, an unusual resurgence of activity took place after AD 400. The life of the later "sub-Roman" town may have continued to about AD 650; with a phase of building in timber after the mid-6th century AD. It has been conjectured that Wroxeter had become the centre of a British, or possibly Irish, leader (315); (316). Two wild boar specimens (GL528, GL804) were analyzed.

Zambujal, Portugal
Contact: Simon Davis
Zambujal is located in the Estremadura region of central Portugal. Like most Portuguese Chalcolithic sites they are fortified settlements, dating to around 4,600-3,800 BP. The occupation at Zambujal spanned the whole Chalcolithic period, and pig is the most common taxon represented at this site (317). Five Chalcolithic specimens from this site were analyzed: GL333, GL454, GL465, GL805, and GL840.

Żegotki 2, Poland
Contact: Arkadiusz Marciniak
The site located in Kujavia, within the Polish lowlands. The site was discovered in the 1990s during the rescue excavation project preceding the construction of the Yamal pipeline from Siberia to western Europe. The major discoveries at this partially excavated site comprised the TRB and Early Medieval settlements. One TRB domestic specimen (AA134) provided successful amplification of their DNA (318). Specimens AA135 and LG497, also domestic pigs, were also analyzed.

Zelgno, Poland
Contact: Arkadiusz Marciniak
This Late Lengyel site is in Chelmno Land within the Polish lowlands. It was excavated in 2000 and 2001 as a part of large rescue excavations project preceding the construction the A1 motorway. The unearthed elements of the settlement comprise 10 trapezoidal houses accompanied by numerous pit of different character, in addition to one inhumation burial (46). Two LBK domestic pig samples were analyzed (LG730, LG732).

Zürich-Mozartstrasse and Zürich-Seefeld, Switzerland
Contact: Jörg Schibler
Zürich-Mozartstrasse and Zürich-Seefeld are two lakeshore sites at the outflow of lake Zürich in Switzerland. Today both sites are situated within the city of Zürich. In both sites a stratigraphy of several settlement layers is found. All settlements are dated by dendrochronology between the beginning of the 6th millennium BP and the beginning of the 4th millennium BP. Only two settlement layers are dated to the early Bronze Age; all other layers (Seedeld: 15 layers; Mozartstrasse: 12 layers) are Neolithic. Archaeozoological results of the animal bone from all settlements are published in (319). New interpretation of the archaeological features and the dendrochronology are published by (320). According to (320), all settlement layers 4 are dated to 3,612-3,595 BC, all settlement layers 3 to 3,126-3,098 BC and all settlement layers 2 to 2,625 -2,568 BC. The bones were provided by Kantonsarchäologie Zürich for this study.

Fourteen specimens were analyzed from Zürich-Mozartstrasse: AA200, AA201, AA202, AA240, AA244, AA246, AA247, AA248, AA249, AA250, AA251, AA252, AA253, AA254; and one specimen (AA197) was analyzed from Zürich-Seefeld.

Ancient DNA

Samples spanning a wide range of time and location, of both wild and domestic individuals, were sourced for this project (Table S1). We produced data using Sanger

sequencing (MC1R and control region of mtDNA) and with NGS in various lab, including Durham (UK), Mainz (Germany), Oxford (UK) and Stockholm (Sweden

Data generation - mtDNA - Durham

(LG/AL, SF001, SF002, SF003, SF004, SF005, SF006, SF007, SF008, SF009, SF010, SF011, SF012, SF013, SF014, SF015, SF016, SF017, SF018, SF019, SF020, SF021, SF022, SF024, SF025, SF026, SF027, SF028, SI003, SI004, SI006, SI007, SI008, SI009, SI010, SI011, SI012, SI013, SI014, SI015, SI016, SI017, SI018, SI019 in Table S1)

Samples were extracted in a dedicated ancient DNA facility at Durham University using the protocol described in (161). In brief, ll equipment and work surfaces were cleaned before and after each use with a dilute solution of bleach (5-10%) followed by water and ethanol (99%). Pipettes and plastic racks were UV-irradiated in a cross-linker (254 nm wavelength) prior to and after use. Pre- and post-PCR laboratories are physically isolated and access to the pre-PCR laboratories is restricted to Ancient DNA lab users only. Ancient DNA lab users wear clean lab coats, double set of gloves (nitrile and latex) and over-shoes in order to avoid introducing contamination from post-PCR areas.

The ancient pig remains were prepared for DNA extraction by removing an approximately two millimeter layer of the outer bone surface by abrasion using a Dremel drill with clean cut-off wheels (Dremel no 409), targeting compact cortical bone or dental dentine. The bone was then pulverized in a Micro-dismembrator (Sartorious-Stedim Biotech), followed by collection in 15ml Grainer tubes. Milling containers and grinding balls were subsequently suspended and cleaned in 1% virkon and rinsed in absolute ethanol.

Bone powder (100-400mg) was digested in 0.425 M EDTA, 0.05% SDS, 0.05 M Tris-HCI and 0.333 mg/mL proteinase K and incubated overnight (18-24 hours) on a rotator at 50°C, or until fully dissolved. The digestion buffer, excluding proteinase K, was UV-irradiated (254 nm wavelength) for an hour in a dedicated cross-linker prior to use. 2mL of extract solution was then concentrated in a Millipore Amicon Ultra-4 30 KDa MWCO (Millipore) to a final volume of 100µL. The concentrated extract was purified using silica spin-columns (QIAquick PCR Purification Kit, Qiagen) following manufacturers recommendations, except that the final elution step was performed twice to produce a final volume of 100µL. One in five or one in ten negative
extraction controls were performed alongside the ancient bone samples. All extraction blank controls were negative when PCR screened for the ANC1 fragment.

PCRs were setup in 25µL reactions using 1.25U Taq GOLD (Applied Biosystems), 1x Gold buffer (Applied Biosystems), 2.5mM MgCl2 , 0.5µg/µL BSA (Bovine Serum Albumine), 200µM of each dNTP, 0.8µM of each forward and reverse primers, and 2µL of aDNA extract. We used PCR primers ANC1 (321). One PCR negative control was included for every 5-8 aDNA template PCRs. PCR cycling conditions were 95°C for 5min, 50 cycles of 94°C for 45 sec, 54°C for 45 sec and 72°C for 45 sec, followed by 72°C for 10 min. PCR products were stored at -20°C.

An initial PCR using the ANC1 primers was performed in order to screen the extracts for preserved DNA. Successful amplifications were Sanger sequenced on the Applied Biosystems 3730 DNA Analyser at the DNA sequencing service in the School of Biological and Biomedical Sciences at Durham University. Once preserved samples were identified we used 5bp 5'-tagged PCR primers (322) to re-amplify the ANC1 fragment. In both instances PCR products were visualized on agarose gel and stained with GelRed (Biotium), and then pooled by eye into approximately equimolar concentrations using a reference series of PCR products previously quantified on the Qubit fluorometer; approximately 12μg/μL of each PCR product was used for the final pool. The pooled 5' tagged PCR products were then concentrated using an Amicon Ultra-4 30KDa MWCO filter column to a final volume of 100μL. The concentrated amplicon pool was subsequently purified using the QIAquick PCR Purification Kit following manufacturers recommendations, except that the final elute volume was 80μL.

Next generation sequencing of LG samples

The concentrated PCR amplicon pool was then built into a paired-end library (Paired-End DNA Sample Prep Kit, Illumina) following manufacturers guidelines and subsequently sequenced on the Illumina GAII platform at the Department of Biology at Copenhagen University. Illumina's Genome Analyzer Sequencing Control Software (SCS) v2.4 was used for base calling. A custom written PERL script (Rasmussen, M., University of Copenhagen) was used to filter out sequences containing the 5' tag label and to mate paired-end reads into single lines containing both
forward and reverse 5' tag label information. A second custom written PERL script was used to write a single fasta file for each tag label/amplicon. The resulting fasta files were assembled into contigs against a reference sequence (EU333163) in Geneious Pro 5.4.3. Assembly was performed using total quality score to call the best base (any base with a quality <20, equivalent to PHRED scores, was called as N and subsequently excluded from further analysis). At least one hundred sub-clones per sample were obtained for each re-sequenced PCR product, although we reached an average of several thousand copies per PCR amplicon. Nucleotide positions that could not be resolved despite the deep coverage were discarded from further analysis and called according the IUPAC nucleotide code.

Sanger sequencing

Sequencing for all other samples was carried out at the University of Oxford, Department of Zoology, Sequencing Facility on an ABI 3100.

Data generation - MC1R/mtDNA

Mainz (AP23, AP24, AP26, AP27, AP28, AP29, AP31, AP32, AP37, Aru24, Aru25, Aru10, Aru11, Aru12, Aru13, Aru7, Cav1, Cav2, Cav3, Cav4, Cav5, Cav6, Dra14, Dra15, Dra18, Dra20, Dra31, Dra32, Dra33, Dra38, Hag1, Hag2, Hag3, Kan10, Kan11, Kan12, Kan13, Kan14, Kan15, Kan16, Kan18, Kan8, Kan9, Kop8, Kop9, Kov21, Kov14, Kov16, Kov18, Kov19, Mal9, Mal12, Men4, Men5, Oko13, Oko15, Oko16, Oko1, Oko3, Ovc1, Pie13, Pie14, Pie15, Pie16, Pie1, Pie3, Pie4, Pie5, Sam10, Sam18, Sam5, Sam9, San7, San9, San1, San3, San8, Sof1, Sof2, Sof3, Tac14, Tac15, Tac16, Tac17, Tac10, Tac13, Tac6, Tac8, Uiv10, Uiv11, Uiv9, Ulu1, Ulu20, Ulu24, Ulu27, Ulu48, Ulu49 in Table S1)

Samples were processed in dedicated ancient DNA facilities at Johannes Gutenberg-University Mainz under strict rules for contamination prevention and using methods amplification and sequencing methods already described in (161). 510 bp of the mitochondrial HVSI were amplified using six overlapping primer pairs:
U15516_5'CTATCTTTAAAACAAAAAAACCCATAA3' and
L15620_5'TACATGCTTATATGCATGGGGACT3',
U15613_5'ARCCCTATGTACGTCGTGCATTA3' and
L15704_5'GCATGTTGACTGGARTTATTTGGAC3',
U15697_5'CATATYATTATTGATCGTACATAGCACA3' and
L15787_5'AAGAGGGATCCCTGCCAAG3',
U15775_5'AAYTACCATGCCGCGTGAAA3' and
L15864_5'GTTCTTACTTCAGGACCATCTCACC3',
U15852_5'TGGGGGTTTCTATTGATGAACTTTA3' and
L15941_5'TATGTGTGAGCATGGGCTGATTA3', and
U15932_5'CCCTTAAATAAGACATCTCGATGGA3' and
L16027_5'TTTGACTGTGTTAGGGCCTTTGA3'.

Primers were hybridised at 57°C. Codon 22 and 124 of the MC1R locus were amplified using the primer pairs MC1R_U44 5'AGGCTGCTGGCTTCCCTCAG3' and MC1R_L87 5'CCCGTCTGGTTGGTCTGGTT3' and MC1R_U234 5'TGGTGCAGCAGCTGGACAA3' and MC1R_L246 5'TGGAGCCGCAGATGAGCA3', respectively. MC1R_U234 was prolonged by an artificial tail (MC1R_U234_tail 5'AACTGACTAAACTAGGTGCCACGTCGTGAAAGTCTGACAATGGTGCAGCAGCTGGACAA3') (Binladen et al. 2007) to allow capillary sequencing of the only 11 bp long target sequence. U41_tail 5'CCACGTCGTGAAAGTCTGACAAT3' was used as a matching sequencing primer. Primers were hybridised at 57°C. Betaine solution 5M (Sigma Aldrich) was added to each PCR at a final concentration of 1M.

Data Authenticity of MC1R PCR data

Two independent DNA extractions were performed per sample. Both extractions had to yield a minimum of 1 successful PCR amplifications, with a minimum number of successful PCRs per sample of 3. A marker was only accepted as homozygote if all amplifications showed a homozygote signal. A marker was only accepted as heterozygote if the heterozygote signal was present at least once in each of the extractions.
MC1R results from 3 samples analysed for the MC1R loci were integrated to the dataset even though the limited amount of available DNA extract did not allow to fulfil the defined stringent authenticity criteria above: Only two sequences retrieved from the same extract of two pig samples (Cav5 and Cav6) are homozygote for not having the CC insertion at codon 22. One pig sample (Kan8) is homozygote in 1 amplification from each of the 2 extractions for having the CC insertion at codon 22. We integrated those sample to our dataset since i) extraction and PCR blank control show no evidence for contamination in any of our experiments, and ii) the data comes

from samples that yielded well-preserved and fully replicated mitochondrial DNA sequences without any evidence for in-sample or cross-contamination.

Data generation - Nuclear and mtDNA capture - Oxford

(all AL and AA samples in Table S1)

DNA extraction

DNA extractions were performed in dedicated ancient DNA (aDNA) facilities at the PalaeoBARN (University of Oxford). DNA was extracted from pig teeth or bone samples (Table S1) in a dedicated ancient DNA laboratory using the appropriate sterile techniques and equipment. Extraction was carried out using the Dabney extraction protocol (323) but with a 30 minutes pre-digest stage (324).

Library Preparation & Sequencing

Illumina libraries were built following (325), but with the addition of a six base-pair barcode added to the IS1_adapter.P5 and IS3_adapter.P5+P7 adapter pair. The libraries were then amplified on an Applied Biosystems StepOnePlus Real-Time PCR system to check that library building was successful, and to determine the minimum number of cycles to use during the indexing amplification PCR reaction. A six base-pair barcode was used during the indexing amplification reaction resulting in each library being double-barcoded with an “internal adapter” directing adjacent to the ancient DNA strand and which would be the first bases sequenced, and a traditional external barcode that would be sequenced during Illumina barcode sequencing.

Six libraries (KD033 KD037, KD025, VEM185, AA119, AA133) were additionally partially USER treated for three hours (326) before whole genome sequencing (see Table S1)

Up to 200 libraries with unique barcode combinations were pooled at equimolar levels (as determined by an Agilent Technologies 2200 TapeStation) and an 80bp run was carried out on an Illumina 2500 sequencer. Selected sample extracts were then built into libraries with a single barcode, and then sequenced individually at greater depth.We selected 12 high quality libraries for deep sequencing (Table S1). Each library was sequenced separately on multiple Hi-Seq 2500/4000 lanes (Table S1). The resulting reads were aligned to the reference genome as detailed above.

mtDNA Capture

Library pools containing up to 200 libraries were captured using MYcroarray mitochondrial MYbaits with 24 hours hybridisation at 56C, and following the MYbaits manual V3.

Data generation - Nuclear - Stockholm

(all BLT samples in Table S1)

DNA extraction

DNA extractions were performed in dedicated ancient DNA (aDNA) facilities at the Archaeological Research Laboratory, University of Stockholm, Sweden. Approximately 100-200 mg bone powder was excised per sample using a dremel drill at the lowest possible rpm. The work surface (a dead-air, UV-enabled cabinet) was thoroughly cleaned after sampling each specimen. DNA extractions were carried out

in batches of eight including one extraction blank. The bone powder was first incubated (washed) three times for 15 min under constant rotation in 500 µL 1M sodium phosphate buffer, pH 6, in order to minimise the amount of microbial contaminant DNA and to enrich for endogenous DNA(327). Buffers were replaced after each wash step and pellets were washed with 1 mL water after the final wash step. DNA was extracted following protocols published previously(328). In summary, bone powder was mixed with lysis buffer containing 0.45M EDTA (pH 8), 1M Urea, and 100 µg proteinase K, and incubated with constant agitation for at least 18 h at 37 °C. DNA was concentrated on Amicon Ultra-4 columns (Merck Millipore) and purified on MinElute silica columns (Qiagen) following manufacturer recommendations but with the addition of a second wash step. Purified DNA was eluted in 65 µL EB buffer (Qiagen).

Library Preparation & Sequencing

Double-stranded sequencing libraries were prepared from 20 µL extracted DNA following (325) but with the modification that we added 3U USER enzyme (NEB) and increased the incubation time to 3hrs at 37°C for the blunt-end repair step. The reason for doing so was to remove deaminated cytosine bases (329). The libraries were subsequently quantified using qPCR, PCR amplified, pooled at equimolar concentrations, and purified as described previously in (104). Library pools were shotgun-sequenced on the Illumina HiSeq-2500 platform at the SciLife DNA sequencing facility, Stockholm.

Data Processing

Sequence reads were aligned using Burrows-Wheeler Algorithm (BWA) version 0.7.5a-r405 (330) to Ssc.10.2, with default parameters apart from disabling the seed option ("-l 1024") (331). Samtools version 0.1.19-96b5f2294a (332) was then utilised to remove duplicates. BAM files from different sequencing lanes were merged using the MergeSamFiles tool from Picard v1.129 (http://broadinstitute.github.io/picard/), FilterUniqueSAMCons (333) was then used to remove duplicates. Depth of coverage was computed using Bedtools (334). We report alignment stats of ancient samples (including those that were only screened) in Table S1.

Sexing

We used a read depth based method based on (335), comparing alignment of sequencing reads post filtering for a mapping quality q30 to the X chromosome and chromosome 1.

MapDamage

Molecular damage was assessed using MapDamage2.0 using default parameters (336) (Figure S1). Most samples display clear signs of deamination (Figure S1), except for those that were USER treated (all BLT samples, KD033 KD037, KD025, VEM185, AA119, AA133).

Publicly available sequences

We obtained genome-wide genotype information for 149 high quality modern genomes from (337, 338) Table S2). These include 23 wild boars from Europe and the Near East: France (1), Netherlands (12), Switzerland (1), Italy (5) and Greece (4) as well as 3 from the Near East: Armenia (1) and the island of Samos (2; Near Eastern wild boars (339)), 10 wild boars from East Asia, as well as as 85 European domestic pigs that represent a broad sampling of non-commercial pigs, and commercial pigs and 24 East Asian domestic breeds. We used the genome of a *Sus verrucosus* as an outgroup (340).

High coverage ancient genomes

We used samtools 'mpileup' (0.1.19; (332) to call SNPs with default settings. Pileup files were further filtered, for each sample, using the following criteria:

- Strict minimum DoC >= 3
- Excluded all sites in region of high DoC (top 5%; taking into account sex for X chromosome genotyping)
- Excluded all sites within 3bp of an indel
- Minimum Phred >= 20
- Minimum fraction of reads supporting heterozygous >= 0.3 (value set to 0 if male for chromosome X genotyping).
- We also discarded the first and last 8bp of each read to avoid incorporating errors from deaminated sites

Ascertainment and ancient genome quality

We used only high quality ancient genome: AA288, AA363, AA133, AL718, KD025, AA119, KD033, AA451, AA349, VEM185, KD037 together with the modern genomes for SNPs ascertainment (to be called in low coverage ancient data for PCA projection, see below). In order to assess the quality of our genotyping and decide which ancient genome to include in the ascertainment we computed transversion/transition ratio at homozygous and heterozygous non reference SNPs (Figure S2). We expect this ratio to approach 0.5 for both heterozygous and homozygous. Lower genome coverage, however, clearly showed a much lower ratio for heterozygous sites suggestive that more damage were incorporated in their genotype (damage most likely incorporated as heterozygous calls). In order to limit the incorporation of deamination in our ascertainment we only used the genome of: AA119, AA133, AA349, KD025, KD033, KD037, and VEM185 (Table S1; Figure S2).

Genotypes called from these genomes were merged together with those of modern genomes using BedTools (334). Ascertainment was done without the outgroup, all non bi-allelic markers were also removed as well as markers with more than 20% missing data. This resulted in 12,522,931 SNPs on autosomal chromosomes and 213,104 SNPs on X the chromosome.

Low coverage data

We also used 54 ancient samples (Table S1) in our PCA (see below). Genotypes in these samples were called by randomly sampling a single read of 30 base pair minimum and with a mapping quality (MAQ) and base quality (BQ) of at

least 30 at each covered position in the genome, excluding bases within 5bp of the start and end of a read.

mtDNA analyses

Filtering

For the mtDNA we generated majority consensus (using reads with BQ>=20 and MAPQ>=30) sequence for all samples that had at least 2x average coverage (111 samples; Table S1) excluding bases within 5bp of the start and end of a read.

Building a database for haplogroup assignment

Our goal here is to build a robust method to assigned haplogroup to low coverage mtDNA data (Table S1) using phylogenetics. To do so we first build a data-base of high quality modern and ancient mtDNA.

We obtained the full mitogenome of 77 pigs previously published in (337, 338) (Figure S3) and combined this data with 111 newly sequenced mitogenomes (including all pigs with coverage greater or equal than 2x; Table S1). Altogether these data set contain 188 full genomes from European and Asian wild and domestic pigs.

Phylogenetic analysis and reference sequences

We used RAxML (version 8)(341) to build a maximum likelihood tree, with 100 bootstrap replicates under a GTR-GAMMA model. The resulting tree is presented in Figure S3. In the tree we find a high support (bootstrap value >=90 in all case but root of Y1 which is >70) for the 6 major mtDNA haplgroups in *S. scrofa*, namely European (Eu), Y1, Y2, ArmT, Italian (It) and Asian (As; Larson *et al.* 2005, 2007; Ottoni *et al.* 2012) (Figure S3). We then selected two high quality genome sequences as reference for each haplogroup:

Haplogroup As - found primarily in East Asian pigs (but also in Iran; Figure 1a). We selected WB30U09 and WB29U14, two high quality sequences from Chinese wild boars (Bosse *et al.* 2014; Frantz *et al.* 2015) as reference sequences.

Haplogroup Eu (including ANC-Aside and ANC-Cside) - found primarily in European populations, this haplogroup is found in the vast majority of ancient and modern European wild boars as well as a majority of European breeds (Figure 1a; (321, 342)). We selected WB21F03 and WB25U11, two high quality sequences from Dutch and French wild boars respectively (337) as reference sequences.

Haplogroup It – found primarily in Italian and northern balkan wild boars (Figure 1a). We selected WB42M09 (wild boar from Italy) and AA457 an ancient wild boar from Croatia that was previously assigned to this haplogroup based on its control region (GL979 in (321).

Haplogroup Y1 - found primarily in Near Eastern (e.g. Turkey) wild boars and ancient European domestic pigs (321, 342). We selected WB33U05, a wild boar from the Island of Samos (337) as the region is known for its high frequency of Y1 haplogroup (339).We also selected the sequence of Turkish wild boar (AL718;

coverage >220x) as this sample was previously assigned to Y1 based on its control region (321).

Haplogroup Y2 - Found in ancient wild boars from the south of Europe (161, 321). In Figure S3 this clade (in purple) can be identified by the sequence BLT025 (control region published as LG260 in (161), from the site of Vlasaac (Serbia). We selected two high coverage reference genome for this clade, AA479 (>228x) and AA480 (>68x) two ancient Ukrainian wild boars (Figure S3).

Haplogroup ArmT (including Arm2T and Arm1T)- Found mainly in wild and domestic pigs from the Near East (e.g. Armenia, Iran, Turkey) as well as in ancient European domestics (161, 321). In Figure S3 this clade (in green) can be identified by the sequence AA364 (control region published as LG419 in (161), from the site of Kohneh Tepesi (Iran) and AA119 (control region published as GL896 in (321) from the site of Lchashen in Armenia (Figure S3) . We selected two high coverage reference genome for this clade, AA363 (same site as AA364; >280x) and AL1107 (>29x) from the site of Cadir Hoyuk in Turkey (Figure S3; Table S1).

Haplogroup NE1 - Found uniquely in modern wild boars from Iran, Iraq, Southern Levant and South Caucasus (161). This haplogroup was never found in modern or ancient domestic pigs from either Near East or Europe (161). The sole full mtDNA genome available representative of NE1 was GL943 (pink), obtained from an egyptian wild boar (Figure S3; Table S1).

A method for rapid haplogroup assignment

We used the sequences described above to rapidly assigned haplogroup to all other mtDNA sequences (partial or full). To do so, we first build a maximum likelihood phylogenetic tree (using RAxML) based on the references sequences (see above) and a query sequence (the sequence we want to assign to a haplogroup). We then compute the pairwise distance between our query and each reference haplogroup sequences in the phylogeny using Dendropy (version 4) (343). We then assign the haplogroup of the reference sequence that was the closest to our query sequence.

We tested the performance of our simple method using 182 sequences with known haplogroup (based on phylogenetic analysis; see above; NE1 was excluded given we only had one sequence). We recovered the right haplogroup for every sequences, demonstrating that our method provide a robust assignment for high quality data. We then tested how missing data affected our success rate. To do so, we randomly selected 250, 400, 800, 1600 and 3200bp (not necessarily consecutive) from each 182 sequences with known haplogroup. We repeated this 100 times per length / genome combination and assigned a haplogroup to these sequences with various degree of missing data. Our method recovered the right at rate of 83.8%, 92.7%, 98.2%, 99.7%, and 99.8% for sequences of 250, 400, 800, 1600 and 3200bp respectively. Our recovery rate per clade was also high (>90% for each clade based on 800bp; Figure S4). Based on this analysis we decided to use 800bp as a minimum length for haplogroup assignment for our low coverage data.

We further tested this method on empirical data. To do so, we compared mtDNA haplogroup for 116 samples as inferred using PCR (Table S1) to our NGS based

assignment. Our method retrieved the right haplogroup for 107 out of 116 samples for which we had both PCR and NGS based haplogroup assignment (>92% success rate; Table S1). Lastly, we checked for mismatch between PCR and NGS sequence data in sample with >1x coverage genome wide (29 samples in total). Although one sample low coverage sample (~1%) had up to 4% mismatch between NGS and PCR these were mostly transition and transversion rate of mismatch were below 1% in all samples (Fig. S5).

Low coverage data

Many sequences did not pass the stringent criteria of 10x depth of coverage (Table S1). Nevertheless, as we shown above, only few reads should provide enough resolution for a robust haplogroup assignment. We thus, lowered our requirement from 10 to 1 reads with MAQP>=30 and BQ>=30 and created a sequence consensus for all samples that did not pass our filtering criteria detailed above. All samples that yielded at least 800bp of coverage, based on this filtering, were assigned a haplogroup using the approach described above.

mtDNA lineage geographic distribution (Figure 1)

We grouped wild boars haplogroups, from our study (Table S1) and from publically available data (Table S1) into four meta groups, including Europe (mt-Eu, and mt-Italy), Y2, Near East (mt-ArmT and mt-Y1) and Asian (As) as previous studies demonstrated that Eu and Italian haplogroups were mostly restricted to European wild boars while mt-ArmT and mt-Y1 were restricted to wild boars found in the Near East (161, 344) (also see Figure S6-7). Aside from a few examples of wild boars carrying mt-Y2 (6/99) haplogroup in the Near East we found that most Near Eastern wild boar carried either mt-Y1 (54/99) or mt-ArmT (39/99) (Figure S6-7).

To further test this phylogeographic signal, haplogroup membership was then transposed to create an ancestry matrix as in (345). This matrix was then plotted onto a map with a tessellated projection, using the R package "tess3r" (346–348). This projection demonstrate a highly pronounced population structuring between Near East and European wild boars (Figure 1a). Following haplogroup assignment of both low and high coverage data, as well as from PCR results we plotted meta group assignment of domestic pigs on the wild boar projection (Figure 1b,c,d).

Samples excluded from Figure 1

EU haplotype in wild boar found in the Near East

ARD3 and ARD4 (349) are modern wild boar with mt-Eu haplotype (ANC-Aside) found in Iran. GL141 is from Armenia; GL753 is from Iran; GL766 and GL779 are from Iraq yet possesses a EU haplotype. This sample was excluded from analysis previously (321) (although GL141 was reported as haplotype A) and was justified as: "*the turnover of domestic pigs in Armenia from domestic pigs of Near Eastern origin to those of European origin before the end of the Iron Age in Romania suggests that pigs domesticated in Europe were transported east into the Near East before or during the Iron Age. This eastward movement of European domestic pigs increases the likelihood of discovering European signatures in samples that superficially appear to be modern wild boar from the Near East, but are in fact feral descendants of domesticates introduced from Europe during or after the Iron Age.*"

GL935 is a wild boar that was found in the north slope of the West Caucasus, and GL944 is a wild boar from Georgia. Because both samples have mt-Eu haplotypes and are from the Near East, we have excluded them from this analysis for the same reason as explained previously (321). AA315 and AA316 are wild boars from Gobekli Tepe, Turkey. The PCR worked failed and there was low NGS coverage (0.98x). Our NGS analysis showed it as mt-Eu, but phylogenetic reconstruction showed that the haplotype is even more basal than mt-Eu, suggesting a potential new haplogroup. We were unable to definitively classify the samples. Also the age of the sample is unknown insecure/direct dating is necessary.

AA308 is a wild boar from Bademagaci, Turkey, dating to 7,000-6,000 BC. The PCR work failed and there was low NGS coverage (0.48x). The NGS analysis showed it as EU, but phylogenetic reconstruction showed that the haplotype call is even more basal than EU, suggesting a potential new haplogroup. We were unable to definitively classify the sample. AA399 is a wild boar from Suberde Hoyuk, Turkey, dating to 7,500-7,000 BC. The PCR work failed and there was low NGS coverage (0.24x). The NGS work showed it as mt-Eu, but phylogenetic reconstruction showed that the haplotype call is even more basal than EU, suggesting a potential new haplogroup. We were unable to definitively classify the sample. Tp1 and WBTR_514, two wild boar samples from Turkey, also have mt-Eu (specifically ANC-Aside) haplotype (161).

Y1 haplotype in wild boar found in Europe

AA41 was found in Siniarzewo 1 site, Poland, dating from 4,100-4,400 BC. AA379 was found in Buran-Kaya 4 site in Ukraine, dating from 7613-7484 cal. BP (OxA-24692). LG610 was found in Bruschal, Germany, dating from 4,200-3,400 BC. . LG829 is also a wild boar found at the same site, except the sample is older, dating to 5,200-4,500 BC. Oko16 was found in Okoliste, Bosnia-Herzegovina, dating to 5,100-5,000 BC.

AA466 has an unclear status. It is assumed to be from a Mesolithic context, although some Neolithic context and domestic pigs were also found at the same site, Bern-Birsmatte, Switzerland. LG595 was found in Klingenberg, Germany, dating to Late Neolithic contexts, 4,000-3,650 BC. AA159 was found in Vinča Belo Brdo, Serbia, dating to Neolithic contexts, Mid 5th Millennium BC Uiv11 was found in Uivar, Romania, dating to the Late-early Neolithic, 5520 ± 26 calBC (KIA-42156: 6560 ± 35 BP). AA497 was found in France, with French middle Neolithic (Northern Chasseen culture) contexts, dating to the early 4th millennium BC. All of these wild boars are found at sites on which domestic pigs were also found - as previous studies suggested, this is likely the result of domestic to wild admixture (350, 351).

Asian haplotype found in wild boar in Europe

DQ379240 (352) was excluded as it is an Asian haplotype in Belgian wild boar. Kbl020 (accession JF774408; (339)), a Greek wild boar sample was excluded as although the paper gave the sample an Asiatic haplotype call, 2 unique mutations suggests that it is an unknown haplogroup.

Nuclear DNA analyses

MC1R and domestic/wild status

We tested the usability of a non-synonymous mutation (D124N that is the result of a G->A mutation at the second codon position) in the *MC1R* gene (leading to black coat colour and loss of camouflage coat colour (353)) as a diagnostic of wild/domestic status. We first computed the frequency of this mutation in a total 222 modern wild and domestic pigs, including genomes used in this study as well as MC1R sequences obtained from (353). We found that the frequency of the derived allele (A) in European domestic (EUD) was >85% (Figure S8). If we take into account breeds that are known to have been selected to have camouflage coat colour (*e.g*. mangalitsa) or breeds that have other derived coat colour phenotypes (*e.g.* red coat colour such as Duroc) (353), the frequency increased to >95%. In contrast, the frequency of the ancestral allele (G) in European wild boar (EUW) was >98% (Figure S8). In fact out of 29 wild boars, we found only one sample (Dutch wild boar) with an heterozygous genotype. We also note that none of the three boars from the Near East and four wild boars from the Balkans possessed the derived allele (Figure S8).

We enriched this data with information from NGS and PCR based assay of ancient samples (see Table S1). For the NGS we counted the number of high quality read (MAPQ>= 30 and BQ>=30) for each allele to obtain a consensus (see Table S1).

This allowed us to 1) assess the robustness of this diagnostic mutation to discriminate between wild and domestic 2) determine the status of samples that could not be classified as wild or domestic based on morphology/context alone. Similarly to the modern data, we found that all ancient wild boars (17) that had been sampled in this study possessed the ancestral allele (G). This included wild boars from Mesolithic Germany, Neolithic/Chalcolithic Ukraine, Romania, Bulgaria, and Turkey, as well as Bronze age Turkey.

Of the 76 domestic samples that we typed for this allele, 57 were homozygous for the derived allele (A) and 7 heterozygous while 12 were homozygous for the ancestral allele (G). Homozygous derived and heterozygous (which express dominant black phenotype) were from a variety of sites, including 43 from Norse period sites in Iceland and Faroe, 13 from a Neolithic/Chalcolithic context in Turkey, England, Romania, Bulgaria, Germany, and Georgia, 6 from a Bronze age context in Turkey, England, Armenia, and Georgia, and from later period (Medieval and Hellenistic) from Turkey and the Netherlands. Of the 12 samples that were homozygous ancestral, 6 were from Neolithic/Chalcolithic context including sites in Georgia, Romania and Turkey and 6 were from Bronze Age sites in Turkey and Romania.

Altogether, these results demonstrate a clear division between wild and domestic samples based on this diagnostic mutation. The dominant black phenotype seem variable in domestic pigs from the Neolithic/Chalcolithic (~68% in Europe and Near East) and in the Bronze Age (~50% in Europe and Near East), but becomes fixed in later period. In addition, we found only one heterozygote wild boar out of 29 modern and 17 ancient samples. This fits very well with previous studies that found the dominant black allele in wild boars at a frequency of 5% in Greece (354) and less than 1% in Luxembourg (355). Altogether this suggests that while the ancestral allele

cannot distinguish between wild and domestic pigs, at least in the ancient data, the derived allele is highly diagnostic of a domestic status. Using this rationale, we infer the status of two samples, for which we had genome-wide data, AA288, a sample from Neolithic France, and AA363, a sample from Bronze Age Armenia.

Given that both European and Near East domestic pigs have the derived D124N allele we investigated its origin. To do so we built an ultrametric tree (using phangorn (356)) of haplotypes from a 50kb region around the *MC1R* gene using the phase of all our high ancient coverage genome data and modern data (see methods section *Chromopainter and GLOBETROTTER* for details about the phasing). Interestingly besides a few examples, most haplotypes (149/153) that had the derived allele formed a monophyletic group (Figure S9). This group included all ancient European and Near East domestic pigs as well as the majority of modern domestic pigs. This suggests that the allele shared by the majority of these pigs was the result of a single mutation. Interestingly, the wild boar haplotypes that were the closest to this group (besides the haplotype from the Dutch wild boar that possess the derived allele and which is most likely the result of recent introgression from domestic into wild (357)) were found in samples from both Near East (WB33U04) and Europe (WB22F02, WB22F03).

Thus, while we cannot definitely ascertain that this haplotype originated in the Near East, it seems plausible, that this haplotype, on which the derived allele first appeared, originated in the Near East. Especially given that an early Bronze age Iranian (AA363, 4468-4279 BP) and a Bronze Age Armenian sample (AA119) share the exact same haplotype as many ancient and modern domestic pigs from Europe. In addition, we also found multiple domestic pigs from Neolithic Anatolia that carry the derived allele (see above; Table S1). Alternatively, it is possible that the mutation was brought into the Near East from Europe, this, however, seems unlikely as European were only brought into the Near East later during the Iron Age (161).

Ancestry analysis

Neighbour Joining tree

For this analysis, we used all ancient and modern high and medium coverage data including the ancient genomes: AA288, AA363, AA133, AL718, KD025, AA119, KD033, AA451, AA349, VEM185 (Table S1). We used plink (358) to compute an Identity By State (IBS) matrix based on our high genome coverage (Table S1) and modern genomes (Table S2). This matrix was used to build a neighbour joining tree (NJ) using the R package “ape” (359). To compute support for each node we bootstrapped our ped file 100 times and re-computed a NJ tree each time (Figure S10). For this analysis we used only transversion removed linked SNPs by enforcing a distance of 1,000 bp between SNPs using plink. This resulted in a set of 1,055,138 SNPs.

Modelling population history of wild boars

For this analysis, we used all wild boar ancient/modern, high/medium coverage data including the ancient genome: AA133, AL718, AA349 (Table S1) and the same 1,055,138 SNPs as for the NJ analysis. In order to understand the ancestry of

domestic pigs, we first established the relationship between wild boar populations in Western Eurasia. Based on our NJ tree above and mtDNA analyses (Figure 1a; Figure S7&9), we define three populations, Near Eastern (NEW), European (EUW; including wild boars from France and the Netherlands; Table S2), Balkan (BLW) and Italy (ITW; including Swiss wild boars) and Asian (ASW) as suggested by our mtDNA analysis. We then ran ADMIXTURE with K=1 to K=10. We used a 10 fold cross validation approach to assess the best K value (Figure S11a). Our cross validation suggested that the best K values were 2 and 3. At K=2 ADMIXTURE splits Western populations (EUW, NEW, BLW, ITW) with Eastern populations (ASW; Figure S11b). Interestingly, this analysis suggests that NEW share some ancestry with ASW, which is also suggested by the fact that Iranian populations of wild boars share the same mtDNA haplogroup as ASW (Figure 1a). At K=3, ADMIXTURE splits NEW and EUW population (red and yellow ancestry in Figure S11c). This analysis indicates that BLW and ITW populations are a mixture of NEW and EUW ancestry, with the former being ~30% NEW and the latter ~10% NEW. Ths analysis also demonstrates that NEW ancestry has been stable for at least ~10000 years, as demonstrated by the result of AL718, a wild boar from Aşıklı Höyük (Figure S11c).

To better understand patterns of gene-flow between NEW, BLW and ITW, we used *qpGraph* to model relationship between Wild boar populations (360). We divided our wild boars into 5 populations based on Figure S10, NE2 (Iran and Armenia), NE1 (Turkey and Samos), BLW (Balkan), ITW (Italy and Switzerland), EUW (rest of Europe). We used *S. verrucosus* as an outgroup. We first fitted a simple model with no admixture (Figure S12a). This model left 11 outliers (Figure S12a). We added admixture from NE1 into BLW (as suggested in Figure ADMXITUREa). This model (Figure S12b) left a few outliers involving the Italian wild boars and Near Eastern populations. This analysis support the above results that the wild boars from the Balkans (BLW) are a mixture of NEW and EUW and further indicate that the best source for the NEW ancestry in NEW is the NE2 population (Turkey and Samos). Adding admixture from the Balkan into Italy removed these outliers (Figure S12c). This further support the result of the ADMIXTURE analysis (Figure S11).

Low-coverage data - testing PCA

Here we want to project low coverage data for which we have only few SNPs covered. To do so, we first selected all our low coverage samples that had at least 10 covered position from the ~12M autosomal SNPs we identified using high coverage samples (see *Ascertainment and ancient genome quality* section above). We then randomly selected a maximum of 100,000 SNPs per sample, that were covered by at least one read (see Table S1 - used-PCA, for a list of samples; note that the majority of samples had less than 100k SNPs covered from our 12M SNP panel). We then merged these lists of SNPs among our low coverage samples - this resulted in 1,534,393 SNPs.

We then assessed the accuracy of smartPCA (361) when projecting low coverage sample using lsqproject=YES option and these 1,534,393 SNPs. To do so, we estimated the position, on the PC space, of sub-sampled high coverage samples. We sub-sampled 10, 50, 100, 200, 500, 1000, 2000, 5000 and 10000 SNPs 1,000 times for each sample and projected these sub-sampled genome on the PCA (PC1 and PC2). For each sample we then computed the distance of the projected data and the expected

position of the full data on each PC. We then divided the difference by the total size of each PC and estimated the mean combining both PC1 and PC2. This analysis was run without the East Asian pigs.
Our results show that, using these 1.5M SNPs the PCA projections are highly accurate when a sample only 5,000 callable SNPs (Mean Error of ~1%; Figure S13). We thus chose to use all samples with at least 5,000 SNPs to be projected on the PCA (Figure S14; Table S1).

PCA

Based on the results above, we used every sample that had at least 5,000 SNPs covered (out of the 1,534,393 SNPs). This resulted in a total of 203 samples, including 54 low coverage genomes. We split the populations into ASD (Asian domestic), ASW (Asian wild), BLW (Balkan wild), BLW-A (Balkan wid ancient), EUD (European domestic), EUD-A (European domestic ancient), EUU-A (European undermined [domestic/wild] ancient), EUW (European wild), EUW-A (European wild ancient), NED-A (Near East domestic ancient), NEU-A (Near East undermined [domestic/wild] ancient), NEW (Near East wild), and NEW-A (Near East wild ancient). On Figure S14a, PC1 separates European and Asian pigs, and PC2 Near East and European pigs. On Figure S14b we find that NEW(-A), NED-A and NEU-A are separated from EUW, EUD(-A), BLW on PC2. Most EUD and EUD-A are clearly clustering with EUW rather than NEW. Some EUD-A from the Neolithic, however, (blue circles) are clearly intermediary between EUW and NEW, suggesting they share ancestry from both NEW and EUW (this includes KD033 as indicated Figure S10 and Figure S15 and Figure S16). PC1 on Figure S14b, however, most likely represent ascertainment bias in European domestic and Asian strong asian admixture (338, 362, 363)(Figure S15-5) - as it splits EUD from from the rest of the populations.

ADMIXTURE

For this analysis, we used all wild boar ancient/modern, high/medium coverage data including the ancient genome: AA288, AA363, AA133, AL718, KD025, AA119, KD033, AA451, AA349, VEM185 (Table S1) and the same 1,055,138 SNPs as for the NJ analysis to compute ancestry proportion using *ADMIXTURE* (364). We used a 10 fold cross validation approach to assess the best K value (Figure S15a). The best K value was 4. Interestingly at K=4, ADMIXTURE does not separate EUW and NEW populations (Figure S15b). Instead it separates between breeds of domestic pigs, in particular, between Large White and other breeds. This is most likely an effect of ascertainment bias given the large number of EUD in this analysis and/or potentially influenced by strong drift (due to artificial selection) in domestic population (337, 365). NEW and EUW are separated at K=6 (Figure S15c). This analysis recapitulate our finding discussed above (Figure S11). We found that the two high coverage Near Eastern domestic pigs (NED) from the Bronze Age of Armenia (AA119) and Iran (A363) possessed 100% NEW ancestry.

We found Near East ancestry in 8 modern EUD samples. These include breeds from Hungary (Mangalitsa [6.4-4.9%]), Italy (Casertana [6.2-1.7%] and Cinta Senese [~3.6%], and Calabrese [~5.2%]) and Spain (Negro Iberico [~1.9%] and Retinto [4.5%]). NEW ancestry in these populations, however, was always lower than in modern ITW (~6.5-8.5%) or than in modern BLW (30-36%).

With the exception of KD025, a sample from the Netherlands, we found NEW ancestry in all ancient domestic pigs from Europe. This includes a medieval pig from the Faroe island (AA451; ~5%), a Neolithic pig from Britain (VEM185; ~10%), and two Neolithic pigs from Germany (KD037: ~9% and KD033: ~54%). Interesting, KD033 also had a Near Eastern haplogroup (Y1; Table S1).

We also ran *ADMIXTURE* on a subset of our data - in particular we pruned out European domestic pigs (EUD) as they make up most of the data set keeping at most 2 genome per breed (Figure S16). We used a 10 fold cross validation approach to assess the best K value (Figure S16a). Our cross validation suggested that the best K value is 3. At K=3 ADMIXTURE identify three populations roughly corresponding to 1) Asian pigs (ASW/ASD; blue in Figure S16a), European and Near East wild boars (NEW/EUW; red in Figure S16a) and European domestic pigs (EUD; pink in Figure S16a). This shows that the result presented in Figure S15 (splitting EUD populations before NEW/EUW) analysis above (with full data set) was indeed affected by an over representation of EUD in the data.

F3 & D-statistics

For this analysis, we used all wild boar ancient/modern, high/medium coverage data including the ancient genome: AA288, AA363, AA133, AL718, KD025, AA119, KD033, AA451, AA349, VEM185 (Table S1) and the same 1,055,138 SNPs as for the NJ analysis. Figure S10 suggests that most EUD are equally related to both NEW and EUW (EUD is outgroup to EUW and NEW). In addition, Figure S15b suggests that EUW is more closely related to NEW than EUD. Here we used outgroup-f3 to further assess whether this is the result of the know admixture from ASD (here we use ASW as proxy for ASD to avoid issues with gene-flow EUD->ASD) into EUD during the 19th century (338, 362, 363). Outgroup-f3 were computed as (*S. verrucosus* [outgroup species]; [ASW/EUW/NEW], EUD). This statistics measure shared drift between two populations (here ASW/EUW/NEW and EUD). The higher the value the more closely related the two populations tested. We plotted every possible combination of this test for each EUD genome (Figure S17). Our results for show that every EUD genome (except KD033, a Neolithic domestic pig from Germany) is more closely related to EUW than to NEW and ASW. However, we can see that many of the modern EUD have higher f3 value with ASW than ancient domestics (EUD-A) or than breeds such as Mangalitsa (MA01F20 and MA01F18 in Figure S17) a traditional breed from Hungary that is known to be non admixed with Asian pigs but also seem to possess NEW ancestry (Figure S15). Altogether this indicates that the placement of EUD in Figure S10 is most likely an artefact of strong admixture between ASD and EUD.

We further used D-statistics to assess whether admixture from ASD would affect our power to detect any remaining NEW ancestry in the genome of modern domestic pigs. To do so, we computed every combination of D(S.verrucosus, [NEW/ITW/BLW/ASW]; EUD, EUW) for each EUD genome. Positive values means admixture from NEW/ITW/BLW/ASW into EUW and negative values indicate admixture into EUD. In Figure S18 we plotted the distribution of Z values of this statistic (plotting all possible combinations). We found that, most EUD genome have high negative Z values (Z<<-4), when using ASW, indicative of gene-flow from Asian pigs (Figure S18). These values were mirrored by high positive Z when using NEW/ITW/BLW (Z>>4). This, however, is not the result of admixture from NEW

into EUW since the MRCA of EUW/EUD but instead most likely the result of the admixture from Asian pigs into EUD, which makes EUW and NEW artificially more related relative to NEW/EUD. This indicates that a D-statistic test is unlikely to be able to assess whether most EUD have any remaining NEW ancestry or if the NEW ancestry can be distinguished from admixture with ITW/BLW.

In some cases, however, traditional breeds such as Mangalitsa (MA in Figure S18) show non-significant values (3<|Z|<3) for admixture from both ASW and NEW. In fact, the only genome that we find with significant evidence for admixture from NEW was KD033, a Neolithic domestic pig from Germany (Figure S18), which has according to our ADMIXTURE analysis possess ~54% Near Eastern ancestry (Figure S15b). All other ancient genomes seem to possess admixture from ITW rather than BLW or NEW. Altogether this suggest that the NEW ancestry in modern EUD found in our ADMIXTURE analysis is likely due to admixture from European wild boars that possess some degree of NEW such as in Italy or in the Balkan (Figure S11c).

Phasing

We used the approach implemented in *GLOBETROTTER (366)* to model the ancestry of EUD as a function of EUW, NEW and ASW. To do so, we first phased all using *SHAPEIT2* (367) with default parameters and using the recombination map from (368). We used all the wild boar ancient/modern, high/medium coverage data including the ancient genome: AA288, AA363, AA133, AL718, KD025, AA119, KD033, AA451, AA349, VEM185 (Table S1). We restricted this analysis to transversion (~4M SNPs).

Chromopainter and GLOBETROTTER

We then used *ChromoPainterV2 (369)* to paint the genome of all EUD (recipients) using NEW, ASW and EUW as donors. To do so, we first estimated switch parameter over global mutation rates by taking weighted average values (weighted over number of SNPs per chromosome) of the final value given by running *ChromoPainterV2* using 10 E-M iterations (-i 10 -in -iM) using all EUD samples on chromosome 1 and 2. We used these values (-n 7249.57 -M 0.00784444) to paint the chromosome of EUD ("target") using NEW, ASW and EUW as donors. We also painted every NEW, ASW and EUW genome using the same set of donors and combined the resulting files for *GLOBETROTTER*. We used these painting profile to compute to admixture proportion of all EUD genomes (without inferring admixture date: num.mixing.iterations=0) as in (370). *GLOBETROTTER* found that the EUD ancestry can be modelled as ~4% NEW, ~26% ASW and, ~60% EUD. The ASD proportion is very close to previous estimates (362, 371, 372).

ADMIXTURE simulations

Here we want to estimate the minimum amount of NEW that we could detect using *ADMIXTURE* in modern European domestic. To do so, we simulated admixed genomes. To simulate an admixed genome, we mixed a random NEW and EUW genome as described in (373). Starting at the beginning of a chromosome we sampled a genotype from a random NEW genome with a probability of α or from EUW with a probability of 1-α. We note that α is the NEW ancestry in the simulated genome. Along the chromosome, at each SNP we re-sampled a new ancestry (either NEW or EUW) with a probability of $1-e^{-\lambda g}$ where λ is the number of generation since

admixture and g the distance in Morgan of the whole block. We used the recombination map described in (368). We simulated 1000 genome for each possible combination of values, with λ: 100, 500, 1000, 2000, 4000 and with α: 0.01-0.1, 0.15.

For each simulated genome, we then estimated NEW ancestry using all NEW and EUW samples with ADMIXTURE (K=2). We then estimated, for each combination of λ and α, the rate at which ADMIXTURE detected NEW ancestry (with ancestry > 0.00010). We then estimated the probability of observing 8 out of 85 genomes (modern data estimate) with NEW ancestry given the rate for each combination of λ and α using a binomial distribution. Our results indicate that the number of EUD with NEW ancestry is compatible with 4-5% NEW ancestry in modern European pigs (Figure S19a).

GLOBETROTTER simulations

As for *ADMIXTURE*, we estimated the minimum amount of NEW ancestry that our *GLOBETROTTER* analysis could detect in EUD. To do so, we simulated admixed haplotypes as described above (170 for 85 EUD) with the same range of λ and α as above. We joined two haplotypes (sampled from the same pair of NEW and EUW) to create a diploid individual and painted each diploid individual using NEW and EUW as donors, excluding the sample that were used for the simulation. We then painted every NEW and EUW genome using the leave one out approach described in (366). Briefly, to ensure that we painted the simulated genome and surrogate genome with the same number of donors, we painted each NEW/EUW with all but 2 randomly selected sample (NEW or EUW). Each painting profile were then used to estimate NEW and EUW ancestry proportion using *GLOBETROTTER* (without LD curve fitting; num.mixing.iterations=0). We repeated this 10 times for each λ and α combination and estimated the proportion of runs for which *GLOBETROTTER* detected NEW ancestry. Our results indicate that *GLOBETROTTER* cannot detect NEW ancestry below 4% and can detect NEW 80% of the time when above 6% (Figure S19b). This suggests that our result from *GLOBETROTTER* (~4% NEW ancestry in EUD overall) is accurate.

Sweep ancestry

Here we want to assess whether selection, during the evolutionary history has acted on haplotypes that were of Near Eastern origin. To do so, we used previously detected selective sweeps regions that had a *p-value* < 0.01 (299 sweep regions in total (337)). For each region, we extracted 25Kb around the peak (as in (337); 50kb in total) and computed Kimura-2-parameter model corrected nucleotide distances between every pairs of haplotypes using the R package ape (359) (see Figure S21 for examples of UPGMA trees computed using this distance). Foreach EUD haplotype we then computed *D*, the normalised difference between the nucleotide distance of this haplotype to the closest EUW haplotype and to the closest NEW as:

$$D = (d_{NEW} - d_{EUW}) \,/\, (d_{EUW} + d_{NEW})$$

Where d_{EUW} is the distance to the closest EUW haplotype and d_{NEW} is the distance to the closest NEW haplotype. *D* value range between -1 and 1 with positive value

meaning that the haplotype is closer to EUW and negative value meaning it is closer to NEW. To plot these results we computed mean and standard deviation of *D* over all EUD. The results are presented in Figure S20. This figures shows that none of the sweeps found in (337) affected haplotypes that are of clear NEW ancestry. In fact we found only 9/299 regions in which D was on average lower than 0, with no region with both mean-standard deviation were lower than 0. On the other hand we found 270 regions with mean greater than 0 and 158 regions in which both mean and mean-standard deviation were greater than 0.

X chromosome

A recent study identified a region on the X chromosome with extremely low recombination rate (374). This large ~50Mb region displayed a very peculiar pattern of divergence where *S. scrofa* from South China clustered together with other *Sus* species from ISEA. This pattern was suggested to be the result of either ancient admixture and/or retaining ancestral state due to low recombination rate. Here we assessed whether this low recombination rate could have allowed EUD to retained NEW ancestry. We first assessed whether this region has either low or high divergence between wild boar populations. To do so, we computed d_{xy} (375) between these three populations in 500kb bins across chromosome 1 and X (Figure S22). We found that the region, in between 50 and 100Mb on chromosome X, described by (374), indeed has a very low divergence rate, between all three populations, compared to other region. As opposed to wild populations in China, however, this region does not seem to display relatively higher divergence rate between NEW and EUW.

We then build NJ trees using identity by state with data from i) the entire chromosome X, ii) the entire chromosome X except for the low recombining region, iii) only the low recombining region. To limit the impact of missing data we used the same set as for the *ADMIXTURE* analysis (see above). We found no difference between the trees build using data in any of these regions (Figure S23) and those built using autosomal chromosomes.

Supplementary Tables

Table S1.

Table describing data used in this paper. Extract No. / Lab code: Code used in this paper. **Previous Extraction Code:** Extract code used in previous publications. **Accession of mtDNA data:** Accession code for mtDNA data if previously published (and provided in the reference). **Source:** Author that provided the material (see site description for more information). **USER treatment:** Y=library was user treated; N=library was not used treated. **Haplogroup based on NGS data:** haplogroup call based on methodology described above. **Control haplotype and Control haplogroup**: haplotype and haplogroup based on control region. **Combined haplogroup**: consensus haplogroup if data from NGS and control region available (control region haplogroup was used if disagreement). **Clade**: Clade used in Figure 1. **Mismatch NGS / control haplogroup:** 0=both method agree; 1=methods disagree. **Capture Status**: indicate whether the library was capture for mtDNA. **SNPs Covered:** Number of autosomal SNP covered. **used-PCA:** indicates whether a sample was used in PCA. **use-other-nuc:** indicates whether a sample was used in other nuclear analyses. **Group:** meta group to which the sample belong: ASD=Asian domestic, ASW=Asian wild BLW=Balkan wild, BLW-A=Balkan wild ancient, EUD=European domestic, EUD-A= European domestic ancient, EUU-A=European unknown (either wild or domestic) ancient, EUW=European wild (including Italian), EUW-A=European wild (including Italian), NED-A= Near Eastern domestic ancient, NEU= Near Eastern unknown (either wild or domestic) ancient, NEW=Near Eastern wild, NEW-A=Near Eastern wild ancient. **Number of reads MC1R (A allele):** number of reads mapped at the D124N variant that possess the A allele. **Number of reads MC1R (G allele):** number of reads mapped at the D124N variant that possess the G allele. **Genotype MC1R:** Predicted MC1R genotype. **Status based on Zooarcheolgy:** Domestic, wild or feral status based on information from context and morphology (see site description for more information). **Status based on MC1R and Zooracheology**: consensus status based on both MC1R and zooarcheology call.

Table S2.

Summary of the modern genome used in the analysis.

Supplementary Figures

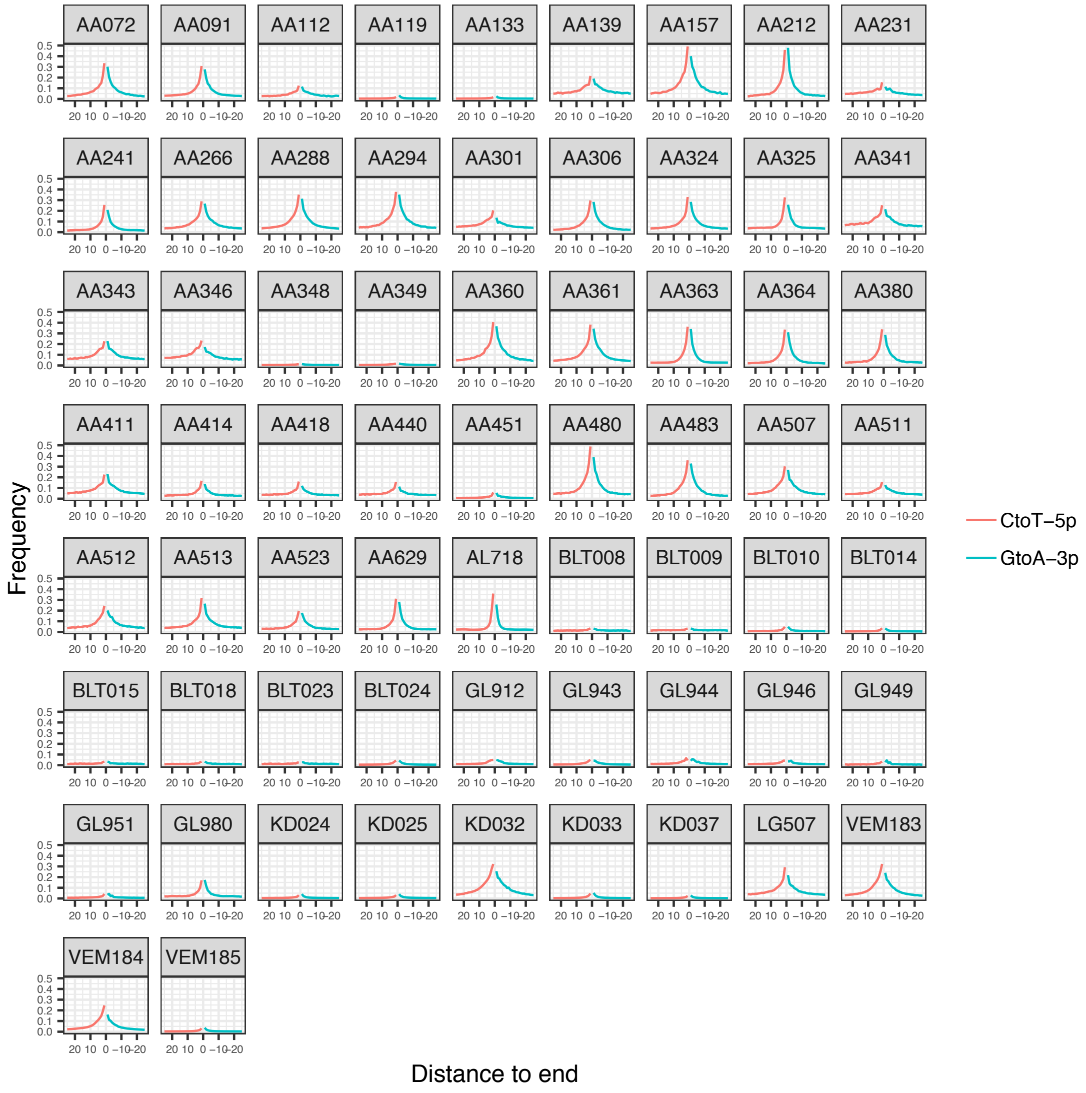

Fig. S1.

Per library C to T (red) and G to A (blue) frequency of mis-incorporation at 3’ and 5’ end of read for samples used in nuclear genome analyses. Libraries starting with BLT, as well as KD033 KD037, KD025, VEM185, AA119, and AA133 were all USER treated.

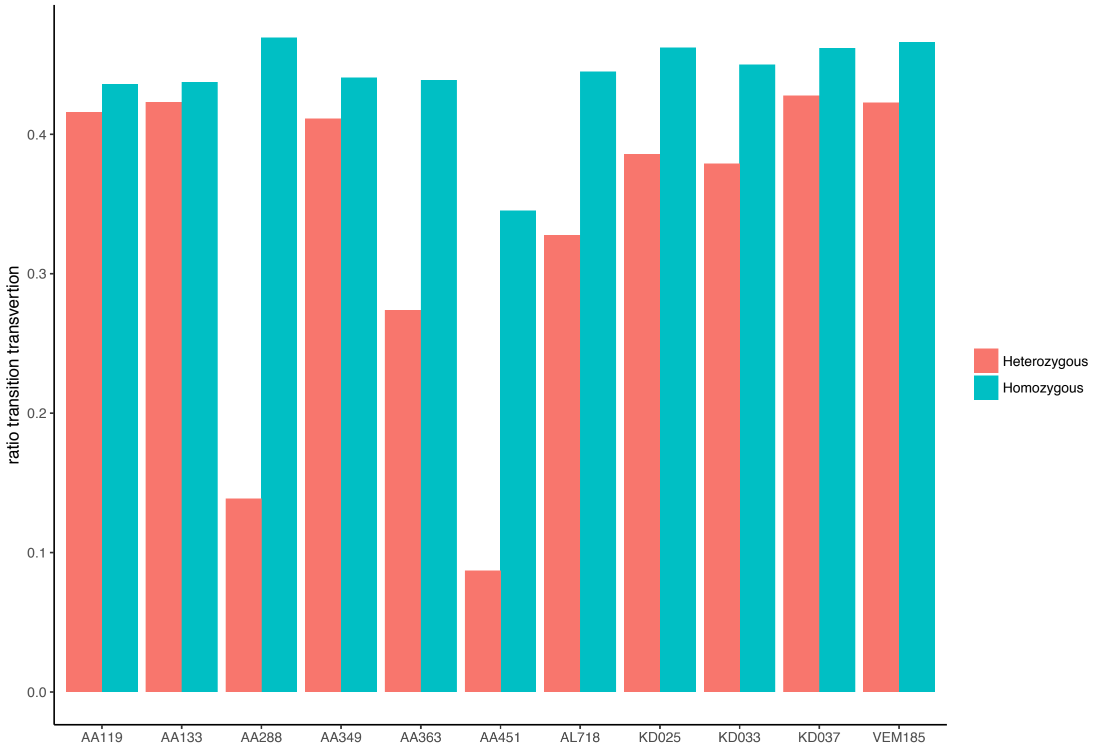

Fig. S2.
Ratio of transversion/transition at heterozygous sites for genome with >3x coverage.

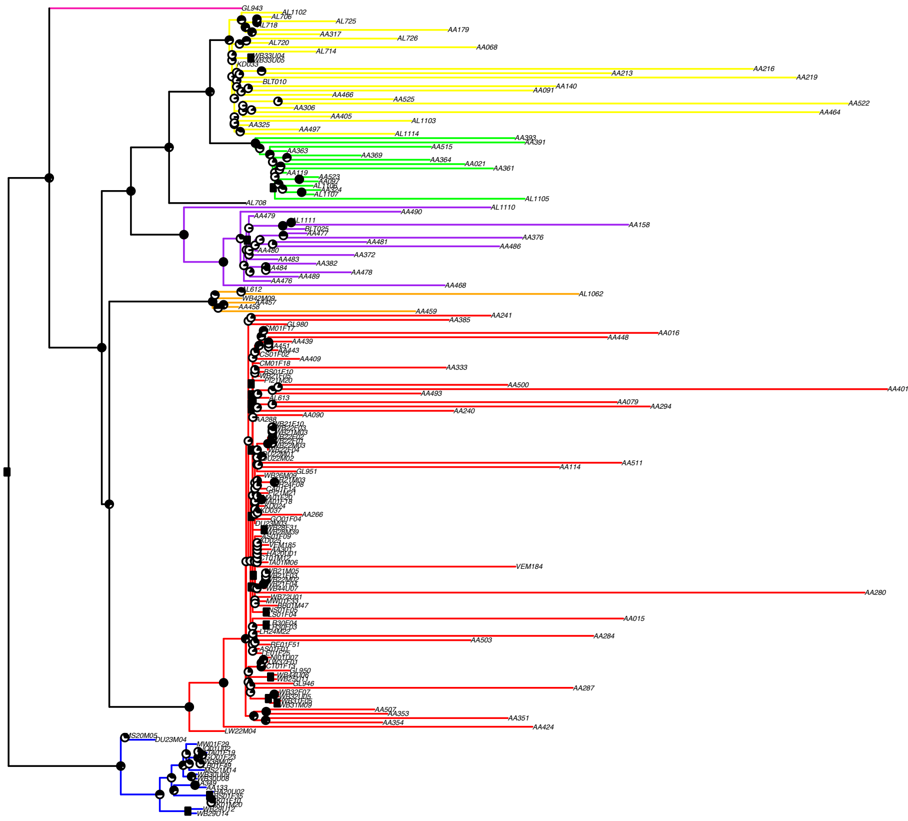

Fig. S3.
Maximum likelihood tree of 188 mitogenomes including both ancient and modern sequences. All ancient ancient genomes were at least 2x coverage. This tree depicts the relationship between the major haplogroup used in this study: As (blue), Y1 (yellow), Y2 (purple), ArmT (green), Eu (red), Italy (orange) and NE1 in pink. Black circle at each node represent bootstrap support (e.g. full black circle = 100 bootstrap support).

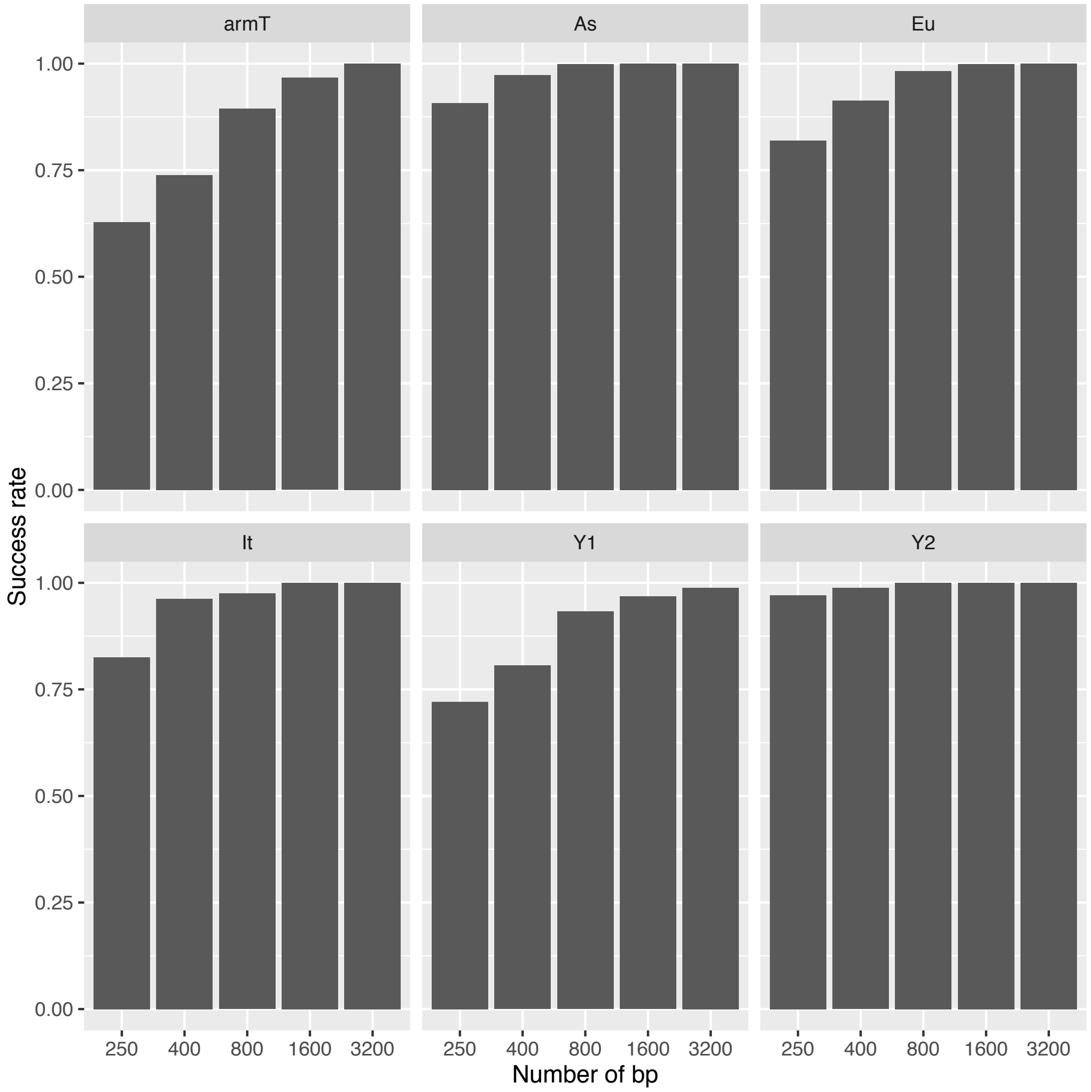

Fig. S4.

Success rate of our method to assign low coverage genome to specific haplogroup for different level of coverage (Number of base pair covered in the reference mitogenome). This was obtain by subsampling genome with known haplogroup.

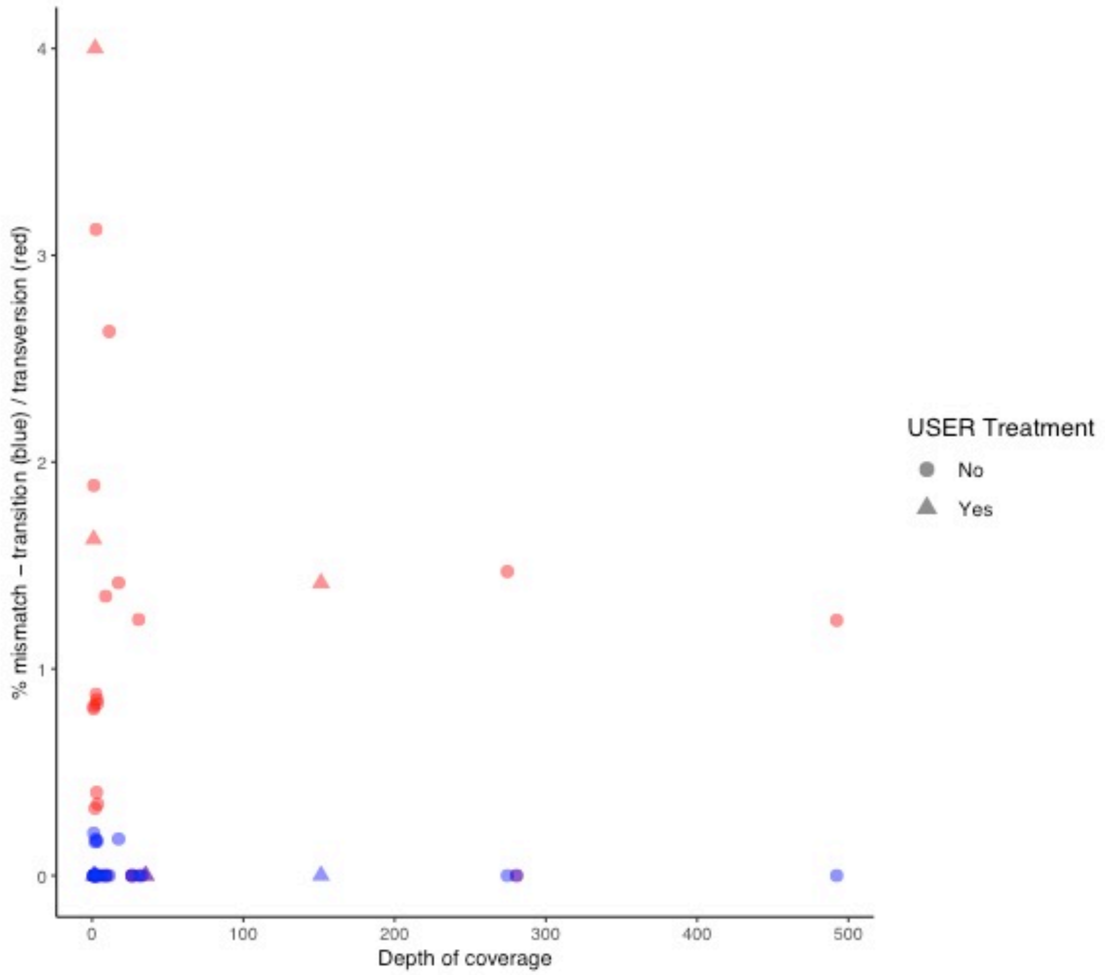

Fig. S5.
This plot represents the % of mismatch between sanger sequenced and NGS (depth of coverage on the x-axis) mitochondrial control haplotypes in 29 samples for which we possessed both data.

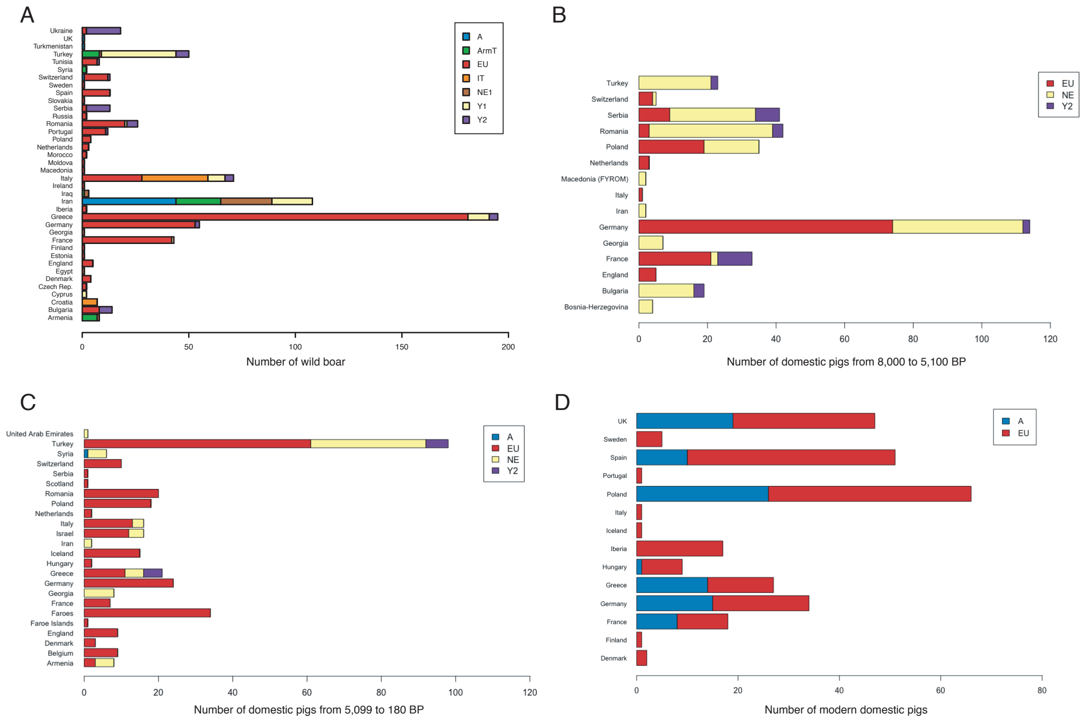

Fig. S6.
The bar plots show the number of domestic pig/wild boar samples that possess that possess As (blue), Y1 (yellow), Eu (red), Y2 (purple), ArmT (green), NE1 (brown), and Italy (orange) haplotypes divided different categories as seen in **Fig. 1** and **Fig. S7**. **A.** modern wild boar. **B.** domestic pigs between 8,000 BP and 5,100 BP. **C.** domestic pigs between 5,099 and 180 BP **D.** modern domestic pigs.

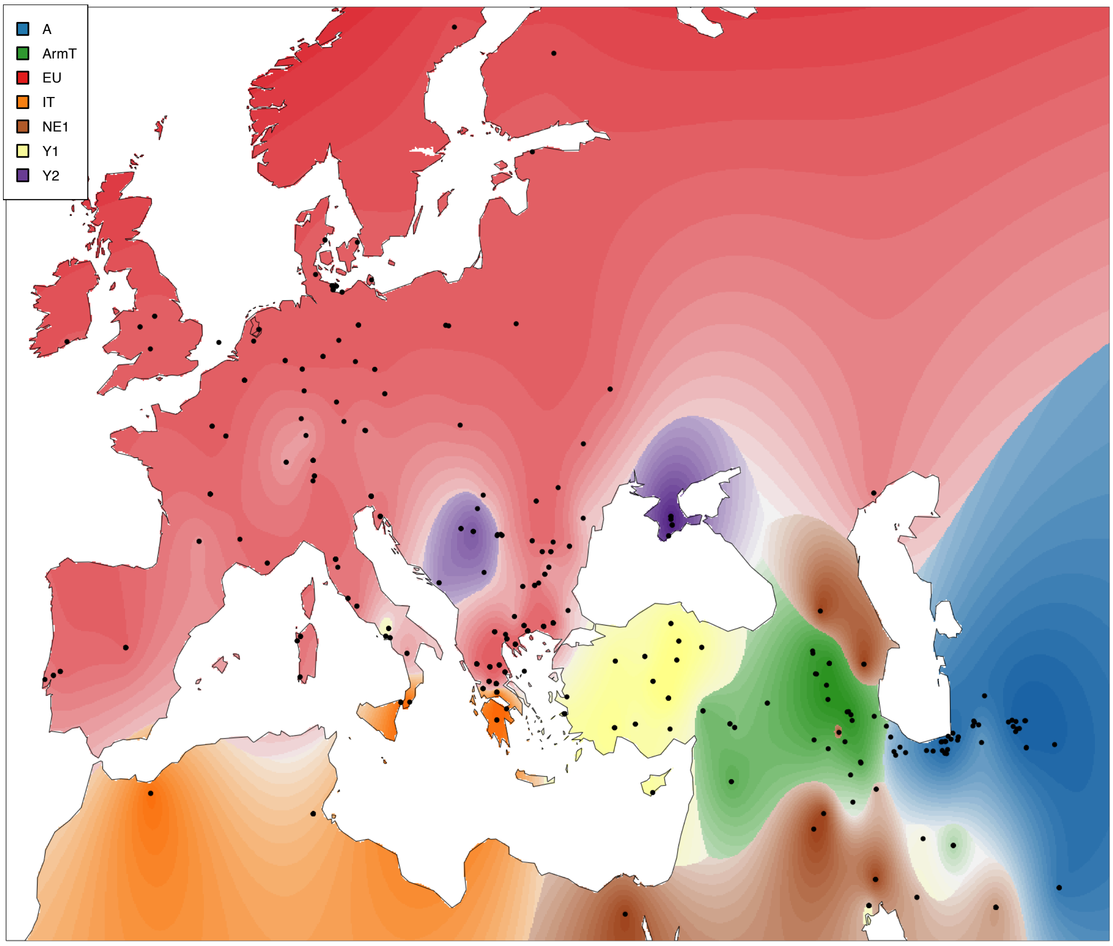

Fig. S7.
The black dots on the map represent locations of 696 modern and ancient wild boar that possess As (blue), Y1 (yellow), Eu (red), Y2 (purple), ArmT (green), NE1 (brown), and Italy (orange) haplotypes. The haplotype assignments were used to interpolate the underlying color distribution which demonstrates the biogeographical boundaries of these haplotypes. The bar plots that show the number of wild boar samples per country are in **Fig. S6A.**

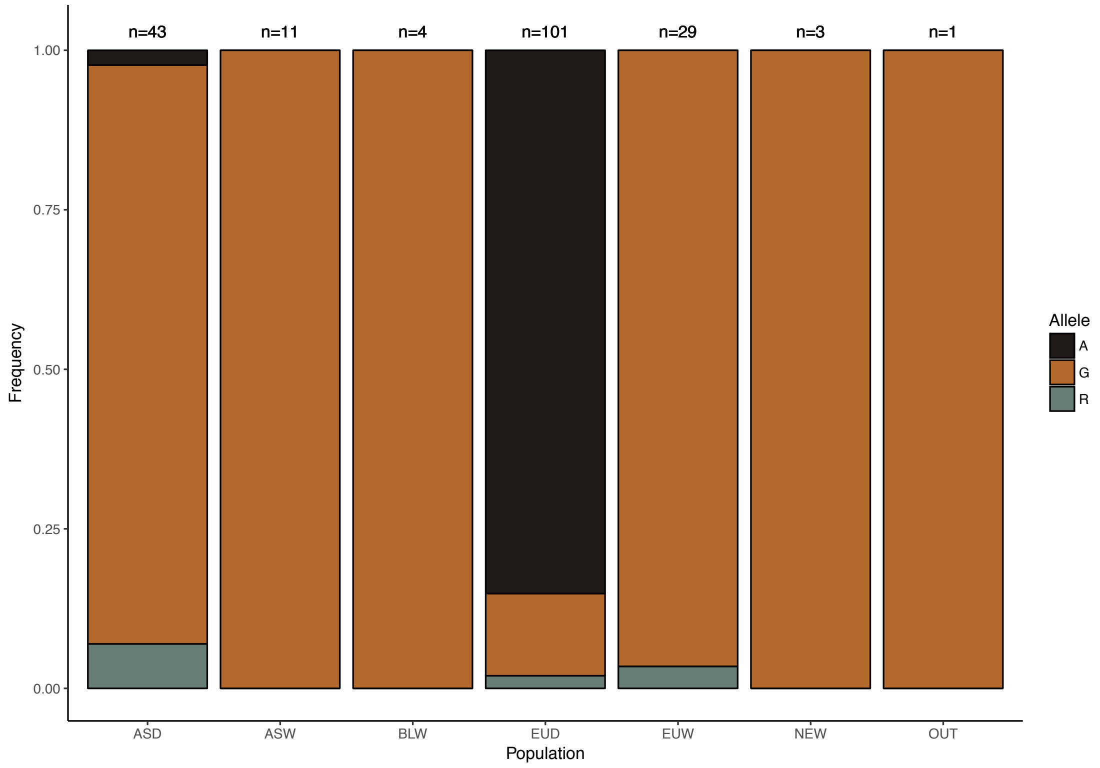

Fig. S8.

Frequency of three possible genotypes in the MC1R gene. G=ancestral allele (camouflage coat colour) and A=derived allele (black coat colour). Population codes: ASD=Asian domestic, ASW=Asian wild BLW=Balkan wild, EUD=European domestic, EUW=European wild (including Italian), NEW=Near Eastern wild, OUT=outgroup (S. verrucosus).

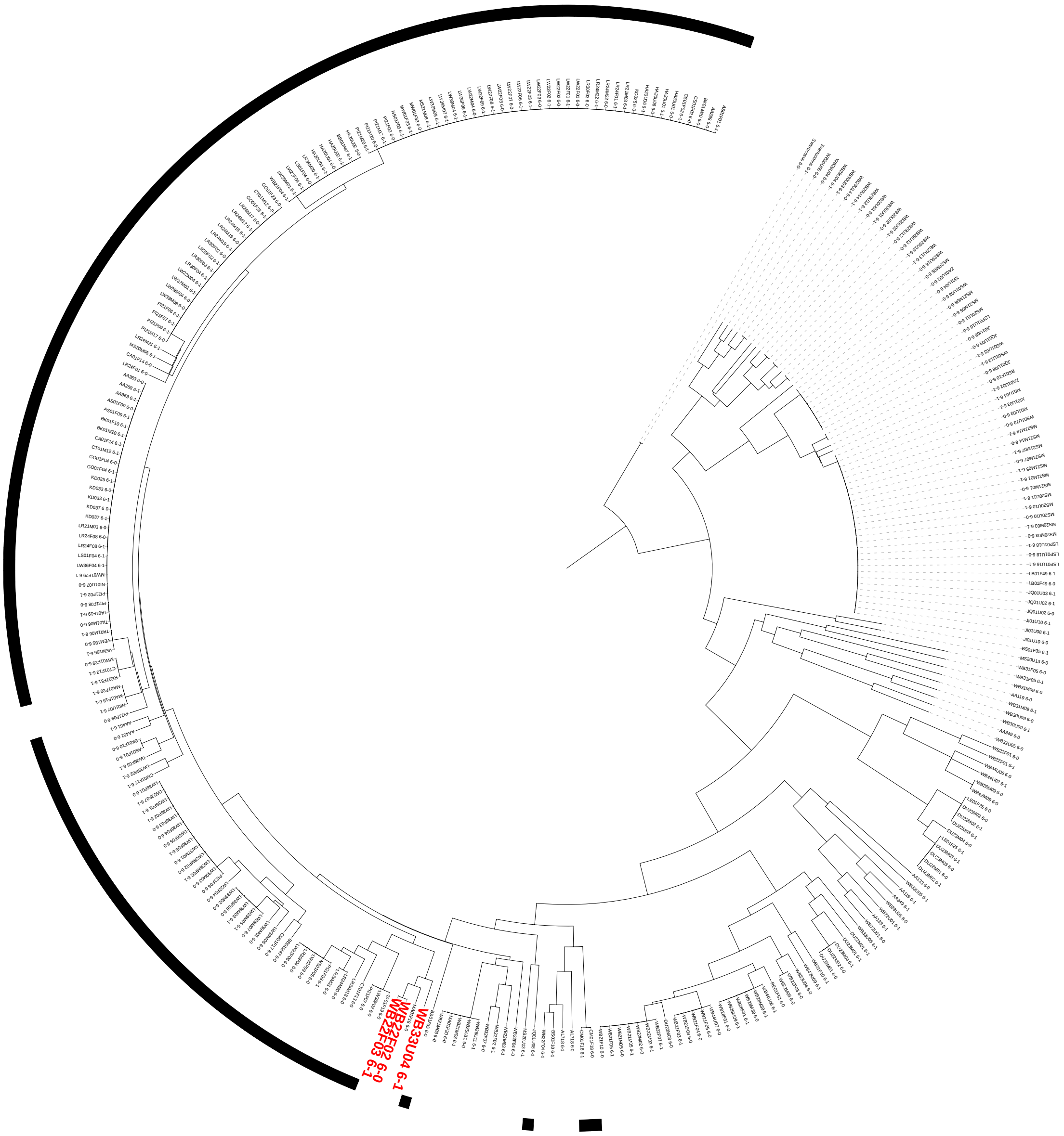

Fig. S9.
Ultrametric tree (UPGMA) based on a 50kb haplotypes around the *MC1R* gene. Black colour stripe represent the samples that possess the A derived allele (black coat colour). Red labels represent the three wild boar haplotypes that are closest to the monophyletic group that possess the derived allele.

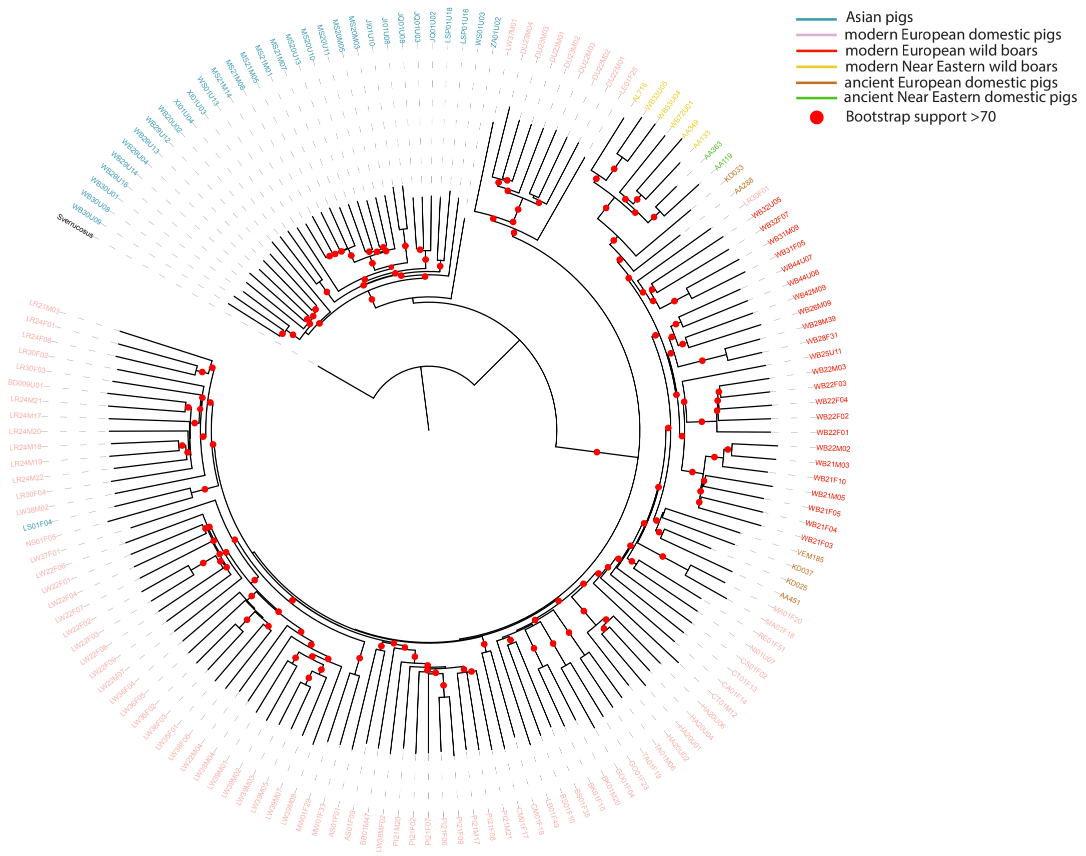

Fig. S10.
Neighbour joining tree based on high genome coverage ancient samples. Node labels represent bootstrap support. This figure shows that KD033 and AA288, both of which have substantial Near East ancestry (see Fig. S15; Fig. 2B) cluster with Near Eastern wild boars.

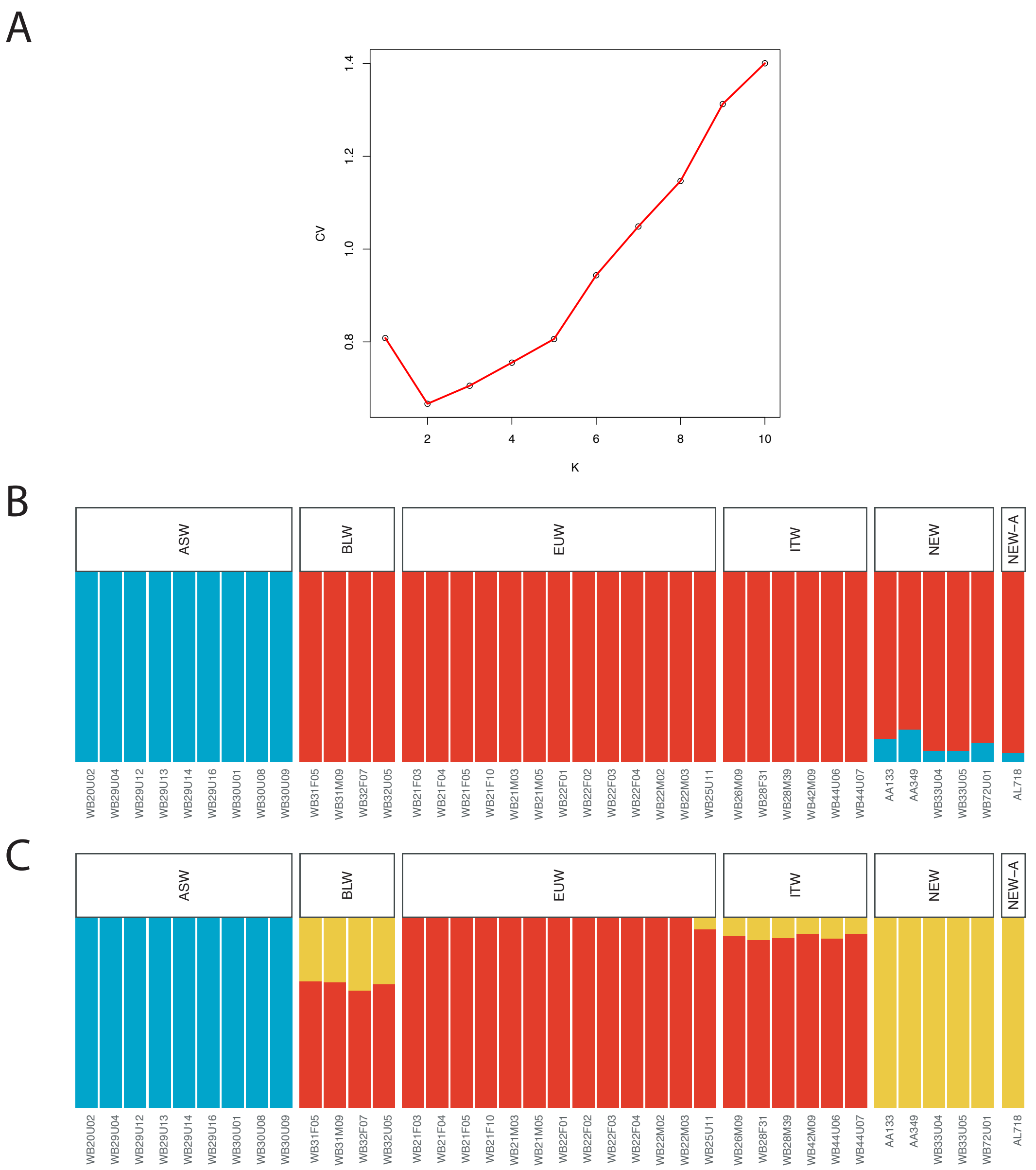

Fig. S11.
Results of ADMIXTURE analysis for wild boar genomes. **A.** Cross validation for different K values. **B.** Results for K=2. **C.** Results for K=3. ASW=Asian wild, BLW=Balkan wild, EUW=European wild, ITW= Italian wild, ITW-A= Italian wild ancient, NEW=Near Eastern wild, NEW-A=Near Eastern wild ancient.

A

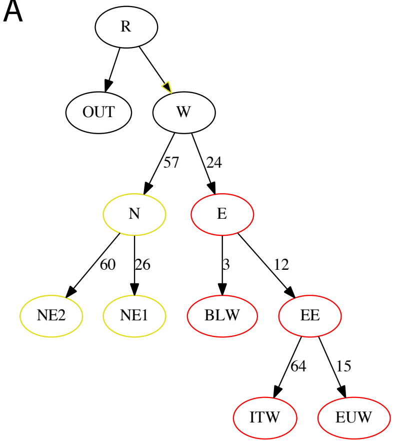

outliers:

f4				fitted	estim	diff	std. err	Z score
OUT	NE2	ITW	BLW	0.000000	0.017129	0.017129	0.004127	4.150
OUT	NE2	EUW	BLW	0.000000	0.014034	0.014034	0.003612	3.885
OUT	ITW	NE2	NE1	0.000000	0.017525	0.017525	0.004425	3.961
OUT	ITW	NE2	BLW	0.024235	0.045152	0.020917	0.005386	3.883
OUT	EUW	NE2	EUW	0.050777	0.067098	0.016321	0.005439	3.001
OUT	NE1	EUW	BLW	0.000000	0.017049	0.017049	0.002950	5.780
NE2	EUW	ITW	BLW	-0.011806	-0.028878	-0.017072	0.004931	-3.462
NE2	EUW	EUW	BLW	-0.026543	-0.044327	-0.017784	0.005668	-3.138
NE2	NE1	ITW	EUW	0.000000	-0.010708	-0.010708	0.003277	-3.268
ITW	NE1	EUW	BLW	0.011806	0.023546	0.011740	0.003654	3.213
EUW	NE1	EUW	BLW	0.026543	0.047342	0.020799	0.005160	4.031

B

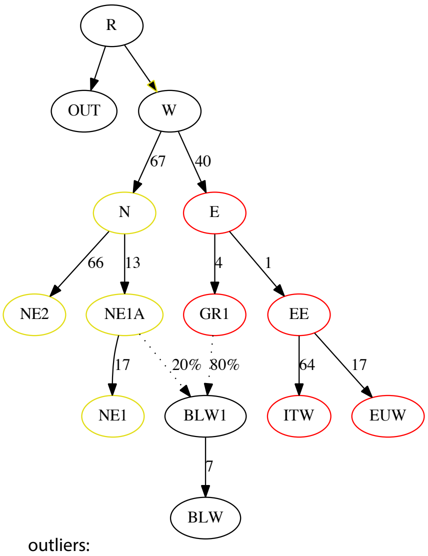

outliers:

f4				fitted	estim	diff	std. err	Z score
OUT	ITW	NE2	NE1	0.000000	0.017525	0.017525	0.004425	3.961
NE2	NE1	ITW	EUW	0.000000	-0.010708	-0.010708	0.003277	-3.268

C

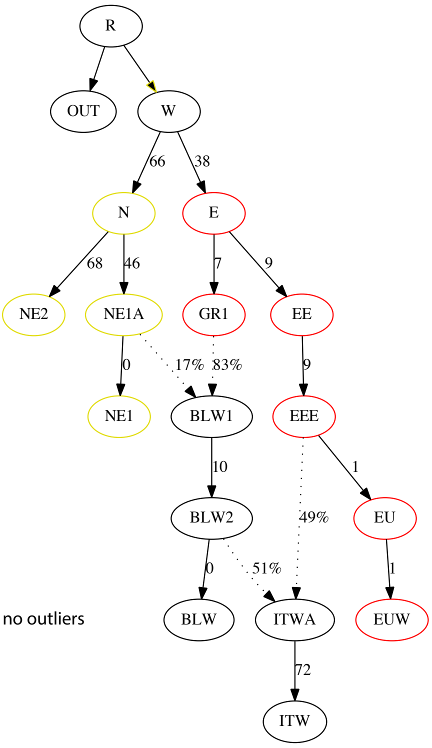

no outliers

Fig. S12.
Results of *Qpgraph* analysis on wild boars genome. In this analysis population were split into NE2 (Iran and Armenia), NE1 (Turkey and Samos), BLW (Balkan), ITW (Italy and Switzerland), EUW (rest of Europe), OUT (*S. verrucosus*). **A.** Model with no gene-flow - this model left 11 outliers. **B.** Model involving gene-flow from Turkish (NE1) wild boars into Balkan wild boars (BLW) - this analysis left two outliers. **C.** Model involving gene-flow from Turkish (NE1) wild boars into Balkan wild boars (BLW) and gene-flow from Balkan into Italy (ITW) - this analysis left no outliers.

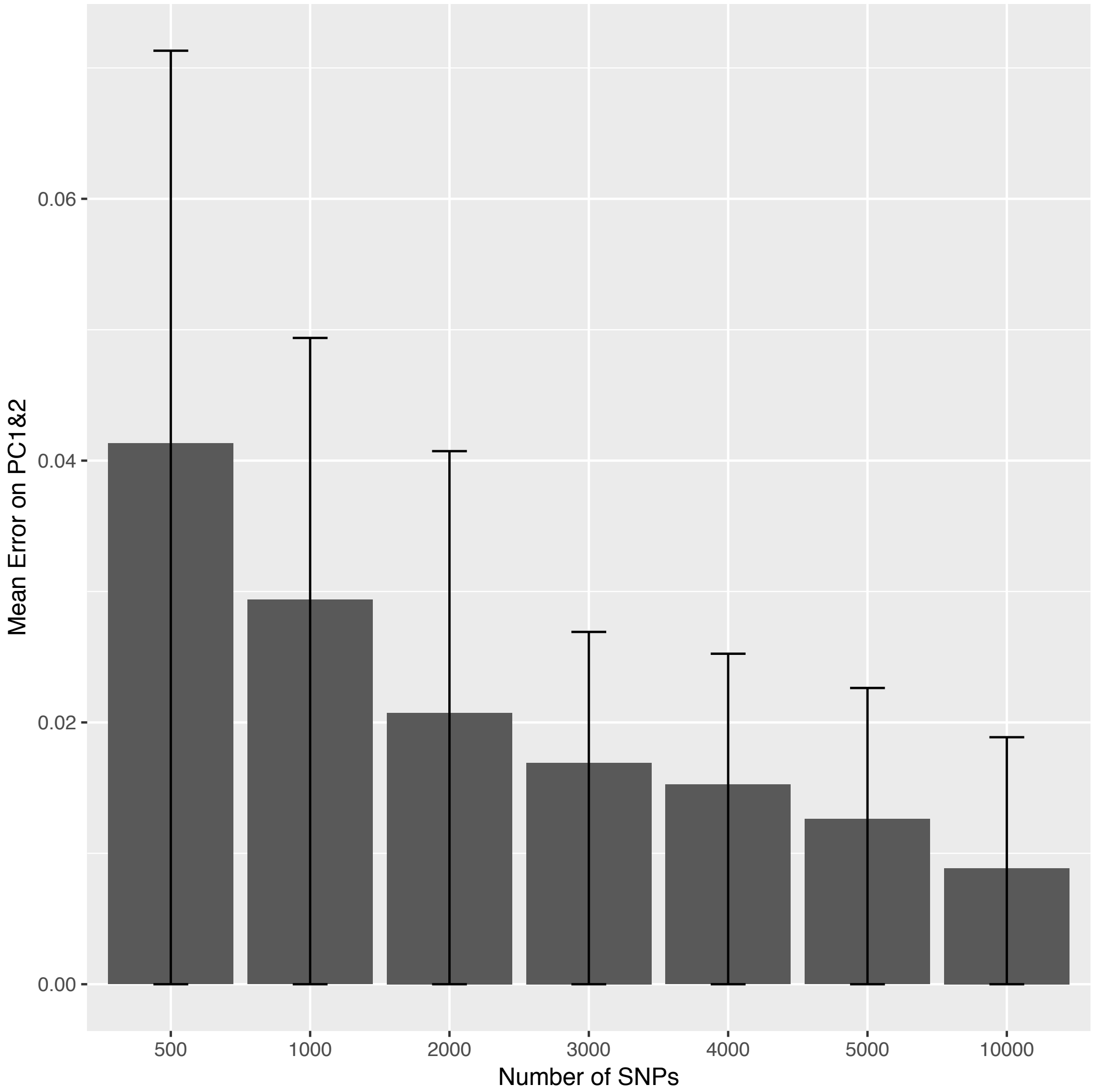

Fig. S13.
Mean error rate on PC1&2 for low coverage data with different levels of SNP coverage.

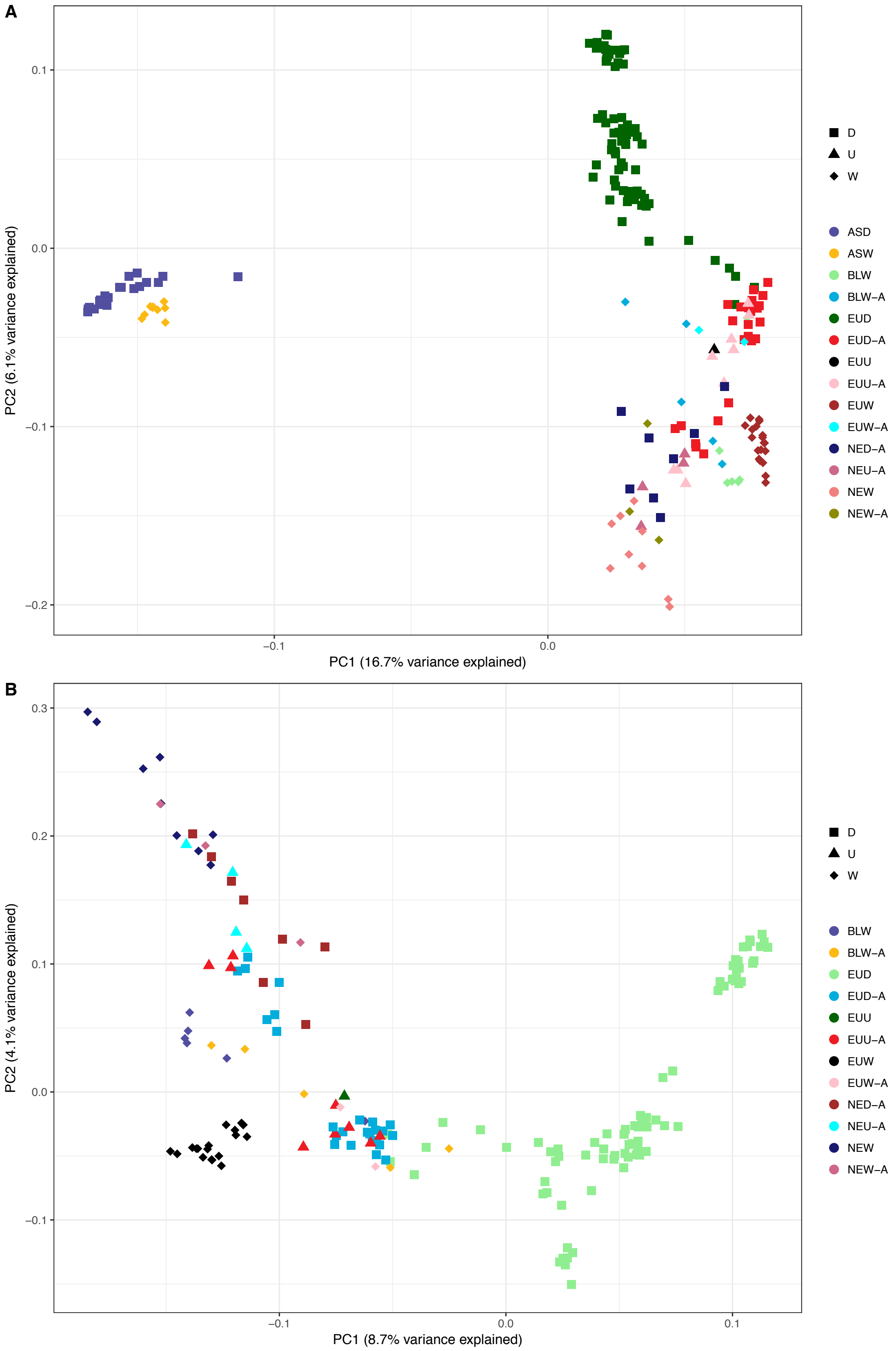

Fig. S14.
PCA including low coverage data (projected). **A.** PCA including Asian wild and domestic samples. **B.** PCA without Asian wild samples. Samples are split into multiple categories. Symbols: D=Domestic, U=Unknown status, W=Wild. Population code: ASD=Asian domestic, ASW=Asian wild BLW=Balkan wild, BLW-A=Balkan

wild ancient, EUD=European domestic, EUD-A= European domestic ancient, EUU-A=European unknown (either wild or domestic) ancient, EUW=European wild (including Italian), EUW-A=European wild (including Italian), NED-A= Near Eastern domestic ancient, NEU= Near Eastern unknown (either wild or domestic) ancient, NEW=Near Eastern wild, NEW-A=Near Eastern wild ancient.

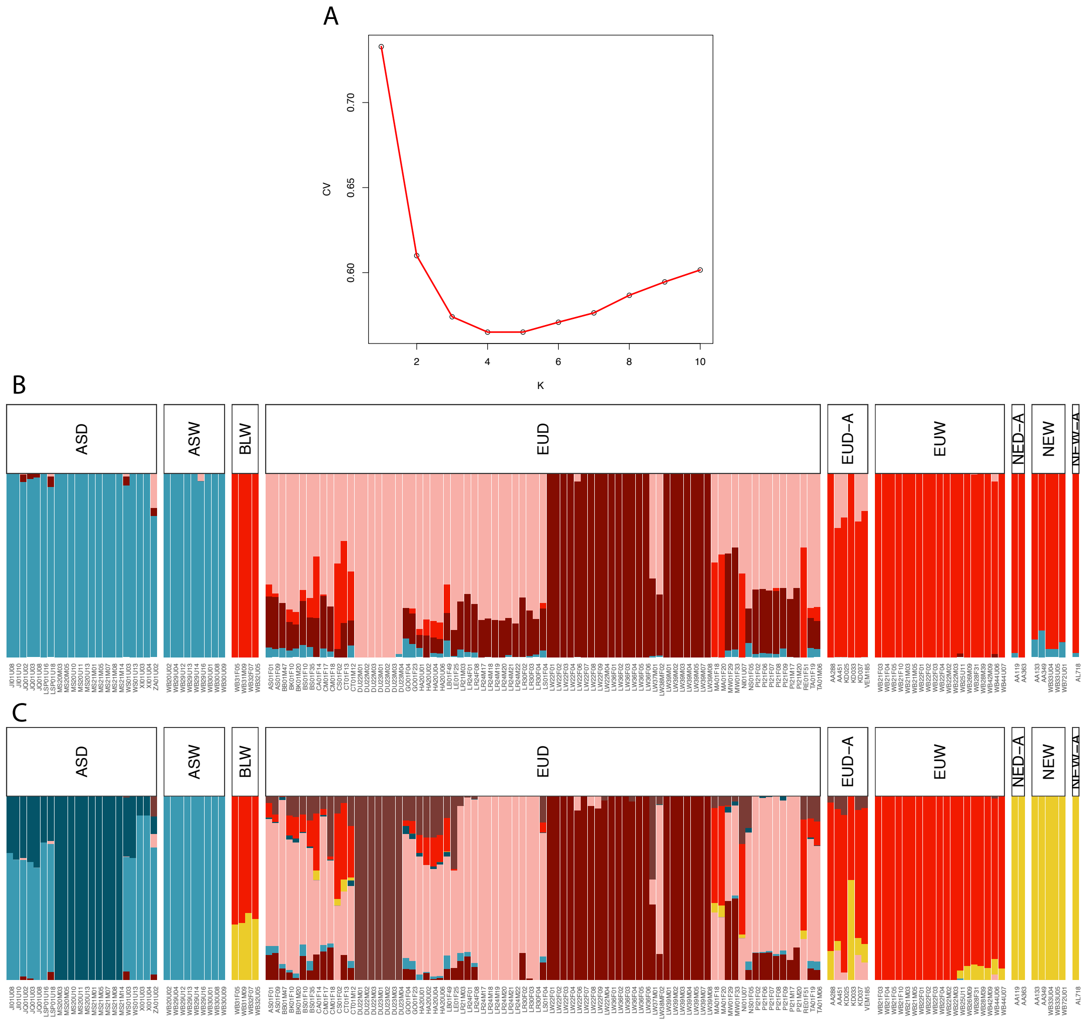

Fig. S15.
Results of ADMIXTURE analysis including all modern genomes and all high coverage ancient genome wild boar genomes. **A.** Cross validation for different K values. **B.** Results for K=4. **C.** Results for K=7. ASD=Asian domestic, ASW=Asian wild BLW=Balkan wild, BLW-A=Balkan wild ancient, EUD=European domestic, EUD-A= European domestic ancient, EUU-A=European unknown (either wild or domestic) ancient, EUW=European wild (including Italian), EUW-A=European wild (including Italian), NED-A= Near Eastern domestic ancient, NEU= Near Eastern unknown (either wild or domestic) ancient, NEW=Near Eastern wild, NEW-A=Near Eastern wild ancient.

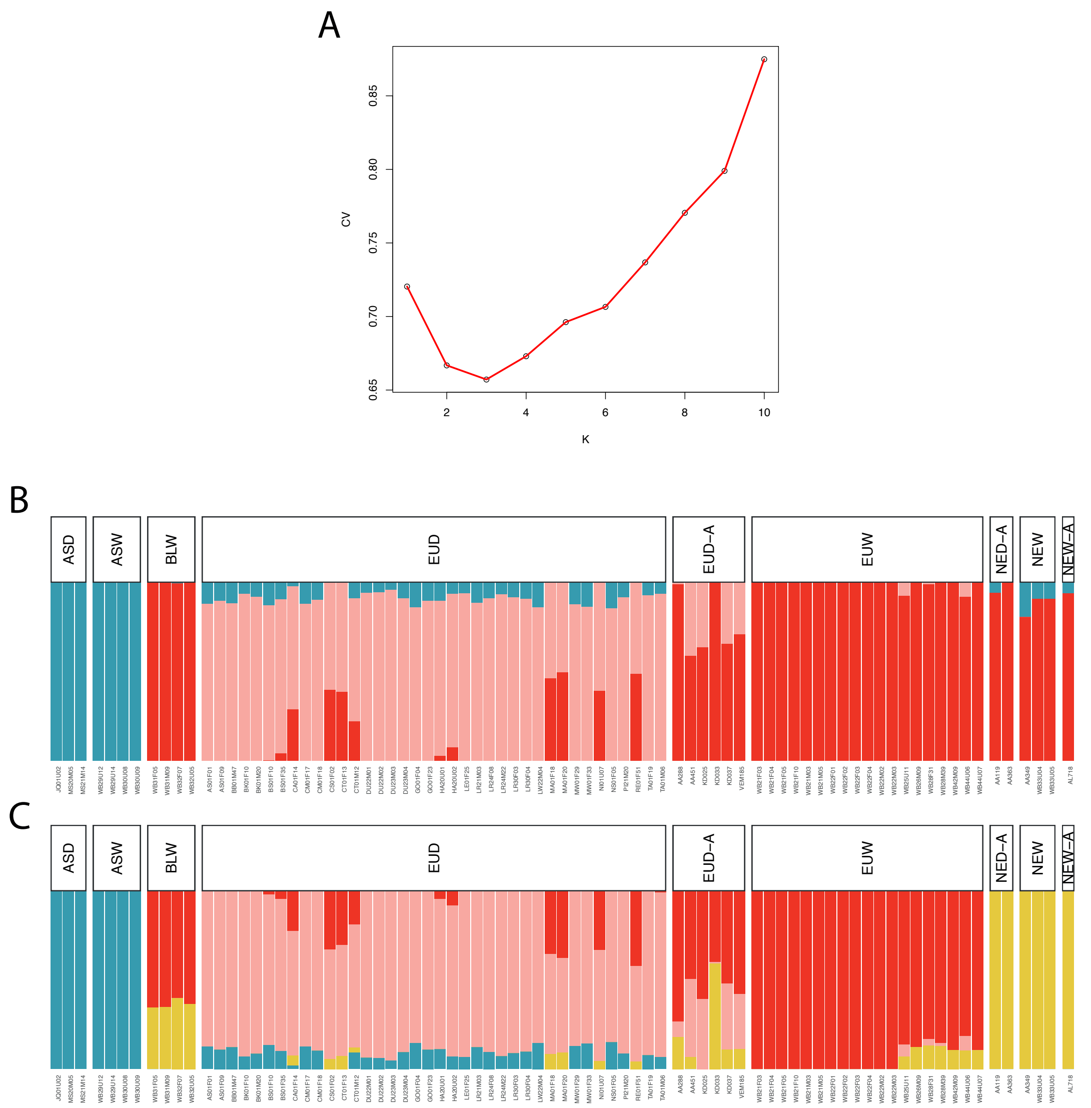

Fig. S16.
Results of ADMIXTURE analysis with only up to two genomes per modern breed. **A.** Cross validation for different K values. **B.** Results for K=3. **C.** Results for K=4. ASD=Asian domestic, ASW=Asian wild BLW=Balkan wild, BLW-A=Balkan wild ancient, EUD=European domestic, EUD-A= European domestic ancient, EUU-A=European unknown (either wild or domestic) ancient, EUW=European wild (including Italian), EUW-A=European wild (including Italian), NED-A= Near Eastern domestic ancient, NEU= Near Eastern unknown (either wild or domestic) ancient, NEW=Near Eastern wild, NEW-A=Near Eastern wild ancient.

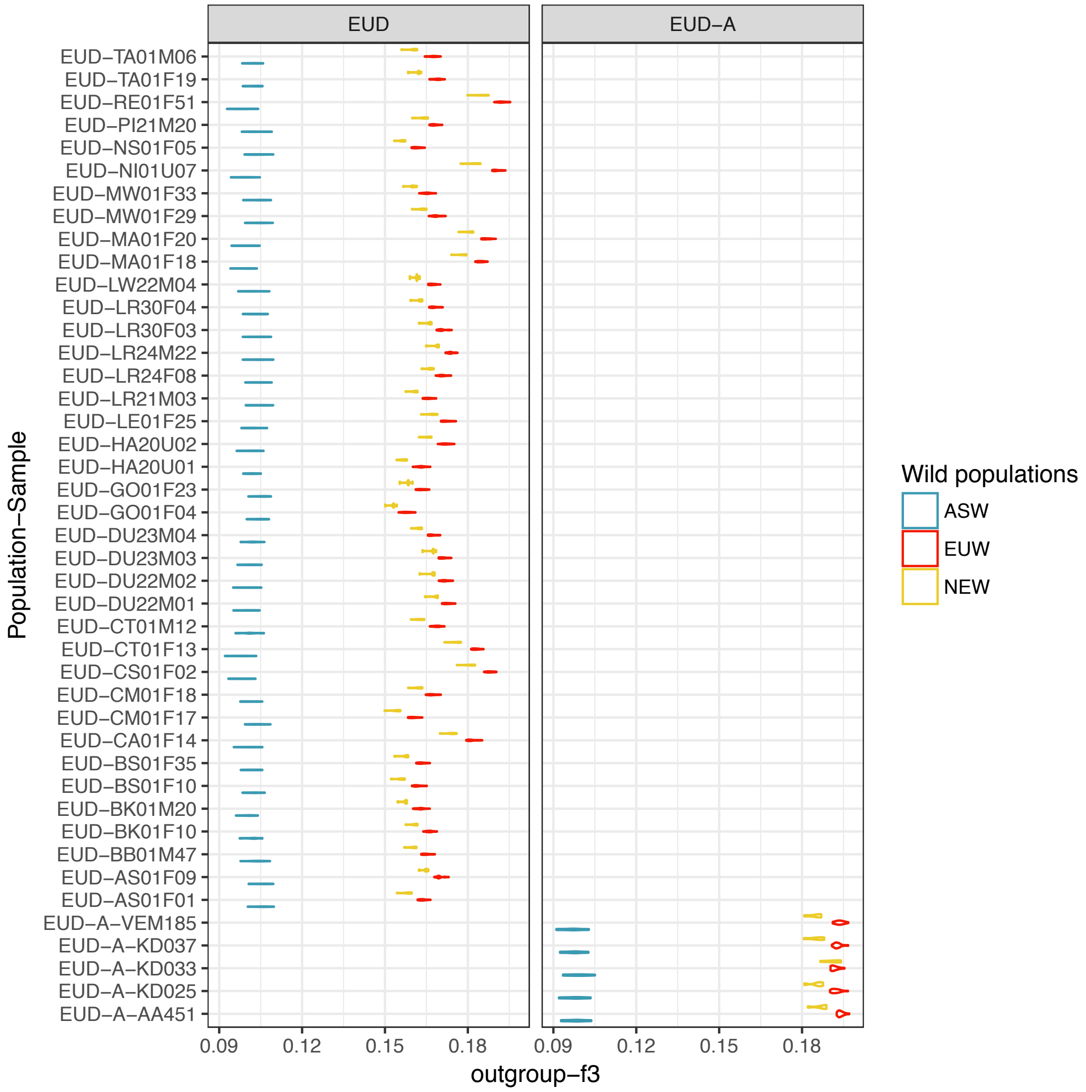

Fig. S17.

Violin plot representing the distribution of outgroup-f3 statistics of the form (*S. verrucosus*; [ASW/EUW/NEW], EUD) for each EUD genome.

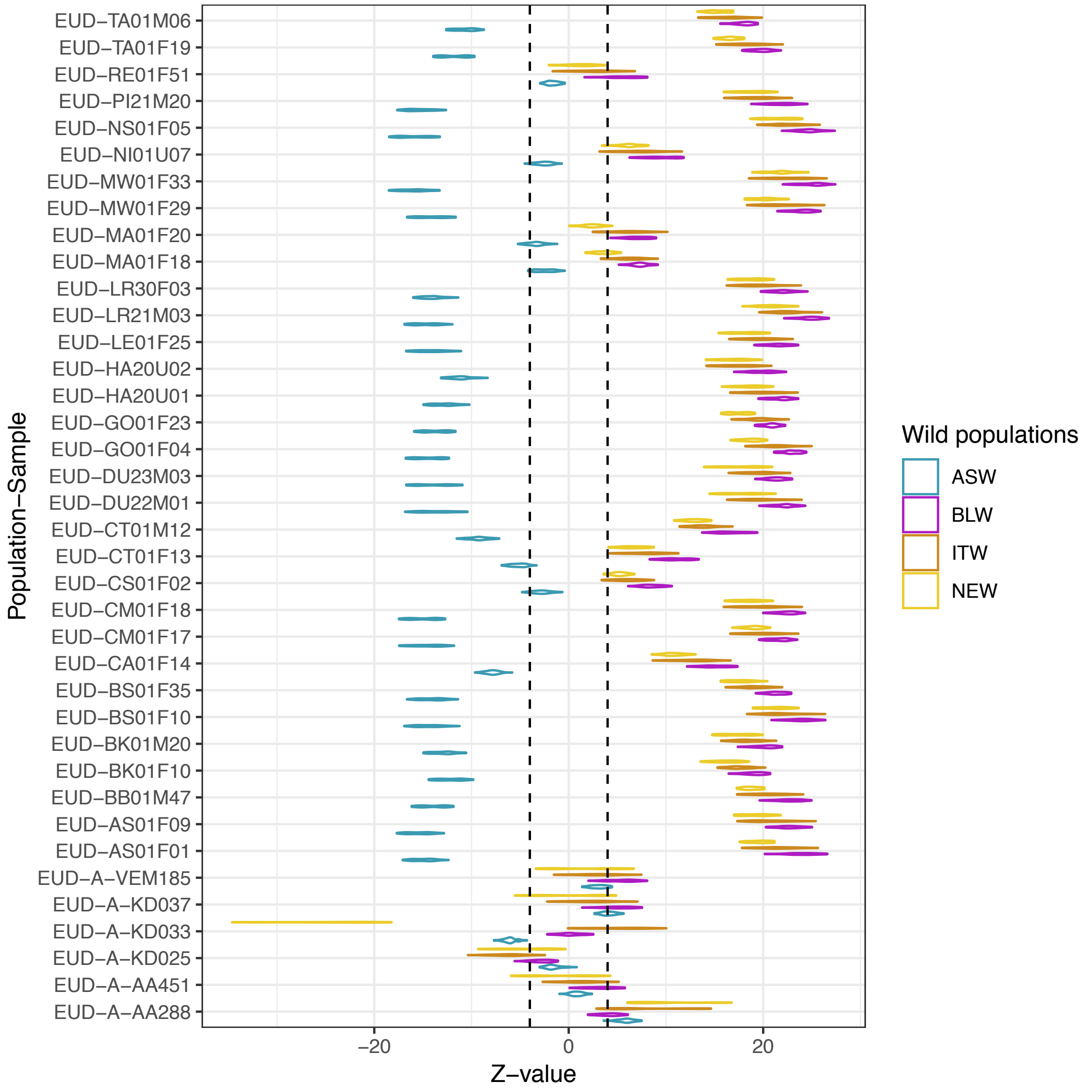

Fig. S18.
Violin plot representing the distribution of Dstatistics of the form D(S.verrucosus, [NEW/ITW/BLW/ASW]; EUD, EUW) for each EUD genome.

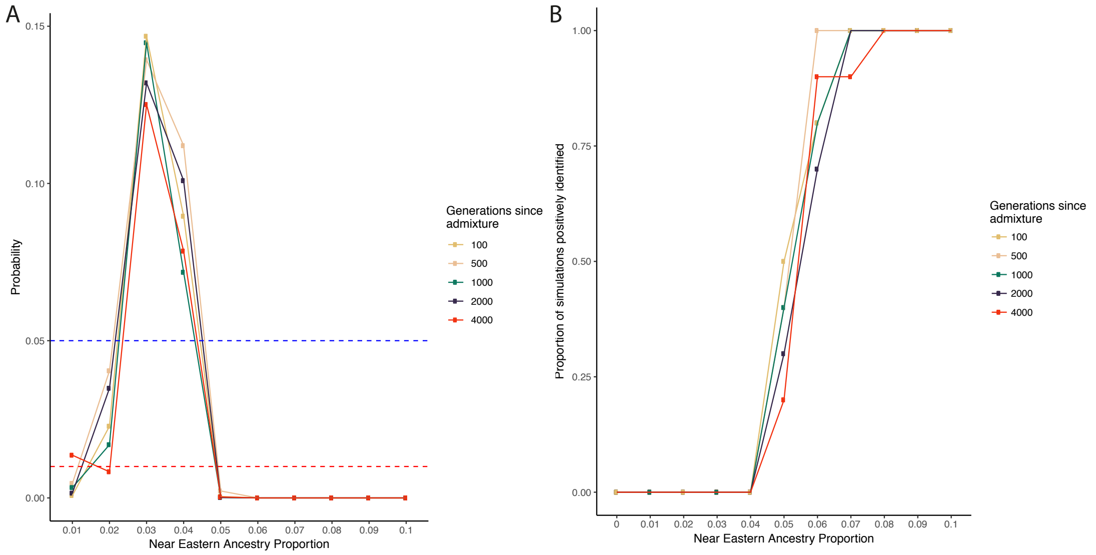

Figure S19.
A. Graph depicting the results of a simulation of European pig genomes using different timeframes of admixture with European wild boar, and different levels of Near Eastern ancestry (x-axis). These simulated genomes were analyzed using ADMIXTURE; the y-axis represents the probability (computed using a binomial distribution) of observing 8 out of 85 European domestic genomes with detectable Near Eastern ancestry (y-axis). **B.** Proportion of simulations that were (out of 10 replicates) for which *GLOBETROTTER* identified NEW ancestry in EUD for different levels of ancestry fraction and time since admixture.

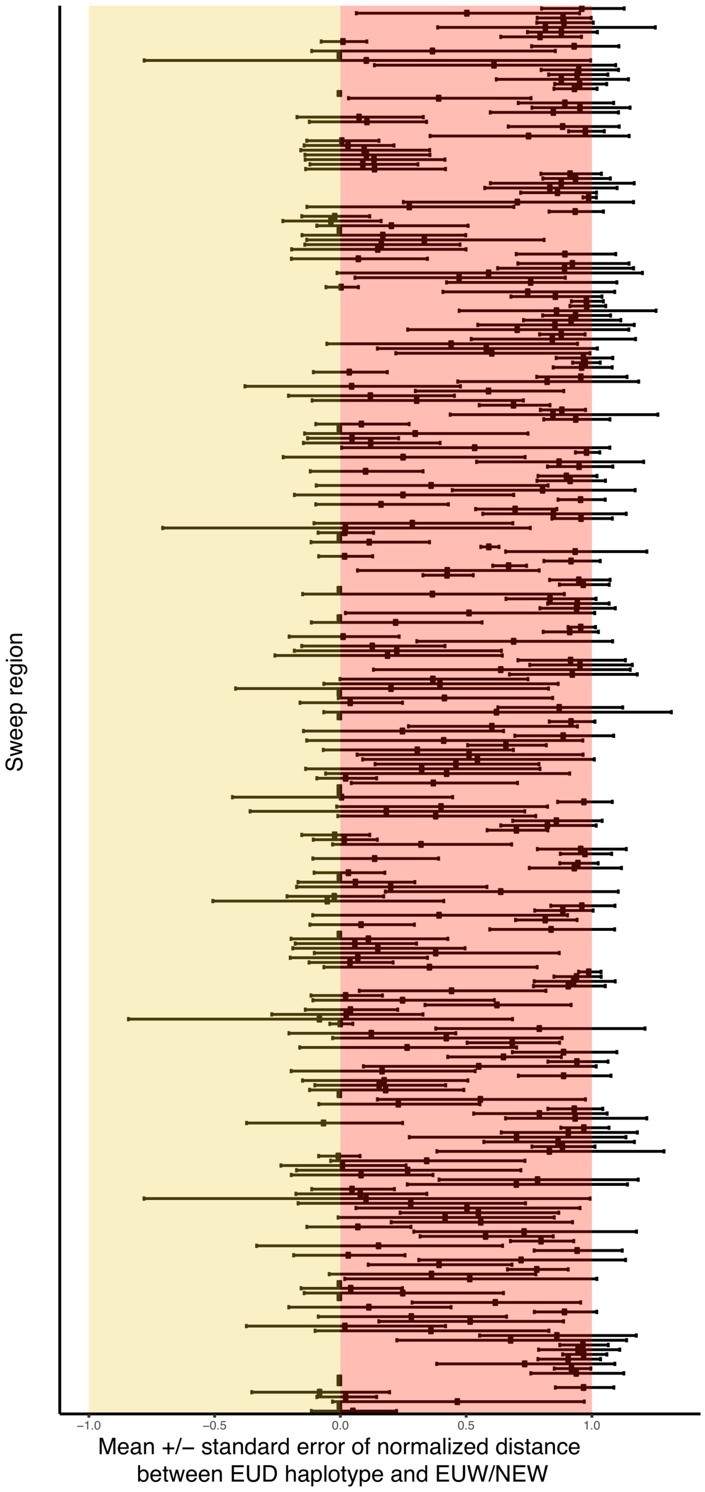

Figure S20.
A graph depicting the ancestry of haplotypes carried by domestic pigs in regions of the genome that underwent a selective sweep (338). Bars on the x-axis represent the mean and standard deviation of the normalized difference between the nucleotide distance of haplotypes found in domestic pigs to the closest European and Near Eastern wild boar haplotype. Positive and negative values indicate that, on average, haplotypes possessed by domestic pigs in a sweep region (y-axis) are closest to haplotypes found in European wild and Near East boars respectively.

A

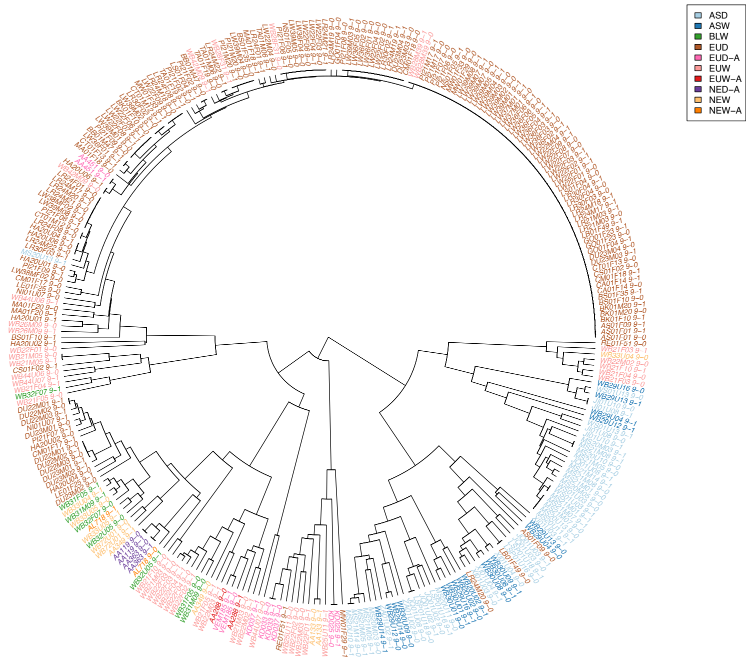

B

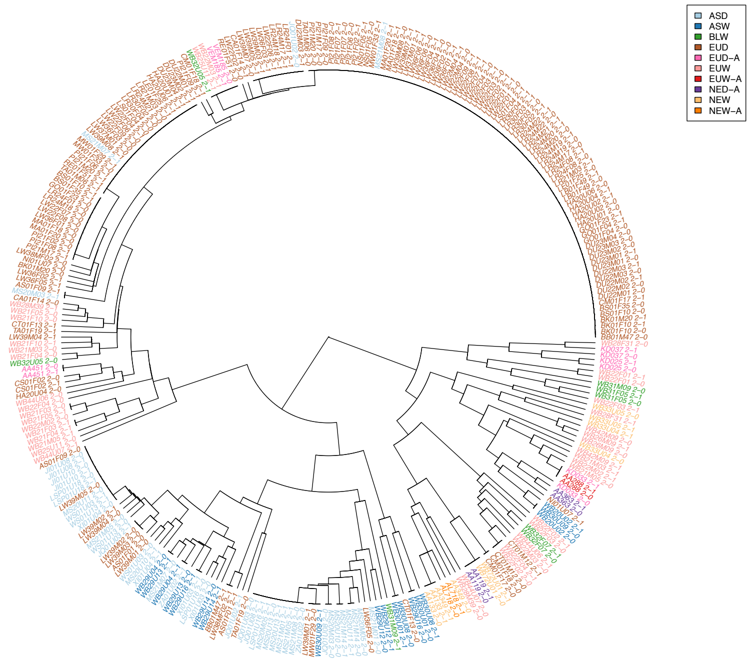

Fig. S21:
Ultrametric tree (UPGMA) in two regions detected as selective sweeps in European Domestic pigs (*335*). This figure shows a clear reduction of diversity in EUD in these specific regions - potentially due to a selective sweep. In both cases the selected haplotype in EUD appears to be much more closely related to EUW than NEW. **A** chromosome 2:73626576-74003521 **B** chromosome 9:109689664-109911665. Population codes: ASD=Asian domestic, ASW=Asian wild BLW=Balkan wild, BLW-A=Balkan wild ancient, EUD=European domestic, EUD-A= European

domestic ancient, EUU-A=European unknown (either wild or domestic) ancient, EUW=European wild (including Italian), EUW-A=European wild (including Italian), NED-A= Near Eastern domestic ancient, NEU= Near Eastern unknown (either wild or domestic) ancient, NEW=Near Eastern wild, NEW-A=Near Eastern wild ancient.

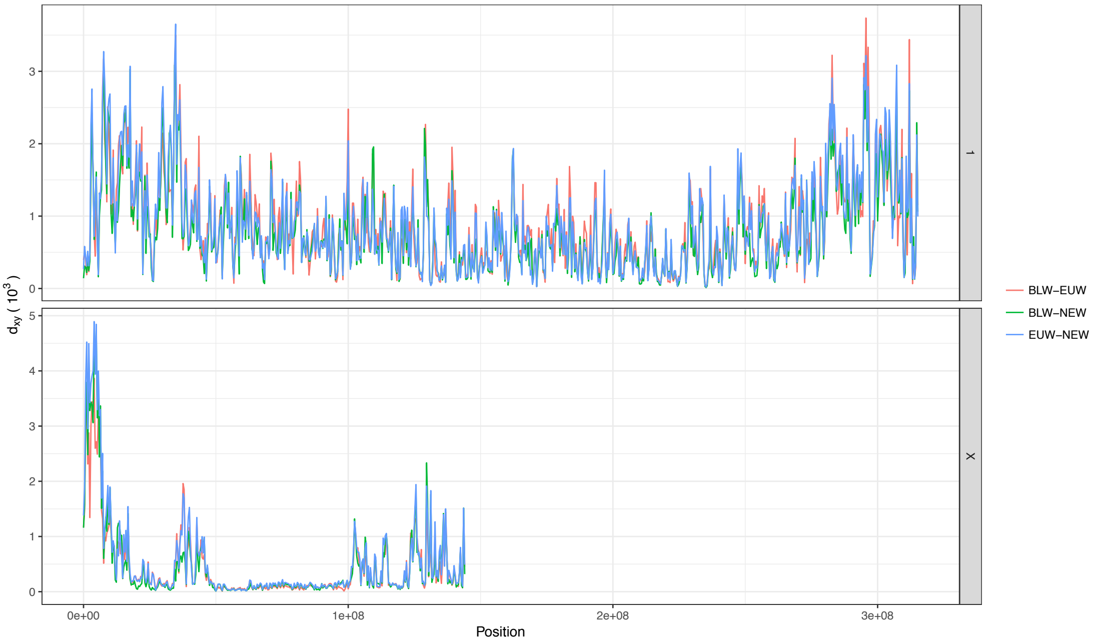

Fig. S22.
Dxy values plotted along chromosome 1 and X.

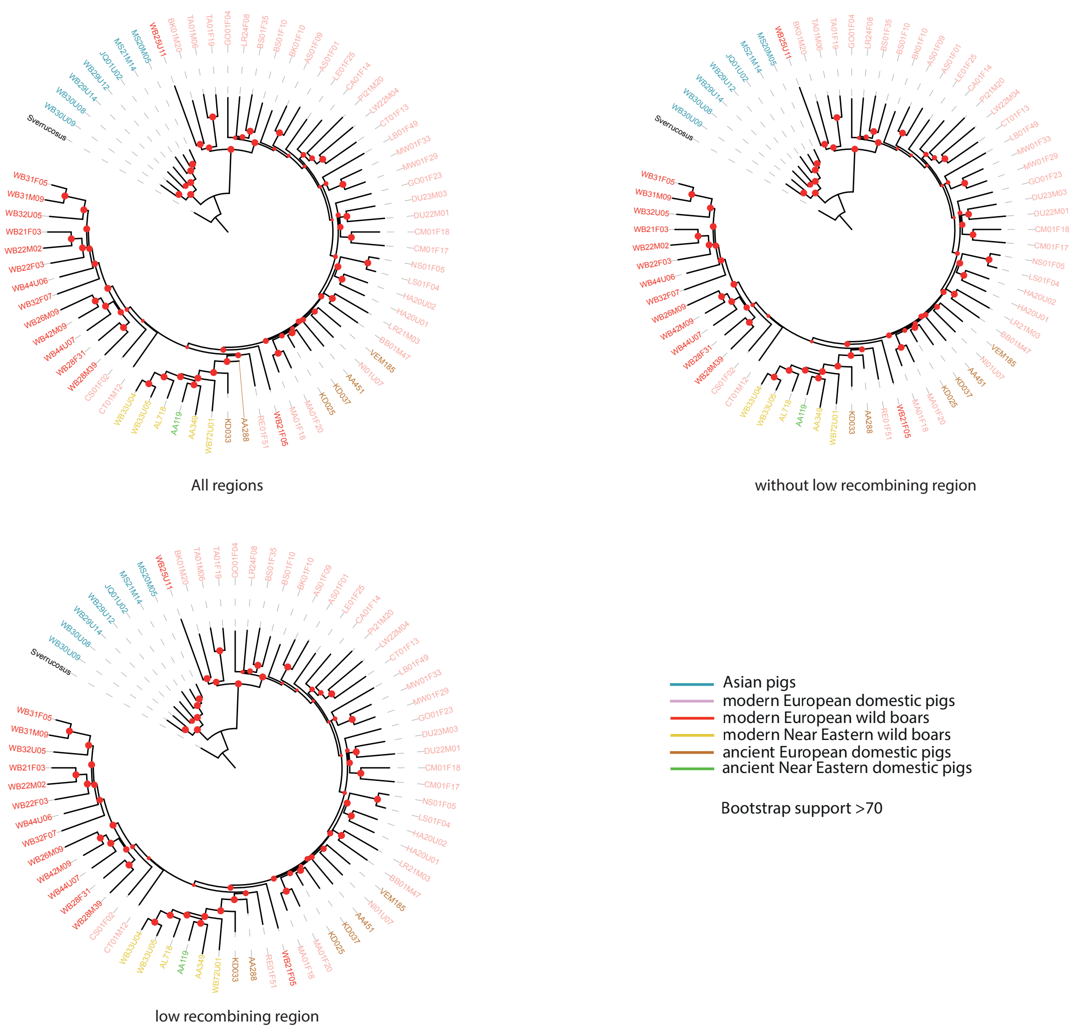

Fig. S23.
NJ tree for **A.** whole chromosome X **B.** only low recombining region of chromosome X **C.** without the low recombining region.

References

1. Benecke N, Bollongino R, Küßner M, Weber R (2006) Zur Datierung und Fauna des spätglazialen Schichtkomplexes am Abri Fuchskirche I bei Allendorf, Lkr. *Saalfeld-Rudolstadt Alt-Thüringen* 39:121–130.

2. Lechevallier M, et al. (1978) Abou Gosh et Beisamoun. Deux gisements du VII millénaire avant l'ère chrétienne en Israël. *Mémoires et Travaux du Centre de Recherches Préhistoriques Français de Jérusalem Jérusalem* 2.

3. Khalaily H, Marder O, ha-ʻatiḳot IR (2003) *The Neolithic site of Abu Ghosh: the 1995 excavations*.

4. Özgüç N (1966) EXCAVATIONS AT ACEMHOYtlK. *Anadolu (Anatolia)*. doi:10.1501/andl_0000000092.

5. Öztan A, Arbuckle BS (2001) Yılı Acemhöyük Kazıları. *Kazı Sonuçları Toplantısı* 24:1–39.

6. Arbuckle BS (2006) The Evolution of Sheep and Goat Pastoralism and Social Complexity in Central Anatolia. Dissertation (Harvard University).

7. Burenhult G (1997) *Ajvide och den moderna arkeologin* (Natur och Kultur).

8. Burenhult G, Brandt B (2002) The grave-field at Ajvide. *Remote Sensing. Applied Techniques for the Study of Cultural Resources and the Localization, Identification and Documentation of Sub-Surface Prehistoric Remains in Swedish Archaeology*, ed Burenhult G (Department of Archaeology, Stockholm University, Stockholm).

9. Burenhult G (1997) Säljägare och svinaherdar på Ajvide. *Ajvide Och Den Moderna Arkeologin. Natur Och Kultur*.

10. Gligor M (2009) *Aşezarea neolitică şi eneolitică de la Alba Iulia-Lumea Nouă în lumina noilor cercetări* (Ed. Mega).

11. Gligor M (2014) Începuturile eneoliticului timpuriu în Transilvania: o abordare Bayesiană [The beginning of Early Eneolithic in Transylvania: a Bayesian approach]. *Analele Banatului* 22:91–105.

12. Gligor M, McLeod K (2014) Disarticulation as a mortuary practice in Early Eneolithic Transylvania? A case study from Alba Iulia-Lumea Nouă. *Annales Universitatis Apulensis, Series Historica* 18(2).

13. Gligor M, Mcleod KKM (2015) Disposal of the dead. Uncommon mortuary practices from Alba Iulia-Lumea Nouă 2003 excavation. *Homines, Funera, Astra 2". Life Beyound Death in Ancient Times. Proceedings of the International Symposium on Funerary Anthropology. 23-26 September 2012, "1 Decembrie 1918" University (Alba Iulia, Romania)*, ed R. Kogălniceanu, M. Gligor, R. Curcă, S. Straton (Archaeopress, Oxford), pp 25–41.

14. Lundberg C, Gligor M PLACE OF DEATH AND PLACE OF REST: COMMINGLED HUMAN REMAINS FROM ALBA IULIA-LUMEA NOUĂ 2015 EARLY ENEOLITHIC FUNERARY DISCOVERY. *Annales Universitatis Apulensis, Series Historica* 19(2).

15. Szirmai K (2004) Feltárások és leletmentések az auxiliáris castellum vicusában Albertfalván. *Aqunicumi Füzetek* 10:116–122.

16. Bökönyi S (1974) *History of domestic mammals in Central and Eastern Europe* (Akademiai Kiado).

17. Papathanasiou A (2005) Health status of the Neolithic population of Alepotrypa Cave, Greece. *Am J Phys Anthropol* 126(4):377–390.

18. Papathanasopoulos GA, Katsipanou-Margeli V, Kourtesi-Filippaki G (2011) To Neolithiko Diro. *Spilaio Alepotrypa* (Ekd. Oikos Melissa Athina).

19. Moser B (1986) Die Tierknochenfunde aus dem latènezeitlichen Oppidum von Altenburg-Rheinau: Charakterisierung des Fundgutes Pferd, Hund, Hausgeflügel und Wildtiere/von Barbara Moser. Dissertation (München).

20. Leuzinger U, und Kultur Thurgau DFE (2000) *Die jungsteinzeitliche Seeufersiedlung Arbon-Bleiche 3: Befunde* (Departement für Erziehung und Kultur des Kantons Thurgau Frauenfeld).

21. Marti-Grädel E, Deschler-Erb S (2004) Viehhaltung und Jagd : Ergebnisse der Untersuchung der handaufgelesenen Tierknochen. *Die Jungsteinzeitliche Seeufersiedlung Arbon, Bleiche 3 : Umwelt Und Wirtschaft*, Archäologie im Thurgau. (Kantonsarchäologie Thurgau, Frauenfeld), pp 158–252.

22. Özdoğan M (2007) Von Zentralanatolien nach Europa Die Ausbreitung der neolithischen Lebensweise. *Vor 12000 Jahren in Anatolien - Die ältesten Monumente Der Menschheit*, ed Lichter C (Badisches Landesmuseum, Karlsruhe), pp 150–160.

23. Benecke N (2001) Die Tierwelt Thrakiens im Mittelholozän (ca. 6000--2000 v. Chr.)—anthropogene und natürliche Komponenten. *Beiträge zur Archäozoologie und prähistorischen Anthropologie* 3:29–38.

24. Buitenhuis H (1997) Asikli Höyük: A "'protodomestication'"site. *Proceedings of the 7 '" ICAZ Conference (Konstanz, 26/09/94—01/10/94). Paris: Antrhopozoologica*, pp 25–26.

25. Özbaşaran M (2012) Aşıklı. *The Neolithic in Turkey. New Excavations and New Research, Vol. 3: Central Turkey*, eds Özdoğan M, Başgelen N, Kuniholm P (Archaeology and Art Publications, Istanbul), pp 135–158.

26. Stiner MC, et al. (2014) A forager–herder trade-off, from broad-spectrum hunting to sheep management at Aşıklı Höyük, Turkey. *Proc Natl Acad Sci U S A* 111(23):8404–8409.

27. Fortin M (1990) Rapport préliminaire sur la seconde campagne de fouilles à Tell 'Atij et la première à Tell Gudeda (automne 1987), sur le Moyen Khabour. *syria* 67(2):219–256.

28. Zeder MA (2003) Food Provisioning in Urban Societies: A View from Northern Mesopotamia. *The Social Construction of Ancient Cities*, ed Smith M (Smithsonian Press), pp 156–183.

29. Rufolo SJ (2011) Specialized pastoralism and urban process in third millennium BC northern Mesopotamia: A treatment of zooarchaeological data from the Khabur Basin, Syria. Dissertation (Johns Hopkins University).

30. Schibler J, Furger AR, Schmid E, Reich J, Kaufmann B (1988) *Die Tierknochenfunde aus Augusta Raurica:(Grabungen 1955-1974)* (Römermuseum Augst).

31. Furger A, Deschler-Erb S (1992) *Das Fundmaterial aus der Schichtenfolge beim Augster*

Theater : typologische und osteologische Untersuchungen zur Grabung Theater-Nordwestecke 1986/87 (Römermuseum Augst, Augst).

32. Deschler-Erb S, Plogmann HH (2012) Ein Kultmahl im privaten Kreis-zu den Tierknochen aus dem Vorratskeller der Publikumsgrabung (2008-2010.058) von Augusta Raurica (um/nach Mitte 3. Jahrhundert n. Chr.). *Jahresberichte aus Augst und Kaiseraugst* 33:237–268.

33. De Cupere B, Duru R, Umurtak G (2008) Animal husbandry at the Early Neolithic to Early Bronze Age site of Bademağacı (Antalya province, SW Turkey): evidence from the faunal remains. *Travaux de la Maison de l'Orient et de la Méditerranée* 49(1):367–405.

34. Gorbunov VS (1986) Abashev culture of Southern Ural region. *Ufa*, p 95.

35. Knipper C, et al. (2018) A knot in a network: Residential mobility at the Late Iron Age proto-urban centre of Basel-Gasfabrik (Switzerland) revealed by isotope analyses. *Journal of Archaeological Science: Reports* 17:735–753.

36. Tresset A (1996) Le rôle des relations homme/animal dans l'évolution économique et culturelle des sociétés des Ve-IVe millénaires en Bassin Parisien: approche ethno-zootechnique fondée sur les ossements animaux. Dissertation (Paris 1).

37. Bandi H-G (1963) *Birsmatten-Basisgrotte: eine mittelsteinzeitliche Fundstelle im unteren Birstal* (Stämpfli).

38. Bandi HG, Others (1963) Birsmatten-Basisgrotte. *Ein Mittelsteinzeitliche Fundstelle im unteren Birstal (Acta Bernensia I) Bern: Verlag Stämpfli and Cie Bern.*

39. Bollongino R, et al. (2013) 2000 years of parallel societies in Stone Age Central Europe. *Science* 342(6157):479–481.

40. Lipson M, et al. (2017) Parallel palaeogenomic transects reveal complex genetic history of early European farmers. *Nature* 551:368.

41. Orschiedt J, Gehlen B, SCHöN W, GRöNING F (2012) The Neolithic and Mesolithic cave site Blätterhöhle at Hagen, Germany. *Notae Paehistoricae* 32:73–88.

42. Buko A, et al. (2013) A unique medieval cemetery from the 10th/11th century with chamber-like graves from Bodzia (central Poland): Preliminary result of the multidisciplinary research. *Archäologisches Korrespondenzblatt* 43(3):423–442.

43. Sobkowiak-Tabaka I, Kabaciński J (2012) Rescue excavation research conducted by the Team for Rescue Archaeological Research of the Centre for Prehistoric and Medieval Research of the Institute for Archaeology and Ethnology of the Polish Academy of Sciences (PAN) in motorway construction routes in years 2007-2008. *Raport 2007-2008* (Warszawa), pp 11–41.

44. Simonin D (1998) Boulancourt « le Châtelet », habitat fortifié du Néolithique moyen et du Bronze final. Opération de fouille programmée. *Rapport de synthèse, musée de Préhistoire d'Île-de-France, Nemours*:55.

45. Bãlãşescu A, Simonin D, Vigne J-D (2008) La faune du Bronze final IIIb du site fortifié de Boulancourt «le Châtelet» (Seine-et-Marne). *Bulletin de la Société préhistorique française* 105(2):371–406.

46. Czerniak L (2002) Settlements of the Brześć Kujawski type on the Polish Lowlands.

Archeologické rozhledy 54:9–22.

47. Nielsen PO (2004) Causewayed camps, palisade enclosures and central settlements of the Middle Neolithic in Denmark. *Journal of Nordic Archaeological Science* 14:19–33.

48. Rowley-Conwy P, Dobney K (2007) Wild boar and domestic pigs in Mesolithic and Neolithic southern Scandinavia. *Pigs and Humans: 10,000 Years of Interaction*, eds Albarella U, Dobney K, Ervynck A (Oxford University Press, Oxford), pp 131–155.

49. Zeiler JT (2003) Faunaresten uit een nederzettingscomplex te Odijk, gemeente Bunnik (Midden-IJzertijd, Laat-Romeinse tijd en Vroege Middeleeuwen). *Leeuwarden: Archaeobone*:16.

50. Detry C, Cardoso JL (2010) On some remains of dog (Canis familiaris) from the Mesolithic shell-middens of Muge, Portugal. *J Archaeol Sci* 37(11):2762–2774.

51. Detry C (2007) Paleoecologia e paleoeconomia do baixo Tejo no Mesolítico Final: o contributo do estudo dos mamíferos de Muge. Universidade de Salamanca/Universidade Autónola de Lisboa. *Salamanca/Lisboa (unpublished PhD thesis)*.

52. Steadman SR, et al. (2015) The 2013 and 2014 Excavation Seasons at Çadir Höyük on the Anatolian North Central Plateau. *Anatolica* 41:87–123.

53. Steadman SR, Ross JC, McMahon G, Gorny RL (2008) Excavations on the north-central plateau: The Chalcolithic and Early Bronze Age occupation at Çadır Höyük. *Anatolian Studies* 58:47–86.

54. Arbuckle BS (2009) Chalcolithic caprines, Dark Age dairy, and Byzantine beef: a first look at animal exploitation at Middle and Late Holocene Çadır Höyük, North Central Turkey. *Anatolica* 35:179–224.

55. Schoop UD (2011) Çamlıbel Tarlası, ein metallverarbeitender Fundplatz des vierten Jahrtausends v. Chr. im nördlichen Zentralanatolien. *Anatolian Metal V Bochum: Deutsches Bergbaumuseum*:53–68.

56. Bartosiewicz L, et al. (2013) Chalcolithic pig remains from Çamlibel Tarlasi, Central Anatolia. *Archaeozoology of the Near East X: Proceedings of the Tenth International Symposium on the Archaeozoology of South-Western Asia and Adjacent Areas*, eds Cupere B, Linseele V, Hamilton-Dyer S (Editions Peeters), pp 101–120.

57. Motzoi-Chicideanu I (2016) *Cimitirul din epoca bronzului de la Cârlomăneşti (Jud. Buzău)* (Editura Academiei Române).

58. Dumitraşcu V, Bălăşescu A (2004) Fauna din complexele arheologice de la Cârlomăneşti-Armanu (jud. Buzău). *Mousaios* (9):51–56.

59. Ashley S, et al. (2007) The resources of an upland community in the fourth millennium BC. *Uplands of Ancient Sicily and Calabria. The Archaeology of Landscape Revisited.*, ed Fitzjohn M, pp 59–80.

60. Dumitrescu V (1986) Stratigrafia aşezării-tell de pe Ostrovelul de la Căscioarele. *Cultură şi Civilizaţie la Dunărea de Jos* 2:73–82.

61. Bălăşescu A, Radu V, Moise D (2005) *Omul si mediul animal intre mileniile VII-IV i.e.n. la Dunarea de Jos* (Editura Cetatea de Scaun, București).

62. Krauss R (2014) *Ovčarovo-Gorata: eine frühneolithische Siedlung in Nordostbulgarien*

(Habelt-Verlag).

63. Benecke N, Ninov L (2002) Zur Nahrungswirtschaft der neolithischen Bevölkerungen im Gebiet des heutigen Bulgariens nach archäozoologischen Befunden. *Eiträge Zu Jungsteinzeitlichen Forschungen in Bulgarien. Saarbrücker Beiträge Zur Altertumskunde*, eds Lichardus-Itten M, Lichardus J, Nikolov V (Habelt, Bonn), pp 555–573.

64. Facorellis Y (2011) Sequential radiocarbon dating and calculation of the marine reservoir effect. *The Cave of the Cyclops: Mesolithic and Neolithic Networks in the Northern Aegean, Greece Volume II: Bone Tool Industries, Dietary Resources and the Paleoenvironment, and Archeometrical Studies*:361–372.

65. Trantalidou K (2011) From Mesolithic Fishermen and Bird Hunters to Neolithic Goat Herders: The Transformation of an Island Economy in the Aegean. *The Cave of the Cyclops: Mesolithic and Neolithic Networks in the Northern Aegean, Greece, Volume II: Bone Tool Industries, Dietary Resources and the Paleoenvironment, and Archeometrical Studies*, ed Sampson A (Cambridge University Press, Cambridge), pp 53–150.

66. Trantalidou K (2014) Trends in faunal taxonomic representation during the 9th millennium in the Aegean and the ambiguous status of suids. *La Transition En Méditerranée. Comment Des Chasseurs Devinrent Agriculteurs. International Conference*, eds Manen C, Perrin T, Guilaine J (Muséum d'Histoire Naturelle de Toulouse), pp 141–163.

67. Moundrea-Agraphioti A (2011) The Mesolithic and Neolithic Bone Implements. *The Cave of Cyclops. Mesolithic and Neolithic Networks in the Northern Aegean, Greece*, ed Sampson A (INSTAP Academic Press, Philadelphia), pp 3–49.

68. Mylona D (2011) Fish vertebrae. *The Cave of Cyclops. Mesolithic and Neolithic Networks in the Northern Aegean, Greece*, ed Sampson A (INSTAP Academic Press, Philadelphia), pp 237–266.

69. Powell J (2011) Non-Vertebral Fish Bones. *The Cave of Cyclops. Mesolithic and Neolithic Networks in the Northern Aegean, Greece*, ed Sampson A (INSTAP Academic Press, Philadelphia), pp 151–235.

70. Bıçakçı E (1998) An essay on the chronology of the PrePottery Neolithic settlements of the Taurus Region (Turkey) with the building remains and 14C dates. *Light on Top of the Black Hill*, eds Arsebük G, Mellink MJ, Schirmer W (Ege Yayınları, Istanbul), pp 137–150.

71. Erim-Özdoğan A (2012) Çayönü. *The Neolithic in Turkey: New Excavations & New Research*, eds Özdoğan M, Başgelen N, Kuniholm P (Archaeology & Art Publications, Istanbul), pp 185–269.

72. Özdoğan M, Özdoğan A (1990) Çayönü: a conspectus of recent work. *Préhistoire de Levant II*, eds Aurenche O, Cauvin MC (Éditions du CNRS, Paris), pp 387–396.

73. Primas M (1985) *Cazis-Petrushügel in Graubünden* (Juris, Zürich).

74. Bougard F (1997) Fouilles de l'École française de Rome à Leopolis-Cencelle (information). *Comptes rendus des séances de l'Académie des Inscriptions et Belles-Lettres* 141(2):479–490.

75. Minniti C (2016) Meat consumption as identity of status and profession during the

Middle Ages: a review of the zooarchaeological evidence from Rome and Latium (Italy). *Objects, Environment, and Everyday Life in Medieval Europe*, eds Jervis B, Broderick LG, Grau Sologestoa I (Brepols Publisher, Turnhout), pp 137–156.

76. Esmonde Cleary AS (2013) *Chedworth: Life in a Roman Villa* (The History Press).

77. Czebreszuk J, Szmyt M (2011) Kurhan, rów i palisada w tradycji kultury ceramiki sznurowej. Próba rekonstrukcji scenariuszy rytualnych. *Kurhany I Obrządek Pogrzebowy W IV-II Tysiącleciu P.n.e.*, eds Kowalewska-Marszałek H, Włodarczak P (Instytut Archeologii i Etnologii Polskiej Akademii Nauk Instytut Archeologii Uniwersytetu Warszawskiego, Warsaw), pp 123–131.

78. Bonsall C, et al. (2012) Interrelationship of age and diet in Romania's oldest human burial. *Naturwissenschaften* 99(4):321–325.

79. Bonsall C, et al. (2016) The "Clisurean"finds from Climente II cave, Iron Gates, Romania. *Quat Int* 423:303–314.

80. Bonsall C (2008) The Mesolithic of the iron gates. *Mesolithic Europe*:238–279.

81. Bonsall C, et al. (2015) New AMS 14C Dates for Human Remains from Stone Age Sites in the Iron Gates Reach of the Danube, Southeast Europe. *Radiocarbon* 57(1):33–46.

82. Bond JM, O'Connor TP (1999) Bones from medieval deposits at 16-22 Coppergate and other sites in York. *The Archaeology of York*, 15. (Council for British Archaeology, York).

83. Domanska L (1991) Obozowisko kultury janislawickiej w Dębach, woj. *Wloclawskie* stanowisko(Inowroclaw):29.

84. Wąs M (2008) O dystrybucji „czekolady" w kulturze janisławickiej z perspektywy technologii krzemieniarstwa. *Krzemień Czekoladowy W Pradziejach", Studia Nad Gospodarką Surowcami Krzemiennymi W Pradziejach*, eds Borkowski W, Libera. J, Sałacińska B, Sałaciński S (Warszawa), pp 171–184.

85. Arbuckle BS, Erek CM (2012) Late Epipaleolithic hunters of the central Taurus: Faunal remains from Direkli Cave, Kahramanmaraş, Turkey. *Int J Osteoarchaeol* 22(6):694–707.

86. Erek CM (2010) A new Epi-paleolithic site in the Northeast Mediterranean region: Direkli Cave (Kahramanmaras, Turkey). *Adalya* 13:1–17.

87. Lichardus I, et al. (2003) Bericht über die bulgarisch-deutschen Ausgrabungen in Drama (1996-2002). *Bericht der Römisch-Germanischen Kommission* 84:157–221.

88. Damian P, Bâltâc A (2007) *The Civil Roman Settlement at Ostrov-Durostorum* (Muzeul Brăilei).

89. Bălăşescu A, Radu V (2013) Studiul faunei din epoca romană de la Ostrov, punctul Ferma 4, județul Constanța. *Cercetări Arheologice* 20:231–250.

90. Wainwright GJ, Longworth IH (1971) Durrington Walls, Excavations 1966--1968 (--Reports of the Research Committee of the Society of Antiquaries of London 29). London. *Society of Antiquaries*.

91. Pearson MP, Stonehenge Riverside Project (England) (2012) *Stonehenge: Exploring the Greatest Stone Age Mystery* (Simon and Schuster).

92. Craig OE, et al. (2015) Feeding Stonehenge: cuisine and consumption at the Late Neolithic site of Durrington Walls. *Antiquity* 89(347):1096–1109.

93. Viner S, Evans J, Albarella U, Parker Pearson M (2010) Cattle mobility in prehistoric Britain: strontium isotope analysis of cattle teeth from Durrington Walls (Wiltshire, Britain). *J Archaeol Sci* 37(11):2812–2820.

94. Wright E, Viner-Daniels S, Parker Pearson M, Albarella U (2014) Age and season of pig slaughter at Late Neolithic Durrington Walls (Wiltshire, UK) as detected through a new system for recording tooth wear. *J Archaeol Sci* 52:497–514.

95. Albarella U, Serjeantson D (2002) A passion for pork: meat consumption at the British Late Neolithic site of Durrington Walls. *Consuming passions and patterns of consumption*:33–49.

96. Breu W (1986) Tierknochenfunde aus einer germanischen Siedlung bei Eggolsheim in Oberfranken:(2.-5. Jh. n. Chr.). Dissertation (Verlag nicht ermittelbar).

97. Kaufmann D B14 Eilsleben. *Archäologie in Der Deutschen Demokratischen Republik. Denkmale Und Funde. Band 2 Fundorte Und Funde.*, Fundorte und Funde., ed Hermann J (Urania-Verlag, Leipzig, Jena, Berlin), pp 410–411.

98. Arsuaga JL, et al. (2014) Neandertal roots: Cranial and chronological evidence from Sima de los Huesos. *Science* 344(6190):1358–1363.

99. Carretero JM, et al. (2008) a late pleistocene-early Holocene archaeological sequence of portalón de Cueva mayor (Sierra de atapuerca, burgos, Spain). *Munibe* 59:67–80.

100. Galindo-Pellicena MA, Carretero JM, Arsuaga JL (2014) Primary or Secondary Products?: the Nature of Capra and Ovis Exploitation within the Chalcolithic and Bronze Age Levels at Portalón (Atapuerca Hill, Burgos, Spain). Greenfield H. Animal Secondary Products.

101. Alday A, et al. (2017) Pottery with ramiform-anthropomorphic decoration from El Portalón de Cueva Mayor site (Sierra de Atapuerca, Burgos) and the globalized symbolic world of the first Neolithic. *Quat Int*. doi:10.1016/j.quaint.2017.10.044.

102. Pérez-Romero A, et al. (2017) An unusual Pre-bell beaker copper age cave burial context from El Portalón de Cueva Mayor site (Sierra de Atapuerca, Burgos). *Quat Int* 433:142–155.

103. Sverrisdóttir OÓ, et al. (2014) Direct estimates of natural selection in Iberia indicate calcium absorption was not the only driver of lactase persistence in Europe. *Mol Biol Evol* 31(4):975–983.

104. Günther T, et al. (2015) Ancient genomes link early farmers from Atapuerca in Spain to modern-day Basques. *Proc Natl Acad Sci U S A* 112(38):11917–11922.

105. Valdiosera C, et al. (2018) Four millennia of Iberian biomolecular prehistory illustrate the impact of prehistoric migrations at the far end of Eurasia. *Proc Natl Acad Sci U S A* 115(13):3428–3433.

106. Anderung C, et al. (2005) Prehistoric contacts over the Straits of Gibraltar indicated by genetic analysis of Iberian Bronze Age cattle. *Proc Natl Acad Sci U S A* 102(24):8431–8435.

107. Lira J, et al. (2010) Ancient DNA reveals traces of Iberian Neolithic and Bronze Age

lineages in modern Iberian horses. *Mol Ecol* 19(1):64–78.

108. Bordaz J, Bordaz L (1976) Erbaba excavations, 1974. *Türk Arkeoloji Dergisi* 23(2):39–44.

109. Bordaz, Alper-Bordaz (1979) The prehistory of the Beyshehir-Suglu Basin Turkey: Excavations at Erbaba. *National Geographic Research Reports*:157–165.

110. Bordaz J, Bordaz LA (1982) Erbaba: the 1977 and 1978 seasons in perspective. *Türk Arkeoloji Dergisi* 26(1):85–93.

111. Bordaz J (1973) Current Research in the Neolithic of South Central Turkey: Suberde, Erbaba and Their Chronological Implications. *Am J Archaeol* 77(3):282–288.

112. Perkins D (1973) The Beginnings of Animal Domestication in the near East. *Am J Archaeol* 77(3):279–282.

113. Makarewicz CA (1999) The faunal remains of Neolithic Erbaba: processes of domestication and the herding economy. Dissertation (Brandeis University).

114. Arbuckle BS (2013) The late adoption of cattle and pig husbandry in Neolithic Central Turkey. *J Archaeol Sci* 40(4):1805–1815.

115. Arbuckle BS, et al. (2014) Data sharing reveals complexity in the westward spread of domestic animals across Neolithic Turkey. *PLoS One* 9(6):e99845.

116. Saguì L (2001) L'esedra della Crypta Balbi tra tardo antico e alto medioevo. *Roma dall'antichità al medioevo: archeologia e storia nel Museo Nazionale Romano Crypta Balbi*:593–595.

117. Saguì L (2013) L'esedra della Crypta Balbi: archeologia, storia, musealizzazione. *Forma Urbis* 18(2):14–21.

118. De Grossi Mazzorin J, Minniti C (2001) L'allevamento e l'approvvigionamento alimentare di una comunità urbana. L'utilizzazione degli animali a Roma tra il VII e il X secolo. *Roma Dall'antichità Al Medioevo*, eds Arena MS, et al. (Archeologia e Storia nel Museo Nazionale Romano, Rome), pp 69–78.

119. Eingartner J, Eschbaumer P, Weber G, Engelien-Schmidt I (1993) *Der römische Tempelbezirk in Faimingen-phoebiana* (Zabern).

120. Andersen SH (2000) "Køkkenmøddinger"(shell middens) in Denmark: a survey. *Proceedings of the Prehistoric Society* (Cambridge University Press), pp 361–384.

121. Luzi C, Courtin J (2001) La céramique des niveaux préchasséens de la baume Fontbrégoua (Salernes, Var). *Bulletin de la Société préhistorique française* 98(3):471–484.

122. Rowley-Conwy P, Gourichon L, Helmer D, Vigne J-D (2013) Early Domestic Animals in Italy, Istria, the Tyrrhenian Islands and Southern France. *The Origins and Spread of Domestic Animals in Southwest Asia and Europe*, eds Colledge S, Conolly J, Dobney K, Manning K, Shennan S (Left Coast Press Editions), pp 161–194.

123. Jacobsen TW, Farrand WR, Farrand WR, Cooper FA, Vitaliano CJ (1987) *Franchthi Cave and Paralia: Maps, Plans, and Sections* (Indiana University Press).

124. Gramsch B (1992) Friesack mesolithic wetlands. *The Wetland Revolution in*

Prehistory. Conference, pp 65–72.

125. Sarti L, Balducci C, Brilli P, Corridi C (2002) Frilli-area C: un sito della media età del Bronzo a Sesto Fiorentino. *Rivista di scienze preistoriche* (52):261–292.

126. Smith PEL (1974) Ganj Dareh Tepe. *Paléorient* 2(1):207–209.

127. Zeder MA, Hesse B (2000) The initial domestication of goats (Capra hircus) in the Zagros mountains 10,000 years ago. *Science* 287(5461):2254–2257.

128. BOLEnDER DJ, Steinberg JM, Damiata BN (2011) Farmstead relocation at the end of the Viking Age: results of the Skagafjörður archaeological settlement survey. *Archaeologia Islandica* 9:77–99.

129. Peters J, Schmidt K (2004) Animals in the symbolic world of Pre-Pottery Neolithic Göbekli Tepe, south-eastern Turkey: a preliminary assessment. *Anthropozoologica* 39(1):179–218.

130. Schmidt K (2006) *Sie bauten die ersten Tempel: das rätselhafte Heiligtum der Steinzeitjäger: die archäologische Entdeckung am Göbekli Tepe* (Beck).

131. Ervynck A, Dobney K, Hongo H, Meadow R (2001) Born Free? New Evidence for the Status of“ Sus scrofa” at Neolithic Çayönü Tepesi (Southeastern Anatolia, Turkey). *Paléorient*:47–73.

132. Peters J, Arbuckle BS, Pöllath N (2014) Subsistence and beyond: animals in Neolithic Anatolia. *Neolithic in Turkey Istanbul: Arkeoloji ve Sanat Yayınları.*

133. Mahfroozi A (2007) The archaeology of eastern Mazandaran relying on excavations at Gohar Tepe. *Archaeological Reports* 7(2):347–368.

134. Soltysiak A, Mahfroozi A (2008) Short Fieldwork Report: Gohar Tepe and Goldar Tepe (Iran), seasons 2006–2007. *Bioarchaeology of the Near East* 2:71–77.

135. Brukner B (1988) *Die Siedlung der Vinča-Gruppe auf Gomolava (die Wohnschicht des Spätneolithikums und Frühäneolithikums-Gomolava Ia, Gomolava Ia-b und Gomolava Ib) und der Wohnhorizont des Äneolithischen Humus (Gomolava II).*

136. Boric D (2009) Absolute dating of metallurgical innovations in the Vinča culture of the Balkans. *Metals and Societies : Studies in Honour of Barbara S. Ottaway*, eds Kienlin TL, Roberts BW (Habelt, Bonn).

137. Orton D (2012) Herding, Settlement, and Chronology in the Balkan Neolithic. *European Journal of Archaeology* 15(1):5–40.

138. Clason AT (1979) The Farmers of Gomolava in the Vinca and La Tene Period. *Palaeohistoria* 21:42–81.

139. Orton DC (2008) Beyond hunting and herding: humans, animals, and the political economy of the Vinča period. Dissertation (University of Cambridge).

140. Voigt MM (1994) Excavations at Gordion 1988-89: The Yassihoyuk Stratigraphic Sequence. *Anatolian Iron Ages 3: The Proceedings of the Third Anatolian Iron Ages Colloquium Held at Van, 6-12 August 1990= Anadolu Demir Caglari 3= III. Anadolu Demir Caglari Sempozyumu Bildirileri, Van, 6-12 Agustos 1990* (The British Institute of Archaeology at Ankara), pp 265–293.

141. Miller NF, Zeder MA, Arter SR (2009) From Food and Fuel to Farms and Flocks: The Integration of Plant and Animal Remains in the Study of the Agropastoral Economy at Gordion, Turkey. *Curr Anthropol* 50(6):915–924.

142. König E (1993) Tierknochenfunde aus einer Feuchtbodensiedlung der Chamer Gruppe in Griesstetten, Ldkr. Neumarkt. Dissertation (LMU-München).

143. Kaiser T, Forenbaher S (2016) Navigating the Neolithic Adriatic. *Fresh Fields and Pastures New*, eds Lillios KT, Chazan M (Sidestone Press, Leiden), pp 145–164.

144. Petrić N (2004) Kultura Gudnja i primjeri importa u neolitiku Dalmacije. *Prilozi Instituta za arheologiju u Zagrebu* 21(1):197–207.

145. Minichreiter K (2005) The first farmers of northern Croatia. *A Short Walk through the Balkans: The First Farmers of the Carpathian Basin and Adjacent Regions* (Institute of Archaeology, University College London, London).

146. Mortensen P (1975) Survey and soundings in the Holailan Valley 1974. *Proceedings of the Third Annual Symposium on Archaeological Research in Iran*, pp 1–2.

147. Zeder M (2008) Animal domestication in the Zagros: an update and directions for future research. *Travaux de la Maison de l'Orient et de la Méditerranée* 49(1):243–277.

148. Schmidt K (1995) Investigations in the Upper Mesopotamian Early Neolithic: Göbekli Tepe and Gürcütepe. *Neo-Lithics* 2(95):9–10.

149. Schmidt K (2000) Göbekli Tepe, Southeastern Turkey: A Preliminary Report on the 1995-1999 Excavations. *Paléorient* 26(1):45–54.

150. Mashkour M (2006) Boars and pigs: A view from the Iranian plateau. *De La Domestication Au Tabou: Le Cas Des Suidés Dans Le Proche-Orient Ancient. Travaux de La Maison d'Archéologie et d'Ethnologie de Nanterre 1*, eds Lion B, Michel C (De Boccard), pp 155–163.

151. Mohaseb Karimlu F (2012) Exploitation des animaux de l'Âge du Bronze au début de la période Islamique dans le Nord-ouest de l'Iran: l'étude archéozoologique de Haftavan Tepe. Dissertation (Paris 1).

152. Mohaseb AF, Mashkour M (2017) Animal Exploitation from the Bronze Age to the Early Islamic Period in Haftavan Tepe (Western Azerbaijan--Iran). *Archaeozoology of the Near East 9 Proceedings of the 2008 Al Ain-Abu Dhabi Conference*, eds Mashkour M, Beech MJ (Oxbow Books Limited), pp 146–170.

153. Rosenberg M, Redding RW (2002) Hallan Çemi and Early Village Organization in Eastern Anatolia. *Life in Neolithic Farming Communities: Social Organization, Identity, and Differentiation*, ed Kuijt I (Springer US, Boston, MA), pp 39–62.

154. Redding RW (2005) Breaking the mold: A consideration of variation in the evolution of animal domestication. *First Steps of Animal Domestication New Archaeozoological Approaches*, eds Vigne J-D, Peters J, Helmer D (Oxbow Books, Oxford), pp 41–48.

155. Popovici D, Haşotti P, Galbenu D, Nicolae C (1992) Cercetările arheologice din tell-ul de la Hârşova (1988). *Cercetări Arheologice* 9:8–18.

156. Popovici D, et al. (2000) Les recherches archéologiques du tell de Hârşova (dép. de Constanţa) 1997–1998. *Cercetari Arheologice* 11(1):13–123.

157. Balasescu A, Radu V, Moise D (2005) *Omul si mediul animal intre mileniile VII-IV ien la Dunarea de Jos*.

158. Kussinger S (1988) Tierknochenfunde von Lidar Höyük in Südostanatolien (Grabungen 1979-86).-229 S. Dissertation (Dissertation Universität München).

159. Stahl U (1989) Tierknochenfunde vom Hassek Höyük (Südostanatolien). Dissertation (Ludwig-Maximilians-Universität).

160. Boessneck J (1992) Besprechung der Tierknochen-und Molluskenreste von Hassek Höyük. *Hassek Höyük: naturwissenschaftliche Untersuchungen und lithische Industrie*:58–74.

161. Ottoni C, et al. (2013) Pig domestication and human-mediated dispersal in western Eurasia revealed through ancient DNA and geometric morphometrics. *Mol Biol Evol* 30(4):824–832.

162. Fewkes VJ (1932) Excavations in the Late Neolithic Fortress of Homolka in Bohemia A Preliminary Report. *Proc Am Philos Soc* 71(6):357–392.

163. Ehrich RW, Pleslová-Štiková E (1968) *Homolka: an eneolithic site in Bohemia* (Peabody Museum, Oxford).

164. Bogucki PI (1979) Mammal Remains from HutB at the Eneolithic Settlement of Homolka (Bohemia). *Archeologické Rozhledy Praha* 31(1):83–92.

165. van der Velde HM (2001) Houten, Hoogdijk terrein 89. *Onderzoek in Het Kader van de Vinexlocatie Loerik, Hofstad II* (Archeologisch Diensten Centrum, Bunschoten).

166. Grönvold K, et al. (1995) Ash layers from Iceland in the Greenland GRIP ice core correlated with oceanic and land sediments. *Earth Planet Sci Lett* 135(1):149–155.

167. Sigurgeirsson MÁ, Hauptfleisch U, Newton A, Einarsson Á (2013) Dating of the Viking Age Landnám Tephra Sequence in Lake Mývatn Sediment, North Iceland. *Journal of the North Atlantic* (21):1–11.

168. Ascough PL, et al. (2007) Reservoirs and radiocarbon: 14C dating problems in Mývatnssveit, Northern Iceland. *Radiocarbon* 49:947–961.

169. Boroneanț A, Bonsall C (2016) The Icoana burials in context. *Mesolithic burials--rites, symbols and social organisation of early Postglacial communities* 2:757–780.

170. Girdland-Flink L (2013) Investigating patterns of animal domestication using ancient DNA. Doctoral (Durham University).

171. Braidwood LS (1983) *Prehistoric archeology along the Zagros Flanks* (Oriental Inst Pubns Sales).

172. Flannery KV (1983) Early pig domestication in the Fertile Crescent: A retrospective look. *The Hilly Flanks and Beyond: Essays on the Prehistory of Southwestern Asia Studies in Ancient Oriental Civilization* 36:163–188.

173. Price MD, Arbuckle BS (2015) Early Pig Management in the Zagros Flanks: Reanalysis of the Fauna from Neolithic Jarmo, Northern Iraq. *Int J Osteoarchaeol* 25(4):441–453.

174. Stampfli HR (1983) The fauna of Jarmo with notes on animal bones from Matarrah,

the Amuq, and Karim Shahir. *Prehistoric Archaeology along the Zagros Flanks Oriental Institute Publications* 105:431–483.

175. Vagedes K (2001) Die Tierknochenfunde aus Karlburg: ein Vergleich zwischen Burg und Talsiedlung. *Karlburg--Rosstal--Oberammerthal: Studien zum Frühmittelalterlichen Burgenbau in Nord Bayern, Frühgeschichtliche und ProvinzialRömische Archäologie Materialen und Forschungen* 5:305–315.

176. Manaseryan N (2013) Armenia: wild boar in all issues. *Studii de Preistorie* 10:245–248.

177. Vigne J-D, et al. (2012) First wave of cultivators spread to Cyprus at least 10,600 y ago. *Proceedings of the National Academy of Sciences* 109(22):8445–8449.

178. Vigne J-D, et al. (2017) Klimonas, a late PPNA hunter-cultivator village in Cyprus: new results. *Nouvelles Données Sur Les Débuts Du Néolithique à Chypre / New Data on the Beginnings of the Neolithic in Cyprus*, séances en ligne., eds Vigne J-D, Briois F, Tengberg M (Société Préhistorique Française, Paris), pp 21–46.

179. Vigne J-D, et al. (2009) Pre-Neolithic wild boar management and introduction to Cyprus more than 11,400 years ago. *Proc Natl Acad Sci U S A* 106(38):16135–16138.

180. Zazzo A, Lebon M, Quiles A, Reiche I, Vigne J-D (2015) Direct Dating and Physico-Chemical Analyses Cast Doubts on the Coexistence of Humans and Dwarf Hippos in Cyprus. *PLoS One* 10(8):e0134429.

181. Vigne J-D, Carrère I, Briois F, Guilaine J (2011) The Early Process of Mammal Domestication in the Near East: New Evidence from the Pre-Neolithic and Pre-Pottery Neolithic in Cyprus. *Curr Anthropol* 52(S4):S255–S271.

182. Seidel U (2008) *Michelsberger Erdwerke im Raum Heilbronn: Neckarsulm-Obereisesheim "Hetzenberg" und Ilsfeld "Ebene", Lkr. Heilbronn, Heilbronn-Klingenberg "Schloßberg", Stadtkreis Heilbronn. Text, Literatur und Anhänge* (K. Theiss, Stuttgart).

183. Seidel U, et al. (2016) Die Zeit der großen Gräben: Modelle zur Chronologie des Michelsberger Fundplatzes von Heilbronn-Klingenberg „Schlossberg", Stadtkreis Heilbronn, Baden-Württemberg. *Praehistorische Zeitschrift* 91(2). doi:10.1515/pz-2016-0022.

184. Maziar S (2015) Settlement dynamics of the Kura-Araxes culture: An overview of the Late Chalcolithic and Early Bronze Age in the Khoda Afarin Plain, North-Western Iran. *Paléorient* 41(1):25–36.

185. Füleky G, Márity E (1997) Environmental changes in Budaújlak (Pannonia Province, Hungary) in the Roman period. *Landscapes in Flux: Central and Eastern Europe in Antiquity*, Colloquia Pontica 3., eds Chapman J, Dolukhanov PM (Oxbow, Oxford), pp 231–239.

186. Márity E (1996) Kolosy tér 6.: római kor, középkor, őskor. *Régészeti Füzetek* 1(47):35.

187. Krauss R (2006) *Die prähistorische Besiedlung am Unterlauf der Jantra vor dem Hintergrund der Kulturgeschichte Nordbulgariens: Praistoriiata po dolnoto techenie na r. Iantra na fona na kulturnata istoriia na severna Bulgariia* (VML, Verlag Marie Leidorf).

188. Lichardus-Itten M, Demoule JP, Pernicheva L, Grębska-Kulova M, Kulov IK (2006) an Early Neolithic site in South-West Bulgaria and its importance for European Neolithization. *Aegean--Marmara--Black Sea: the Present State of Research on the Early Neolithic Langenweissbach* 94.

189. Honkisz B (2014) Gospodarcze i społeczne znaczenie nieudomowionych zwierząt dla społeczności wczesnoneolitycznych w wybranych rejonach Europie Środkowej. *Folia Praehistorica Posnaniensia* 19.

190. Kołodziej B (2011) Pochówki zwierzęce w neolicie na terenie ziem Polski. *Materiały i Sprawozdania* 32:55–106.

191. Ott-Luy S (1988) *Die Tierknochenfunde aus der mittelneolithischen Station von Künzing-Unternberg, Ldkr. Deggendorf.*

192. Boessneck J (1958) *Tierknocken aus spätneolithischen Siedlungen Bayerns* (Munchen).

193. Kennet D (1997) Kush: a Sasanian and Islamic-period archaeological tell in Ras al-Khaimah (UAE). *Arabian archaeology and epigraphy* 8(2):284–302.

194. L. B, F. D, Rousselet O, Manen C (2018) Early farming economy in Mediterranean France: fruit and seed remains from the Early to Late Neolithic levels of the site of Taï (ca 5300–3500 cal bc). *Vegetation History and Archaeobotany* 28(1):17–34.

195. Rova E, Weiss H (2002) The origins of North Mesopotamian Civilization, Ninevite 5 Chronology, Economy, Society (Subartu IX).

196. Ervynck A (1994) "De Burcht" te Londer-zeel. Bewonings-ge-schiedenis van een motte en een bakstenen kasteel. *Archeologie in Vlaanderen Monografie I*, ed Ervynck A (Instituut voor het Archeologisch Patrimonium, Zelik).

197. Ervynck A, Van Neer W, Van der Plaetsen P (1994) Dierlijke resten. *"De Burcht" Te Londer-zeel. Bewonings-ge-schiedenis van Een Motte En Een Bakstenen Kasteel (Archeologie in Vlaanderen Monografie I)*, ed Ervynck A (Instituut voor het Archeologisch Patrimonium, Zellik), pp 99–170.

198. Pyzel J (2012) Preliminary Results of Large Scale Emergency Excavations in Ludwinowo 7, Comm. Włocławek. *Siedlungsstruktur Und Kulturwandel in Der Bandkeramik: Beiträge Der Internationalen Tagung "Neue Fragen Zur Bandkeramik Oder Alles Beim Alten*:160–166.

199. Micu C, Maille M (2002) Recherches archéologiques dans le cadre de l'établissement-tell de Luncaviţa (dép. de Tulcea). *Studii de preistorie* 1:115–129.

200. Comşa E (1952) Săpături arheologice la Luncaviţa. *Materiale* 5:221–225.

201. Bălăşescu A (2003) L'étude de la faune des mammifères découverts a Luncaviţa. *Peuce* 1(14):453–468.

202. Commenge C (2009) Neolithic settlement patterns in the alluvial plains of Macedonia. *Ol'Man River. Geo-Archaeological Aspects of Rivers and River Plains*, Archaeological Reports Ghent University., eds De Dapper M, Vermeulen F, Deprez S, Taelman D (Ghent Academia Press, Ghent), pp 229–241.

203. Bălăşescu A (2014) Arheozoologia neo-eneoliticului de pe Valea Teleormanului. *Muzeul Naţional de Istorie a României, Colecţia Muzeului Naţional, Seria Cercetări*

Puridisciplinare (Editura Mega, Cluj Napoca), p 216.

204. Evin A, et al. (2015) Unravelling the complexity of domestication: a case study using morphometrics and ancient DNA analyses of archaeological pigs from Romania. *Philos Trans R Soc Lond B Biol Sci* 370(1660):20130616.

205. Çakırlar C (2013) Rethinking Neolithic subsistence at the gateway to Europe with new archaeozoological evidence from Istanbul. *Barely Surviving or More than Enough*, eds Groot M, Lentjes D, Zeiler J (Sidestone Press, Leiden), pp 59–79.

206. Thuesen I (1994) Mashnaqa. *Am J Archaeol* 98(1):111–112.

207. Hole FA (2007) Khabur Basin Project - 1986-2001. *Ann Archeol Syrie* (45-46):11–20.

208. Braidwood RJ, Braidwood L, Smith JG, Leslie C (1952) Matarrah: A Southern Variant of the Hassunan Assemblage, Excavated in 1948. *J Near East Stud* 11(1):1–75.

209. Mignot PH (1994) Le Château de Montaigle. *Fiche Patrimoine* 94:12.

210. Gautier A, Fiers E (2009) Restes animaux des occupations de l'âge de Fer, gallo-romaine et médiévale du site castral de Montaigle (Namur). *VA, VIE ARCHEOLOGIQUE* (68):85–100.

211. Gourichon L, Helmer D (2008) Étude archéozoologique de Mureybet. *Tell Mureybet, Un Site Néolithique Dans Le Moyen Euphrate Syrien*, ed Ibañez JJ (Archaeopress, Oxford), pp 115–227.

212. Willcox G, Fornite S, Herveux L (2008) Early Holocene cultivation before domestication in northern Syria. *Veg Hist Archaeobot* 17(3):313–325.

213. Gurina NN (1955) New Neolithic sites in eastern Estonia. *Drevnie Poseleniya I Gorodishcha* (Tallinn), pp 153–175.

214. Paaver K (1965) Theriofauna genesis and variability of mammals in Baltic Republic in Holocene. *Institut Zoologii I Botaniki* (Tartu), p 494 pp. + XII tabl.

215. Mol D, Post K, Reumer JWF, de Vos J, Laban C (2003) Het Gat: preliminary note on a Bavelian fauna from the North Sea with possibly two mammoth species. *Deinsea* 9(1):253–266.

216. Mol D, et al. (2004) The Eurogeul - first report of paleontological, palynological and archeological investigations of this part of the North Sea. *Quat Int* 142-143(2006):178–185.

217. Mol D, Reumer JWF (2010) The North Sea--Maasvlakte 2 project. *Quaternaire, Hors Serie, 3. Volume of Abstracts of the Vth International Conference on Mammoths and Their Relatives, Le Puyen-Velay, August 30--September 5.*

218. Kolfschoten T van, Laban C, Others (1995) Pleistocene terrestrial mammal faunas from the north Sea area. *Mededelingen Rijks Geologische Dienst* 52:135.

219. Reumer JWF, et al. (2003) Late Pleistocene survival of the saber-toothed cat Homotherium in northwestern Europe. *J Vert Paleontol* 23(1):260–262.

220. Mol D, et al. (2008) *Kleine encyclopedie van het leven in het Pleistoceen: mammoeten, neushoorns en andere dieren van de Noordzeebodem* (Veen Magazines,

Diemen).

221. Kortenbout van der Sluijs G (1971) Bones of mammals from the Brown Bank area (North Sea). *Mesolithic bone and antler implements from the North Sea and from the Netherlands Berichten van de Rijksdienst voor het Oudheidkundig Bodemonderzoek*:20–21.

222. Van Der Sluijs G. K (1971) Fossil bones from the North Sea.- in: Proceedings Nederlandse Dierkundige Vereniging (Netherlands Zoological Society). *Neth J Zool* 21(2):214.

223. Mordant D, Mordant C (1992) Noyen-sur-Seine: a Mesolithic waterside settlement. *The Wetland Revolution in Prehistory. Conference*, pp 55–64.

224. Mordant D, Valentin B, Vigne J-D (2013) Noyen-sur-Seine, twenty-five years on. *Mesolithic Palethnography Research on Open-Air Sites between Loire and Neckar (Proceedings from the International Round-Table Meeting, Paris, November 26 – 27, 2010)*, Séances de la Société préhistorique française, 2012., eds Valentin B, et al. (Société préhistorique française, Paris), pp 37–49.

225. Marinval-Vigne M-C, et al. (1993) Archéologie et paléo-environnement: Noyen-sur-Seine (Seine-et-Marne). *Mémoires du Groupement archéologique de Seine-et-Marne* (1):21–31.

226. Bridault A, et al. (2000) Wild boar - age at death estimates: the relevance of new modern data for archaeological skeletal material. 1. Presentation on the corpus. Dental and epiphyseal fusion ages. *Anthropozoologica* 31:11–38.

227. Hofmann R (2007) *Prospektionen und Ausgrabungen in Okoliste (Bosnien-Herzegowina): Siedlungsarchäologische Studien zum zentralbosnischen Spätneolithikum (5300-4500 v. Chr.)* (Bericht der Römisch-Germanischen Kommission).

228. Vanhoutte S (2015) The late Roman coastal fort of Oudenburg (Belgium): spatial and functional transformations within the fort walls. *Roman Military Architecture on the Frontiers. Armies and Their Architecture in Late Antiquity*, eds Collins R, Symonds M, Weber M (Oxbow Books, Oxford & Philadelphia), pp 62–75.

229. Ervynck A, et al. (2017) Het castellum aan het eind van de wereld. Vlees en vis voor de soldaten te Oudenburg. *Signa* 6:41–45.

230. Roudil J-L (1987) Le gisement Néolithique de la Baume d’Oullins le Garn-Gard. *Premières Communautés Paysannes En Méditerranée Occidentale*, eds Guilaine J, Courtin J, Roudil J-L, Vernet J-L (CNRS Éditions), pp 523–529.

231. Roudil JL, Van Willigen S (2002) La céramique du Néolithique ancien de la Baume d’Oullins (Le Garn, Gard). *Ardèche Archéologie* 19:1–26.

232. Helmer D, Gourichon L, Sidi Maamar H, Vigne J-D (2005) L’élevage des caprinés néolithiques dans le sud-est de la France: saisonnalité des abattages, relations entre grottes-bergeries et sites de plein air. *Anthropozoologica* 40(1):167–189.

233. Turnbull PF, Reed CA (1974) The Fauna from the Terminal Pleistocene of Palegawra Cave, A Zarzian Occupation Site in Northeastern Iraq. *Fieldiana* 63(3):81–146.

234. Radi G, Tozzi C, Arobba D, Battentier J (2017) Modelling the earliest north-western dispersal of Mediterranean Impressed Wares: new dates and Bayesian chronological model. *Documenta Praehistorica* 44:54–77.

235. Benecke N, et al. (2013) Pietrele in the Lower Danube region: integrating archaeological, faunal and environmental investigations. *Documenta Praehistorica* 40:175.

236. Garašanin MV (1951) *Hronologija vinčanske grupe* (Univerza, Arheološki seminar, Ljubljana).

237. Radivojević M, Kuzmanović-Cvetković J (2014) Copper minerals and archaeometallurgical materials from the Vinča culture sites of Belovode and Pločnik: overview of the evidence and new data. *Starinar* (64):7–30.

238. Stalio B (1960) Plocnik-Prokuplje-naselje. *Arheološki pregled* 2:33–36.

239. Palincaş N (1996) Valorificarea arheologică a probelor 14C din fortificaţia aparţinând Bronzului târziu de la Popeşti (jud. Giurgiu). *SCIVA* 47(3):239–288.

240. Dumitrascu V (2004) THE 2001 CAMPAIGN IN THE EARLY IRON AGE SETTLEMENT AT POPE: A ZOOARCHAEOLOGICAL ANALYSIS. *Dacia* 48:71–75.

241. Miracle P (1997) Early Holocene foragers in the karst of northern Istria. *Poročilo O Raziskovanju Paleolita, Neolita in Eneolita v Sloveniji*, XXIV., pp 43–61.

242. Czerniak L, et al. (2016) House time: Neolithic settlement development at Racot during the 5th millennium CAL BC in the Polish lowlands. *J Field Archaeol* 41(5):618–640.

243. Rieckhoff S (1987) *Archäologisches Museum im BMW-Werk Regensburg* (Bayer. Motoren-Werke Aktienges.).

244. Vagedes K, Peters J (2001) Die Faunenreste aus der karolingisch-ottonischen Reichsburg in Rosstal, Landkreis Fürth. *Studien Zum Frühmittelalterlichen Burgbau in Nordbayern*, eds Ettel P, Karlburg-Rosstal-Oberammertal. (Rahden/Westf., Leidorf), pp 317–339.

245. Lesur J, Gasco J, Tresset A, Vigne J-D (2001) Un approvisionnement chasséen caussenard exclusivement fondé sur la chasse? La faune de Roucadour (Lot). *Préhistoire du Sud-Ouest* (8):71–90.

246. De Cupere B (2001) *Animals at ancient Sagalassos evidence of the faunal remains* (Brepols, Turnhout).

247. Malez M (1964) Šandalja bei Pula-ein neuer und wichtiger paläolithischer fundort in Istrien. *Bulletin Scientifique du Conseil des Académies des Sciences et des Arts de la RSF Yougoslavie* 9(6):154–155.

248. Miracle PT (1995) Broad-spectrum adaptations re-examined: Hunter-gatherer responses to Late Glacial environmental changes in the eastern Adriatic. Dissertation (The University of Michigan).

249. Oros Sršen A, Brajković D, Radović S, Mauch Lenardić J, Miracle PT (2014) The Avifauna of Southern Istria (Croatia) During the Late Pleistocene: Implications for the Palaeoecology and Biodiversity of the Northern Adriatic Region. *Int J Osteoarchaeol* 24(3):289–299.

250. Freguglia M, Lo Vetro D, Volante N (2005) Santa Maria in Selva di Treia, Macerata: l'area 1. *Atti della XXXVIII Riunione scientifica*:856–860.

251. Dinu A, Soficaru A, Miritoiu D (2007) The Mesolithic at the Danube's Iron Gates: new radiocarbon dates and old stratigraphies. *Documenta Praehistorica* 34(31):e52.

252. Beregovaya NF (1960) Paleolithic sites of USSR. *Materialy i Issledovaniya po Archeologii SSSR* 81:1–218.

253. Benecke N (2006) Zur Datierung der Faunensequenz am Abri Šan-Koba (Krim, Ukraine). *Beiträge zur Archäozoologie und Prähistorischen Anthropologie* 12:15.

254. Bibikov SN, Stanko VN, Koen VY (1994) Final Paleolithic and Mesolithic of Crimean mountain. *Vest, Odessa*:238.

255. Kośko A, Kirkowski R (1997) Siniarzewo, st. 1, gm. Zakrzewo, woj. włocławskie, AZP 46-44/115. *Informator Archeologiczny: badania* 31.

256. Vogler U (1997) Faunenhistorische Untersuchungen am Sirkeli Höyük/Adana, Türkei. Diss. med. vet. (LMU-München).

257. Larina OV, Wechler KP, Dergačev VA, Kovalenko SI, Bikbaev VM (1997) New field surveys on the Mesolithic and Neolithic of Moldova. *Vestigii Arheologice din Moldova*:62–110.

258. Yanevich OO (1993) Shpan Mesolithic culture. *Archeologia* `:3–15.

259. Benecke N (2000) Mesolithic hunters of the Crimean mountains: The fauna from the rock shelter of Shpan'-Koba. *Archaeozoology of the Near East*, eds Mashkour M, Choyke AM, Buitenhuis H, & Poplin F (Groningen), pp 107–120.

260. Martini F, Poggiani Keller R, Sarti L Bell Beaker in everyday life. *Proceedings of the 10th Meeting Archéologie et Gobelets* (Museo Fiorentino di Preistoria , Firenzevia).

261. Sarti L (2006) Presenze di vasi a bocca quadrata in Italia centrale: problemi e prospettive. *Preistoria dell'Italia Settentrionale*, eds A. P, Visentini P (Mueo Friulano di Storia naturale, Udine), pp 193–210.

262. Volante N (2014) La cultura dei vasi a Bocca quadrata e il pieno Neolitico. *A Cura Di, Passaggi a Nord Ovest*, eds Poggesi G, Sarti L (Millenni 10, Firenze), pp 46–50.

263. Kuz'min SL (2008) Ladoga in early Middle Ages (middle VIII – beginning XII century). *Issledovanie Archeologicheskikh Pamyatnikov Epokhi Srednevekoviya*, ed Vinogradov AV (St. Petersburg), pp 69–94.

264. Bordaz J (1970) *The Suberde excavations, Southwestern Turkey: an interim report* (Turk Tarih Kurumu Basimevi).

265. Solecki RS (1964) An archaeological reconnaissance in the Beysehir-Sugla area of south western Turkey'. *Turk Arkeoloji Dergisi* 13(1):129–125.

266. Arbuckle BS (2008) Revisiting Neolithic Caprine Exploitation at Suberde, Turkey. *J Field Archaeol* 33(2):219–236.

267. Perkins D Jr, Daly P (1968) A hunters' village in neolithic Turkey. *Sci Am* 219(5):97–106.

268. King T (2017) Analysis of the suid (Sus scrofa) faunal assemblage from Neolithic Suberde Höyük, Southwest Turkey. Curriculum in Archaeology (UNC-CH).

269. McComish JM (2015) Archaeological excavations at 12-18 Swinegate, 14 Little Stonegate and 18 Back Swinegate.

270. Mazar A, Amitai-Preiss N (2006) *Excavations at Tel Beth-Shean 1989-1996* (Israel Exploration Society, Jerusalem).

271. Horwitz LK Mammalian remains from Areas H, L, P and Q. *Excavations at Tel Beth-Shean 1989-1996 Vol. I. From the Late Bronze Age IIB to the Medieval Period.*, ed Mazar A (Israel Exploration Society, Jerusalem), pp 689–710.

272. Maeir AM (2012) *Tell es-Safi/Gath I: Report on the 1996--2005 Seasons. Ägypten und Altes Testament 69* (MANFRED GÖRG, MÜNCHEN).

273. Meiri M, et al. (2013) Ancient DNA and population turnover in southern levantine pigs--signature of the sea peoples migration? *Sci Rep* 3:3035.

274. Gitin S (2012) Tel Miqne-Ekron. Summary of Fourteen Seasons of Excavation 1981–1996 and Bibliography 1982–2012. 2012. *The Tel Miqne-Ekron Excavation and Publications Project* (W.F. Albright Institute of Archaeological Research and Hebrew University, Jerusalem).

275. Dothan T, Garfinkel Y, Gitin S (2016) *Tel Miqne-Ekron Excavations, 1985–1988, 1990, 1992–1995: Field IV Lower—The Elite Zone, Part 9/3A: The Iron Age I and IIC Early and Late Philistine Cities Database* (Eisenbrauns, Winona Lake).

276. Hesse B (1986) Animal Use at Tel Miqne-Ekron in the Bronze Age and Iron Age. *Bull Am Schools Orient Res* (264):17–27.

277. Lev-Tov JSE (2000) Pigs, Philistines, and the Ancient Animal Economy of Ekron from the Late Bronze Age to the Iron Age II. Dissertation (University of Tennessee, Knoxville).

278. Maher EF (2004) Food for the Gods: The Identification of Philistine Rites of Animal Sacrifice. PhD (University of Illinois at Chicago).

279. Maher EF, and Hesse B (2016) The Middle Bronze Age II and Iron Age I faunal remains. Tel Miqne-Ekron Excavations 1985-1988, 1990, 1992-1995. Field IV Lower – The Elite Zone. Part 1. The Iron Age I Early Philistine City. Winona Lake. *Eisenbrauns*, eds Dothan T, Garfinkel Y, and Gitin S, pp 515–542.

280. Bartosiewicz L (2016) Halaf Period Animal Remains from Tell Aqab, Northeastern Syria. *Bones and Identity: Zooarchaeological Approaches to Reconstructing Social and Cultural Landscapes in Southwest Asia*, eds Marom N, Yeshurun R, Weissbrod L, and Bar-Oz G (Oxbow Books, Oxford & Philadelphia), pp 125–155.

281. Hilditch J, Kiriatzi E, Psaraki K, Aravantinos V (2008) Early Helladic II pottery from Thebes: An integrated typological, technological and provenance study. *BAR INTERNATIONAL SERIES* 1746:263.

282. Vika E (2011) Diachronic dietary reconstructions in ancient Thebes, Greece: results from stable isotope analyses. *J Archaeol Sci* 38(5):1157–1163.

283. Aravantinos V (1997) Θήβα. *ArchDeltio* 52:354–359.

284. Mashkour M, Bailon S (2010) Animal remains from Tol-e Bashi (Ramjerd plain-Fars), a Late Neolithic / Chalcolithic settlement in South-West Iran. Social Life in a Neolithic Village. *The 2003 Excavations at Tol-E-Baši, Iran*, eds Pollock S, Bernbeck R,

Abdi K (Archäologie Mitteilungen, Turan), pp 215–229.

285. Andersen NH (1982) A Neolithic Causewayed Camp at Trelleborg near Slagelse, West Zealand. *Journal of Danish Archaeology* 1(1):31–33.

286. Davidsen K (1978) *The final TRB culture in Denmark: a settlement study* (Akademisk Forlag, Copenhagen).

287. Miller R, Zwyns N, Otte M, Stevens C, Stewart J (2012) La séquence mésolithique et néolithique du Trou Al'Wesse (Belgique): résultats pluridisciplinaires. *Anthropologie* 116(2):99–126.

288. Grundbacher HR, Stampfli B (1977) Tierknochenfunde. Erster Bericht. *Die Neolithischen Ufersiedlungen von Twann* (Band, Bern).

289. Becker C (1981) Tierknochenfunde. Dritter Bericht. *Die Neolithischen Ufersiedlungen von Twann* (Band, Bern).

290. Becker C, Johansson F (1981) Tierknochenfunde. Zweiter Bericht. *Die Neolithischen Ufersiedlungen von Twann* (Band, Bern).

291. Stampfli HR (1980) Tierknochenfunde: Dokumentation. *Die Siedlungen Der Horgener Kultur. Die Neolithischen Ufersiedlungen von Twann*, ed Furger AR (Band, Bern), pp 141–160.

292. Schier W, Draşovean F (2004) Der spätneolithisch-frühkupferzeitliche Tell von Uivar, jud. Timiş, Rumänien. Vorbericht über die Prospektionen und Ausgrabungen 1998–2002. *Praehist Zeitschr* 79:145–230.

293. El Susi G (2017) Animal Bones from the Neolithic (Szakálhát) Levels at Uivar (Timiş County). *Georgeta El Susi* 31:29–54.

294. Çilingiroğlu Ç (2012) *The Neolithic pottery of Ulucak in Aegean Turkey: organization of production, interregional comparisons and relative chronology* (BAR International Series, Oxford).

295. Çilingiroğlu Ç, Çakırlar C (2013) Towards configuring the neolithisation of Aegean Turkey. *Documenta Praehistorica* 40:21–29.

296. Çakırlar C (2012) The evolution of animal husbandry in Neolithic central-west Anatolia: the zooarchaeological record from Ulucak Höyük (c. 7040–5660 cal. BC, Izmir, Turkey). *Anatolian Studies* 62:1–33.

297. Zeder MA (1994) After the revolution: post-Neolithic subsistence in northern Mesopotamia. *Am Anthropol* 96(1):97–126.

298. Church MJ, et al. (2005) Puffins, Pigs, Cod and Barley: Palaeoeconomy at Undir Junkarinsfløtti, Sandoy, Faroe Islands. *Environ Archaeol* 10(2):179–197.

299. Arge SV, Church MJ, Brewington SD (2009) Pigs in the Faroe Islands: An Ancient Facet of the Islands' Paleoeconomy. *Journal of the North Atlantic*:19–32.

300. Brewington S, et al. (2015) Islands of change vs. islands of disaster: Managing pigs and birds in the Anthropocene of the North Atlantic. *Holocene* 25(10):1676–1684.

301. Caduff B (2005) Die ur-und frühgeschichtlichen Fundstellen auf dem Haselboden in Untervaz (GR). *Archäologie der Schweiz* 28(3):16–23.

302. Comşa E (1961) Săpăturile arheologice de la Boian. *Materiale şi cercetări arheologice* 7:63–70.

303. Bolomey A (1966) Fauna neolitica din asezarea Boian A de la Varasti. *Studii si cercetari de antropologie* 3(1):27–34.

304. Volante N (2003) Neto-Via Verga (Sesto Fiorentino): la produzione vascolare dell'area 1. *Rivista di scienze preistoriche* (53):375–504.

305. Sarti L, Volante N (2011) Il pieno Neolitico in Toscana: variabilità delle produzioni ceramiche e litiche nel contesto dell'Italia centrale. *Rivista di studi liguri* (77):497–504.

306. Coradeschi G (2013) O sítio da Idade do Bronze de Via Neruda em Sesto Fiorentino (Florença, Itália): exploração dos recursos arbóreos. *Techne* 1(1).

307. Tasić NN, Ignjatović M (1908) Od tradicionalne do moderne metodologije. Istraživanja u Vinči 1978--2008. *Vinča—praistorijska metropola, istraživanja* 2008:87–119.

308. Dimitrijević V (2006) Vertebrate fauna of Vinča-Belo Brdo: Excavation campaigns 1998-2003. *Starinar* (56):245–269.

309. Andreescu R, Mirea P, Apope Ş (2001) Cultura Gumelniţa în vestul Munteniei. Aşezarea de la Vităneşti, jud Teleorman. O Civilizaţie Necunoscută. Gumelniţa.

310. Balasse M, et al. (2016) Wild, domestic and feral? Investigating the status of suids in the Romanian Gumelniţa (5th mil. cal BC) with biogeochemistry and geometric morphometrics. *Journal of Anthropological Archaeology* 42:27–36.

311. Bréhard S, Bălăşescu A (2012) What's behind the tell phenomenon? An archaeozoological approach of Eneolithic sites in Romania. *J Archaeol Sci* 39(10):3167–3183.

312. Srejović D, Letica Z (1978) *Vlasac: A mesolithic settlement in the iron gates* (Serbian Academy of Science and Art).

313. Borić D, et al. (2014) Late Mesolithic lifeways and deathways at Vlasac (Serbia). *J Field Archaeol* 39(1):4–31.

314. Berglund J (1982) Kirkebjerget—A Late Bronze Age Settlement at Voldtofte, South-West Funen: An Interim Report on the Excavations of 1976 and 1977. *Journal of Danish Archaeology* 1(1):51–63.

315. Barker P, White R, Pretty K, Bird H, and Corbishley M (1997) *The Baths Basilica Wroxeter: Excavations 1966–90* (English Heritage, London).

316. Hammon A (2011) Understanding the Romano-British–Early Medieval Transition: A Zooarchaeological Perspective from Wroxeter (Viroconium Cornoviorum). *Britannia* 42:275–305.

317. Albarella U, Davis S, Detry C, Rowley-Conwy P (2005) Pigs of the "Far West": the biometry of Sus from archaeological sites in Portugal. *Anthropozoologica* 40:27–54.

318. Pyzel J (2001) *Naczynie mieszkowate kultury ceramiki wstęgowej rytej z Żegotek, gm. Strzelno, woj. kujawsko-pomorskie, stan. 2* (Instytut Archeologii i Etnologii Polskiej Akademii Nauk, Kraków).

319. Schibler J (1997) *Ökonomie und Ökologie neolithischer und bronzezeitlicher Ufersiedlungen am Zürichsee: Ergebnisse der Ausgrabungen Mozartstrasse, Kanalisationssanierung Seefeld, AKAD/Pressehaus und Mythenschloss in Zürich* (Direktion der Öffentlichen Bauten des Kantons Zürich, Hochbauamt, Abt. Kantonsarchäologie).

320. Ebersbach R, Ruckstuhl B, Bleicher N (2015) Zürich "Mozartstrasse". Neolithische und bronzezeitliche Ufersiedlungen. Band 5: Die neolithischen Befunde und die Dendroarchäologie. *Monographien Der Kantonsarchäologie Zürich 47* (Zürich und Egg).

321. Larson G, et al. (2007) Ancient DNA, pig domestication, and the spread of the Neolithic into Europe. *Proc Natl Acad Sci U S A* 104(39):15276–15281.

322. Binladen J, et al. (2007) The use of coded PCR primers enables high-throughput sequencing of multiple homolog amplification products by 454 parallel sequencing. *PLoS One* 2(2):e197.

323. Dabney J, et al. (2013) Complete mitochondrial genome sequence of a Middle Pleistocene cave bear reconstructed from ultrashort DNA fragments. *Proc Natl Acad Sci U S A* 110(39):15758–15763.

324. Damgaard PB, et al. (2015) Improving access to endogenous DNA in ancient bones and teeth. *Sci Rep* 5:11184.

325. Meyer M, Kircher M (2010) Illumina sequencing library preparation for highly multiplexed target capture and sequencing. *Cold Spring Harb Protoc* 2010(6):db.prot5448.

326. Rohland N, Harney E, Mallick S, Nordenfelt S, Reich D (2015) Partial uracil-DNA-glycosylase treatment for screening of ancient DNA. *Philos Trans R Soc Lond B Biol Sci* 370(1660):20130624.

327. Korlević P, et al. (2015) Reducing microbial and human contamination in DNA extractions from ancient bones and teeth. *Biotechniques* 59(2):87–93.

328. Svensson EM, et al. (2007) Tracing genetic change over time using nuclear SNPs in ancient and modern cattle. *Anim Genet* 38(4):378–383.

329. Briggs AW, Heyn P (2012) Preparation of Next-Generation Sequencing Libraries from Damaged DNA. *Ancient DNA: Methods and Protocols*, eds Shapiro B, Hofreiter M (Humana Press, Totowa, NJ), pp 143–154.

330. Li H, Durbin R (2009) Fast and accurate short read alignment with Burrows-Wheeler transform. *Bioinformatics* 25(14):1754–1760.

331. Schubert M, et al. (2012) Improving ancient DNA read mapping against modern reference genomes. *BMC Genomics* 13(1):178.

332. Li H, et al. (2009) The Sequence Alignment / Map (SAM) Format and SAMtools 1000 Genome Project Data Processing Subgroup. *Data Processing*:1–2.

333. Kircher M (2012) Analysis of high-throughput ancient DNA sequencing data. *Methods Mol Biol* 840:197–228.

334. Quinlan AR, Hall IM (2010) BEDTools: a flexible suite of utilities for comparing genomic features. *Bioinformatics* 26(6):841–842.

335. Skoglund P, Ersmark E, Palkopoulou E, Dalén L (2015) Ancient wolf genome reveals an early divergence of domestic dog ancestors and admixture into high-latitude breeds. *Curr Biol* 25(11):1515–1519.

336. Jónsson H, Ginolhac A, Schubert M, Johnson PLF, Orlando L (2013) mapDamage2.0: fast approximate Bayesian estimates of ancient DNA damage parameters. *Bioinformatics* 29(13):1682–1684.

337. Frantz LAF, et al. (2015) Evidence of long-term gene flow and selection during domestication from analyses of Eurasian wild and domestic pig genomes. *Nat Genet* 47(10):1141–1148.

338. Bosse M, et al. (2014) Genomic analysis reveals selection for Asian genes in European pigs following human-mediated introgression. *Nat Commun* 5:4392.

339. Alexandri P, et al. (2012) The Balkans and the colonization of Europe: the post-glacial range expansion of the wild boar, Sus scrofa : Post-glacial range expansion of wild boar. *J Biogeogr* 39(4):713–723.

340. Frantz LAF, et al. (2013) Genome sequencing reveals fine scale diversification and reticulation history during speciation in Sus. *Genome Biol* 14(9):R107.

341. Stamatakis A (2006) RAxML-VI-HPC: maximum likelihood-based phylogenetic analyses with thousands of taxa and mixed models. *Bioinformatics* 22(21):2688–2690.

342. Larson G, et al. (2005) Worldwide phylogeography of wild boar reveals multiple centers of pig domestication. *Science* 307(5715):1618–1621.

343. Sukumaran J, Holder MT (2010) DendroPy: a Python library for phylogenetic computing. *Bioinformatics* 26(12):1569–1571.

344. Larson G, et al. (2007) Phylogeny and ancient DNA of Sus provides insights into neolithic expansion in Island Southeast Asia and Oceania. *Proc Natl Acad Sci U S A* 104(12):4834–4839.

345. Frantz L, et al. (2018) Synchronous diversification of Sulawesi’s iconic artiodactyls driven by recent geological events. *bioRxiv*:241448.

346. Caye BK, Jay F, Michel O, François O (2016) Fast Inference of Individual Admixture Coefficients Using Geographic Data. *bioRxiv*:080291.

347. Martins H, Caye K, Luu K, Blum MGB, François O (2016) Identifying outlier loci in admixed and in continuous populations using ancestral population differentiation statistics. *bioRxiv*:054585.

348. Caye K, Deist TM, Martins H, Michel O, François O (2016) TESS3: fast inference of spatial population structure and genome scans for selection. *Mol Ecol Resour* 16(2):540–548.

349. Khalilzadeh P, et al. (2016) Contact Zone of Asian and European Wild Boar at North West of Iran. *PLoS One* 11(7):e0159499.

350. Krause-Kyora B, et al. (2013) Use of domesticated pigs by Mesolithic hunter-gatherers in northwestern Europe. *Nat Commun* 4:2348.

351. Caliebe A, Nebel A, Makarewicz C, Krawczak M, Krause-Kyora B (2017) Insights into early pig domestication provided by ancient DNA analysis. *Sci Rep* 7:44550.

352. Fang M, Berg F, Ducos A, Andersson L (2006) Mitochondrial haplotypes of European wild boars with 2n = 36 are closely related to those of European domestic pigs with 2n = 38. *Anim Genet* 37(5):459–464.

353. Fang M, Larson G, Ribeiro HS, Li N, Andersson L (2009) Contrasting mode of evolution at a coat color locus in wild and domestic pigs. *PLoS Genet* 5(1):e1000341.

354. Koutsogiannouli EA, Moutou KA, Sarafidou T, Stamatis C, Mamuris Z (2010) Detection of hybrids between wild boars (Sus scrofa scrofa) and domestic pigs (Sus scrofa f. domestica) in Greece, using the PCR-RFLP method on melanocortin-1 receptor (MC1R) mutations. *Mamm Biol* 75(1):69–73.

355. Frantz AC, Zachos FE, Kirschning J (2013) Genetic evidence for introgression between domestic pigs and wild boars (Sus scrofa) in Belgium and Luxembourg: a comparative approach with multiple marker …. *Biol J Linn Soc Lond* 110(1):104–11.

356. Schliep KP (2011) phangorn: phylogenetic analysis in R. *Bioinformatics* 27(4):592–593.

357. Goedbloed DJ, et al. (2013) Genome-wide single nucleotide polymorphism analysis reveals recent genetic introgression from domestic pigs into Northwest European wild boar populations. *Mol Ecol* 22(3):856–866.

358. Purcell S, et al. (2007) PLINK: a tool set for whole-genome association and population-based linkage analyses. *Am J Hum Genet* 81(3):559–575.

359. Paradis E, Claude J, Strimmer K (2004) APE: Analyses of Phylogenetics and Evolution in R language. *Bioinformatics* 20(2):289–290.

360. Patterson N, et al. (2012) Ancient admixture in human history. *Genetics* 192(3):1065–1093.

361. Patterson N, Price AL, Reich D (2006) Population structure and eigenanalysis. *PLoS Genet* 2(12):e190.

362. Groenen MAM, et al. (2012) Analyses of pig genomes provide insight into porcine demography and evolution. *Nature* 491(7424):393–398.

363. White S (2011) From Globalized Pig Breeds to Capitalist Pigs: A Study in Animal Cultures and Evolutionary History. *Environ Hist Durh N C* 16(1):94–120.

364. Alexander DH, Novembre J, Lange K (2009) Fast model-based estimation of ancestry in unrelated individuals. *Genome Res* 19(9):1655–1664.

365. Falush D, van Dorp L, Lawson D (2016) A tutorial on how (not) to over-interpret STRUCTURE/ADMIXTURE bar plots. *bioRxiv*:066431.

366. Hellenthal G, et al. (2014) A genetic atlas of human admixture history. *Science* 343(6172):747–751.

367. O'Connell J, et al. (2014) A general approach for haplotype phasing across the full spectrum of relatedness. *PLoS Genet* 10(4):e1004234.

368. Tortereau F, et al. (2012) A high density recombination map of the pig reveals a correlation between sex-specific recombination and GC content. *BMC Genomics* 13(1):586.

369. Lawson DJ, Hellenthal G, Myers S, Falush D (2012) Inference of population structure using dense haplotype data. *PLoS Genet* 8(1):e1002453.

370. Leslie S, et al. (2015) The fine-scale genetic structure of the British population. *Nature* 519(7543):309–314.

371. Bosse M, et al. (2014) Untangling the hybrid nature of modern pig genomes: a mosaic derived from biogeographically distinct and highly divergent Sus scrofa populations. *Mol Ecol* 23(16):4089–4102.

372. Fang M, Andersson L (2006) Mitochondrial diversity in European and Chinese pigs is consistent with population expansions that occurred prior to domestication. *Proc Biol Sci* 273(1595):1803–1810.

373. Price AL, et al. (2009) Sensitive detection of chromosomal segments of distinct ancestry in admixed populations. *PLoS Genet* 5(6):e1000519.

374. Ai H, et al. (2015) Adaptation and possible ancient interspecies introgression in pigs identified by whole-genome sequencing. *Nat Genet* 47(3):217–225.

375. Cruickshank TE, Hahn MW (2014) Reanalysis suggests that genomic islands of speciation are due to reduced diversity, not reduced gene flow. *Mol Ecol* 23(13):3133–3157.
